# Perovskite Oxide Materials for Solar Thermochemical Hydrogen Production from Water Splitting through Chemical Looping

**DOI:** 10.1021/acscatal.4c03357

**Published:** 2024-09-25

**Authors:** Cijie Liu, Jiyun Park, Héctor A. De Santiago, Boyuan Xu, Wei Li, Dawei Zhang, Lingfeng Zhou, Yue Qi, Jian Luo, Xingbo Liu

**Affiliations:** † Department of Mechanical, Materials and Aerospace Engineering, Benjamin M. Statler College of Engineering and Mineral Resources, West Virginia University, Morgantown, West Virginia 26506, United States; ‡ School of Engineering, Brown University, 184 Hope Street, Providence, Rhode Island 02912, United States; § Department of Physics, Brown University, 184 Hope Street, Providence, Rhode Island 02912, United States; ∥ Program in Materials Science and Engineering, University of California San Diego, La Jolla, California 92093, United States; ⊥ Department of Chemical and Biomedical Engineering, Benjamin M. Statler College of Engineering and Mineral Resources, West Virginia University, Morgantown, West Virginia 26506, United States; # Department of NanoEngineering, University of California San Diego, La Jolla, California 92093, United States

**Keywords:** chemical looping, solar-driven thermochemical hydrogen (STCH), thermodynamics and kinetics, renewable energy

## Abstract

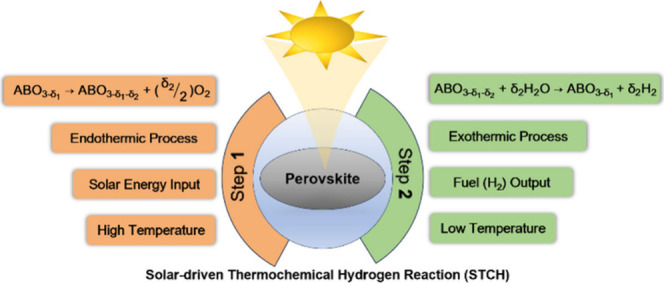

Solar-driven thermochemical hydrogen (STCH) production represents a sustainable approach for converting solar energy into hydrogen (H_2_) as a clean fuel. This technology serves as a crucial feedstock for synthetic fuel production, aligning with the principles of sustainable energy. The efficiency of the conversion process relies on the meticulous tuning of the properties of active materials, mostly commonly perovskite and fluorite oxides. This Review conducts a comprehensive review encompassing experimental, computational, and thermodynamic and kinetic property studies, primarily assessing the utilization of perovskite oxides in two-step thermochemical reactions and identifying essential attributes for future research endeavors. Furthermore, this Review delves into the application of machine learning (ML) and density functional theory (DFT) for predicting and classifying the thermochemical properties of perovskite materials. Through the integration of experimental investigations, computational modeling, and ML methodologies, this Review aspires to expedite the screening and optimization of perovskite oxides, thus enhancing the efficiency of STCH processes. The overarching objective is to propel the advancement and practical integration of STCH systems, contributing significantly to the realization of a sustainable and carbon-neutral energy landscape.

## 1 Introduction

Solar energy has emerged as a crucial and indispensable energy source, owing to its renewable, abundant, inexhaustible, and environmentally friendly attributes.^1−4^ However, the intermittent nature of solar energy, confined to daylight hours and contingent on weather conditions, poses a challenge to its wide utilization.^5,6^ To overcome this limitation, researchers are dedicatedly working on the development of efficient energy conversion technologies that facilitate the maximum usage of solar energy even during nonsolar periods.^7−9^ Currently, diverse technologies, encompassing both artificial and natural photosynthesis and photoelectrochemistry (PEC), are being explored for hydrogen production through water (H_2_O) splitting (WS), carbon monoxide (CO) generation through carbon dioxide (CO_2_) splitting (CDS), and the synthesis of versatile fuels from biomass.^10,11^ Particularly intriguing are technologies that enable the efficient dissociation of H_2_O and CO_2_ through endothermic processes, where the product of H_2_ and CO can be further transformed into organic oxygenates and various liquid hydrocarbons.^12−14^ Another technology rapidly gaining attention is the conversion of solar energy into fuel through solar-driven thermochemical processes.^15,16^

**Figure 1:**
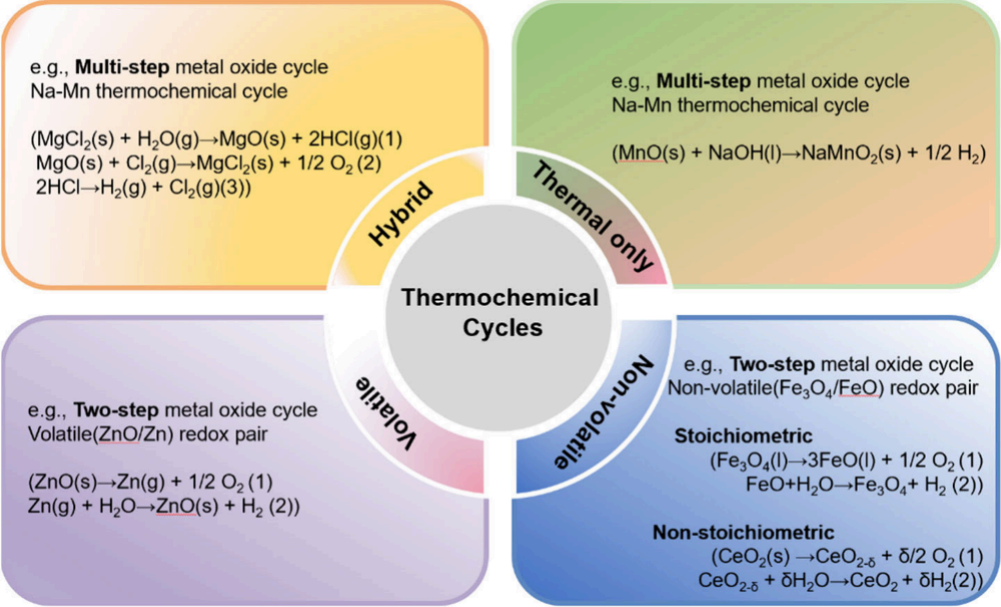
Examples of different types of thermochemical reactions.

The traditional solar thermochemical technique entails a two-phase redox cycle utilizing ceria or perovskite materials, involving high-temperature reduction followed by oxidation. This Review delineates two main types of thermochemical cycles: multistep and two-step, with specific examples, as illustrated in Figure 1. The magnesium–chlorine (MgCl) cycle is a multistep hybrid process incorporating both electricity and heat to split water, comprising one electrolytic step and two thermochemical steps.^17^ An example of the multistep thermal-only type includes manganese oxide–sodium hydroxide (MnO-NaOH), first investigated in 1999 by Sturzenegger et al.^18^ However, challenges related to multiple stages, such as high reduction temperatures, significant temperature fluctuations, and complex system integration, have stimulated the exploration of simpler two-step redox reactions utilizing basic metal oxides.^19^ Two-step redox metal oxides are classified into volatile and nonvolatile categories. The former underwent extensive investigation from 2000 to 2010, presenting challenges in recovering condensed metallic gas species (e.g., Zn, Sn) in colder reactor sections.^20,21^ The nonvolatile category encompasses stoichiometric and nonstoichiometric metal oxides. However, stoichiometric examples like iron oxide often encounter sintering issues leading to the formation of a deactivation layer of Fe_3_O_4_.^22^ In the nonstoichiometric series, ceria stands out for its rapid oxidation kinetics, with 500 cycle reactions demonstrating promise. Despite its advantages, ceria has drawbacks, including a low degree of nonstoichiometry and the need for high reduction temperatures.^12^ CeO_2_ and CeO_2_-based fluorite oxides have been widely reported, with CeO_2_ commonly used as the benchmark material.^23,24^ Herein, perovskite is considered a promising candidate due to its requirement of a low reduction temperature and large compositional design space. Additionally, perovskite exhibits significant nonstoichiometry, fast oxidation kinetics in most cases, and the ability to undergo testing across hundreds of cycles.^25^

**Figure 2:**
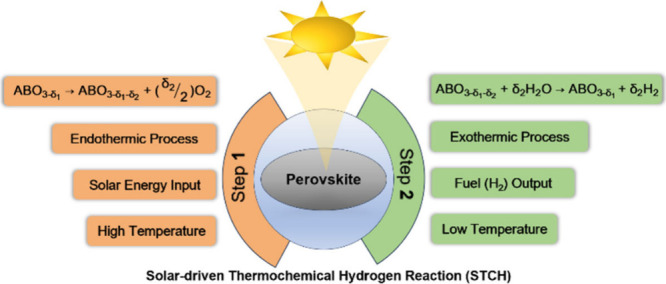
Schematic of the two-step water-splitting reaction using a metal oxide. In step 1, the perovskite oxide is reduced, accompanied by oxygen evolution, at high temperatures. In step 2, the perovskite is reoxidized, involving H_2_ evolution, at low temperatures.

The two-step thermochemical water-splitting method is universally recognized as a thermodynamically viable and potentially highly efficient process for generating “solar fuels”.^26^ Unlike other methods that depend on photon-to-electron conversion, such as electrolysis, this technology operates at elevated temperatures and has the advantage of utilizing the entire solar spectrum.^27^ Chemical looping refers to a process used in solar thermochemical systems for energy conversion and storage.^28^ Thermochemical reactions in this context comprise two processes: Process 1 involves the reduction of perovskite oxide under solar energy (eq 1), with the production of oxygen (O_2_). Process 2 encompasses the reoxidation of the reduced perovskite oxide, leading to the evolution of fuel (H_2_) (eq 2). The specific steps are illustrated in Figure 2 by considering perovskite oxide (ABO_3_) in the context of solar-driven thermochemical hydrogen production.
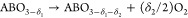
1
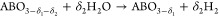
2Here, δ_1_ represents the initial reduction extent and δ_2_ denotes the additional reduction extent due to heating during process 1.

Solar thermochemical hydrogen (STCH) production has been extensively reviewed in the literature. Various reviews have covered the basic theory of thermodynamics and kinetics, different types of materials, and various reactor technologies.^29−33^ For example, Aldo Steinfeld’s group reviewed the use of concentrated solar radiation for high-temperature process heat, the thermodynamics of various metal oxide redox reactions, and the design and operation of solar reactors.^34−37^ Some reviews have focused on specific materials, such as ceria and perovskite, used in the STCH field. For example, Stéphane Abanades’ group summarized advanced redox cycles involving various metal oxides.^38,39^ They highlighted the ability of these materials to produce water with high H_2_ production yields, rapid reaction rates, and performance stability. Moreover, STCH as a H_2_ generation method is also used to make comparisons with other methods, including PEC; for instance, Chengxiang Xiang’s group has compared solar thermochemical and photoelectrochemical water-splitting methods, illustrating their respective advantages and challenges.^40^ Despite these extensive studies, there is still a lack of a comprehensive summary of various B-site-based materials used in the STCH reaction, particularly regarding their thermodynamic and kinetic properties. This Review seeks to illuminate the landscape of STCH reactions, emphasizing the potential of perovskite materials from the perspectives of experimental, computational, thermodynamic, and kinetic studies as well as the complexities they entail. Ultimately, these efforts hold the potential not only to shape a more sustainable energy future but also to extend into the research field of chemical looping.

## 2 Structure and Properties of Perovskite Oxides

**Figure 3:**
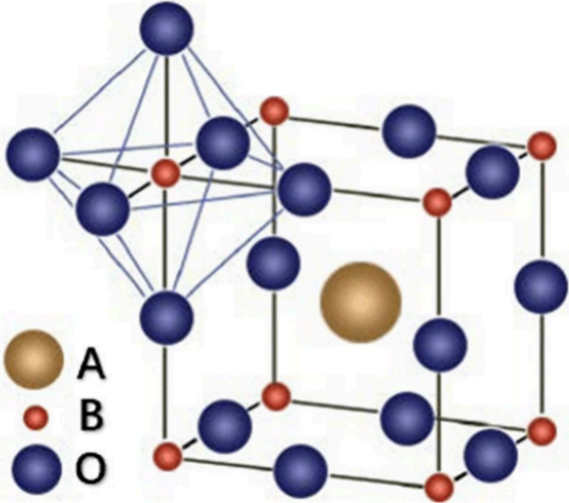
In the ideal perovskite structure, an octahedron of O atoms encloses the B ion, forming a three-dimensional cubic lattice through corner sharing, with the B-site ion situated at the center and surrounded by oxygen ions.^45^ Reproduced from ref (45) with permission from the research repository at WVU. Copyright 2022 West Virginia University.

Perovskite-type oxides are named after CaTiO_3_ and belong to a class of oxides with the common formula ABO_3_ (where A represents a rare- or alkaline-earth metal and B represents a transition metal). In perovskite oxides, the crystal structure is characterized by a dominant 12-fold A cation sublattice, a 6-fold coordinated B cation sublattice, and an octahedral oxygen anion sublattice positioned between two B site locations. Furthermore, the cations in the A-site are larger than those in the B-site, as depicted in Figure 3.^41−44^

Inorganic perovskites, owing to their unique physical properties such as superconductivity, magnetoresistance, dielectricity, piezoelectricity, and ferroelectricity, can be applied in a variety of fields.^46^ These fields include fuel cells, catalysis, thermoelectrics, batteries, and solar thermal energy, emphasizing their importance as energy materials.^47−51^ When employed in solar thermal energy applications, perovskite oxides undergo thermal reduction, releasing oxygen from the lattice and creating oxygen vacancies. This process also induces changes in the oxidation states of the multivalent transition metal cations present in the structure.

To maintain the perovskite phase, Victor Moritz Goldschmidt developed a tolerance factor based on the ionic radii of the cations occupying the A- and B-sites, and it serves as an important parameter in assessing perovskite structures. This so-called Goldschmidt tolerance factor, τ, is a function of the A cation radii, *R*_A_, the B cation radii, *R*_B_, and the O anion radii, *R*_O_.^52^
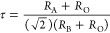
3In general, materials with an ideal cubic structure have a tolerance factor (τ) within the range of 0.9 ≤ τ ≤ 1.0, while a hexagonal/tetragonal phase may form when τ > 1.0, and an orthorhombic/rhombohedral phase may form when τ < 0.9.^53−56^ Distortions in perovskite structures can result in a variety of symmetry reductions, with orthorhombic and rhombohedral distortions being the most common.^57,58^ Considering the most practical perovskite materials have mixed A and B ions, the weighted average cation radius is typically used and is expressed as follows:^59−62^
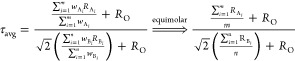
4where the subscript *i* indicates different types of cations (*i* = 1, 2, ..., *m* for A-site cations and *i* = 1, 2, ..., *n* for B-site cations), and *w*_A_*i*__ and *w*_B_*i*__ represent atomic fractions associated with *R*_A_*i*__ and *R*_B_*i*__, respectively. Prior experiments suggested that the Goldschmidt tolerance factor needs to be closer to unity (e.g., 0.99 ≤ τ ≤ 1.01) for the formation of single-phase cubic high-entropy perovskite oxides.^62^

Despite the Goldschmidt tolerance factor’s simplicity and utility in predicting perovskite stability, Christopher J. Bartel et al. developed an improved tolerance factor using a data-driven approach, which correctly predicts perovskite stability with 92% accuracy on a diverse set of 576 compounds.^63^ This new factor accounts for the limitations of the traditional Goldschmidt factor, particularly in handling a broader range of ABX compositions, including halides and oxides. Incorporating these recent advancements allows for a more reliable prediction of perovskite stability and guides the discovery of new functional materials for various applications, including photovoltaics and electrocatalysis.

The ability to modify the perovskite structure provides extensive opportunities for enhancing the material properties, such as electronic and ionic conductivity.^64,65^ When considering perovskites for thermochemical cycles, their reactivity for oxygen exchange and H_2_O/CO_2_ splitting is an important determining factor. To be suitable for this application, a perovskite material should exhibit a large range of oxygen nonstoichiometry (δ_2_), possess a well-matched enthalpy of formation for oxide defects (typically oxygen vacancies), and support mobile electronic carriers that facilitate the exchange reaction at the surface.^58^

## 3 Thermodynamics of the Thermochemical Water-Splitting Reduction Cycle

By examining the thermodynamics of perovskites, we can predict the theoretical maximum achievable fuel production achievable in thermochemical cycles as a function of reduction and oxidation temperatures and the partial pressure of oxygen. This Δ*G*_O_^°^ can be calculated using the standard enthalpy change (Δ*H*_O_^°^) and the standard entropy change (Δ*S*_O_^°^) according to the following equation:^25^
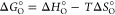
5These relationships were formulated under the assumption that the individual values of Δ*G*_O_^°^ and Δ*S*_O_^°^ remain temperature-independent (*T*) and are solely reliant on the extent of nonstoichiometry (δ_2_).^29^ This presumption is reasonable for minor fluctuations in δ_2_ and when the crystal structure is maintained. Considering that oxygen behaves as an ideal gas and the solid activity is unity, the Δ*G*_O_^°^ at equilibrium can be determined as follows:
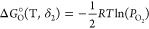
6The thermodynamics of active materials represent their most crucial attributes, which are essential for making unbiased comparisons between different materials and in system models. Hence, obtaining accurate thermodynamic data is of paramount importance. In the literature, one primary method is prevalent for calculating thermodynamic properties of materials used in solar thermochemical hydrogen (STCH) production: the van’t Hoff analysis.^66^

The thermodynamics of reduction parameters (the reduction enthalpy change (Δ*H*_red_^°^) and the reduction entropy change (Δ*S*_red_^°^)) of perovskite can be measured by thermogravimetric analysis (TGA) according to the equation below:^67,68^
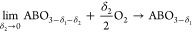
7According to the van’t Hoff method, in the limit of an infinitesimal extent of reduction, the chemical activities of ABO_3−δ_1__ and ABO_3−δ_1_–δ_2__ become equal. Therefore, the equilibrium reaction constant (*K*_red_^eq^) can be given by
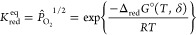
8where *P̂*_O_2__ is the *P*_O_2__ relative to the reference standard pressure of 1 atm. The Arrhenius plot of eq 8 can be written in the form
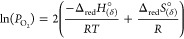
9Δ_red_*H*_(δ)_^°^ and Δ_red_*S*_(δ)_^°^ can be extracted from the slopes and intercepts of the plots of ln(*P*_O_2__) vs 1/*T* for each δ, respectively.

**Figure 4:**
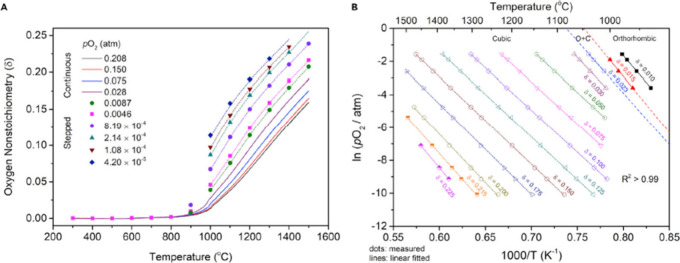
The process for performing the van’t Hoff method is illustrated as follows: (a) a map of temperature, pressure, and oxygen nonstoichiometry for CaTi_0.5_Mn_0.5_O_3−δ_ and (B) straight lines that are linear fits to the plotted data points.^66^ Reproduced from ref (66). Available under a Creative Commons CC-BY-NC-ND license. Copyright 2020 Qian and co-workers.

According to the van’t Hoff method, a graph is constructed in the Arrhenius form, plotting the set of values for oxygen partial pressure against corresponding temperatures for a specified value of δ. From this graph, the slope and y-intercept can be determined, which correspond to the enthalpy and entropy, respectively. Figure 4, which illustrates an instance of this analysis for the perovskite oxide CaTi_0.5_Mn_0.5_O_3−δ_, clearly displays a minor anomaly at a temperature of 980 °C. This feature indicates a transition to a cubic phase, an attribute also observed in the oxide CaMnO_3−δ_.^66,69,70^

**Figure 5:**
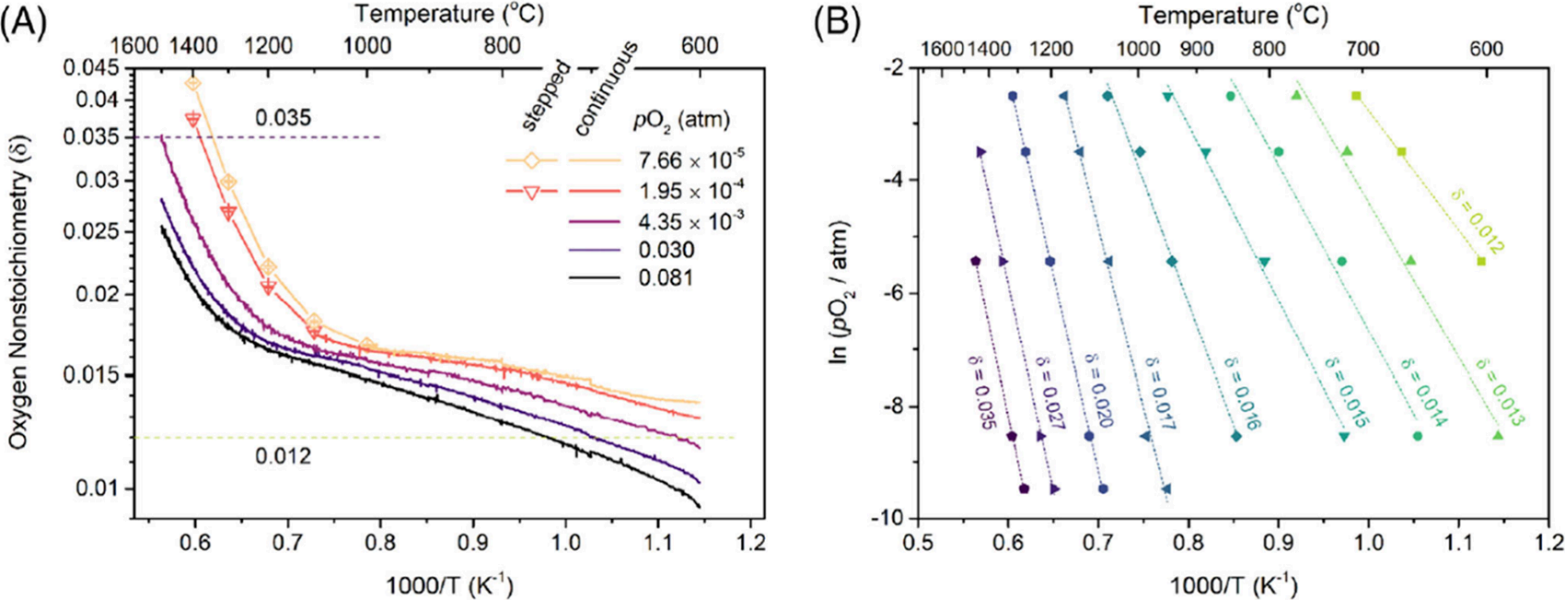
(A) Oxygen nonstoichiometry measured using both continuous and stepwise heating at the indicated *P*_O_2__ values. (B) An Arrhenius presentation is used to extract thermodynamic properties using the van’t Hoff method, with representative fixed δ values ranging between 0.012 and 0.035.^71^ Reproduced with permission from ref (71). Copyright 2022 John Wiley and Sons.

Xin Qian et al. first reported the experimental protocols for evaluating the redox thermodynamics of nonstoichiometric oxides, focusing on YMnO_3−δ_, as depicted in Figure 5.^71^ They employed a temperature-stepped protocol involving long isothermal holds. Interpolation was necessary for analyzing the results obtained from these stepped measurements, with a preference for ln(δ) versus 1/*T* plane analysis over δ versus *T*.^71^

The following equation is given to consider the influence of temperature at the first thermolysis reactions:^72^
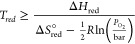
10In the given conditions, Δ*H*_red_ is approximately 250 kJ mol_o_^–1^ and Δ*S*_red_^°^ is nearly constant at around 57 J (mol_o_ K) ^–1^ in the reduction process of water-splitting. At fixed *P*_O_2__, *T*_red_ can be reduced only by either decreasing Δ*H*_red_ or increasing Δ*S*_red_^°^. However, a large Δ*H*_red_ is required to provide the necessary driving force for the reduction of ABO_3_. Therefore, a large Δ*S*_red_^°^ holds a pivotal role in the success of materials for thermochemical splitting.

The Δ*S*_red_^°^ may be decomposed into two parts: (1) a gas-phase contribution arising from the release of half a mole of O_2_ and (2) a solid-state contribution (or more broadly, an oxide/metal contribution) indicating the entropy variation in the oxide between its oxidized and reduced states.^72^
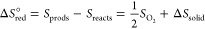
11In this condition,
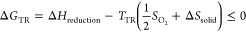
12and
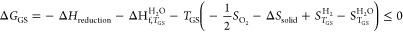
13The van’t Hoff model is extensively employed in solar thermochemical materials due to its relatively straightforward application. Generally, there exists considerable flexibility between the Δ*H*_red_ and Δ*S*_red_^°^ terms, leading to the potential misallocation of energies between enthalpy and entropy terms. Therefore, while nonstoichiometry might be predicted with accuracy, achieving precision in the determination of partial molar entropies and enthalpies could be challenging. Moreover, employing linear regression within the van’t Hoff space might result in inaccuracies, particularly when dealing with a narrow temperature range. The inherent 1/*T* nature of this transformation leads to a concentration of higher-temperature data points, potentially amplifying experimental errors during the determination of the slope (Δ*H*_red_) and intercept (Δ*S*_red_^°^). As a result, its application should prioritize broader temperature ranges and encompass an investigation into nonstoichiometry at lower temperatures.^27^

**Figure 6:**
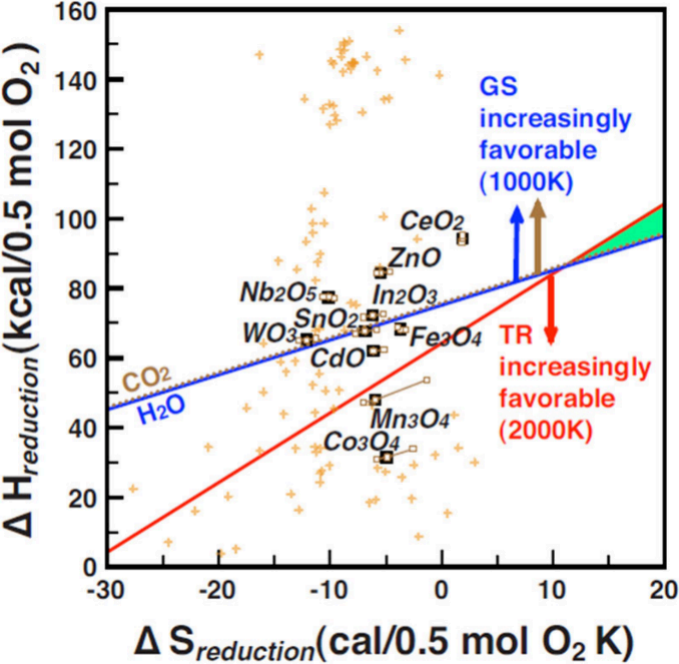
Computational screening for the utilization of monometal oxides in thermochemical water-splitting was conducted by C. Wolverton et al.^73^ The screening encompassed considerations of oxidation and reduction toward equilibrium as well as reduction under atmospheric pressures. The “ideal” materials would be positioned within the designated green triangle region.^27^ Reproduced with permission from ref (73). Copyright 2009 American Physical Society.

Computational methods can be utilized to predict the thermodynamics of proposed water-splitting cycles after the materials are used in two-step water-splitting cycles. C. Wolverton’s group screened all binary oxides (CeO_2_, ZnO, Nb_2_O_5_, In_2_O_3_, SnO_2_, WO_3_, Fe_3_O_4_, CdO, Mn_3_O_4_, and Co_3_O_4_) for use in thermochemical H_2_O splitting, as depicted in Figure 6.^27,73^ They discovered that these oxides tend to aggregate close to the thermal reduction (TR) and gas splitting (GS) equilibrium lines. It is reasonable that most oxides also prefer GS slightly because the GS reaction is more likely affected by slow kinetics. Figure 6 shows that none of the evaluated reactions achieve the requisite combination of Δ*H*_red_ and Δ*S*_red_^°^ necessary to facilitate favorable TR and GS energetics. However, it is noted that Δ*S*_red_^°^ serves as a means to create an optimal range for values exceeding 10 cal (0.5 mol O_2_ K)^−1^. Achieving a substantial positive Δ*S*_red_^°^ is a complex task due to the fact that MO_*x*–1_ contains fewer atoms compared to MO_*x*_, resulting in fewer vibrational degrees of freedom. Additionally, based on eqs 12 and 13, identifying or developing materials that possess a substantial positive Δ*S*_red_^°^ and also exhibit a corresponding Δ*H*_red_ is an ongoing challenge.

## 4 Kinetics of the Thermochemical Water-Splitting Cycle

Theoretical limits on fuel production are determined by thermodynamic restrictions, while kinetic restrictions dictate the maximum quantity of fuel that can be generated within a reasonable duration. Here, the common methods used to investigate the kinetics of redox reactions involving perovskites will be discussed. The H_2_ output depends on thermodynamics, the reaction kinetics, the mass transport, etc.; therefore, it highly depends on the measurement protocol.

The rate of a solid-state reaction can generally be described by^74,75^
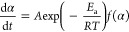
14where α represents the conversion fraction, *A* stands for the pre-exponential (frequency) factor, *E*_a_ denotes the activation energy, *R* signifies the gas constant, and *T* represents the absolute temperature. The function *f*(α) represents the reaction model. For a gravimetric measurement, α can be defined by the following equation:^74^
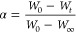
15where *W*_0_ is the initial weight, the *W*_*t*_ is the weight at time *t*, and *W*_∞_ is the final weight.

Jonathan R. Scheffe et al. were the first to introduce a computational method for analyzing the transient H_2_ production rate observed during water-splitting. This method takes the following into account: (1) the kinetics inherent to the solid state, (2) the time needed to infuse the reacting chamber with water vapor, (3) delays associated with the detector, and (4) the distribution and blending of H_2_ as it moves from the solid surface and travels downstream to the measurement device. Many earlier studies overlooked the significance of the experimental nuances (specifically points 2–4), and this oversight could result in inaccurate findings.^76^

**Figure 7:**
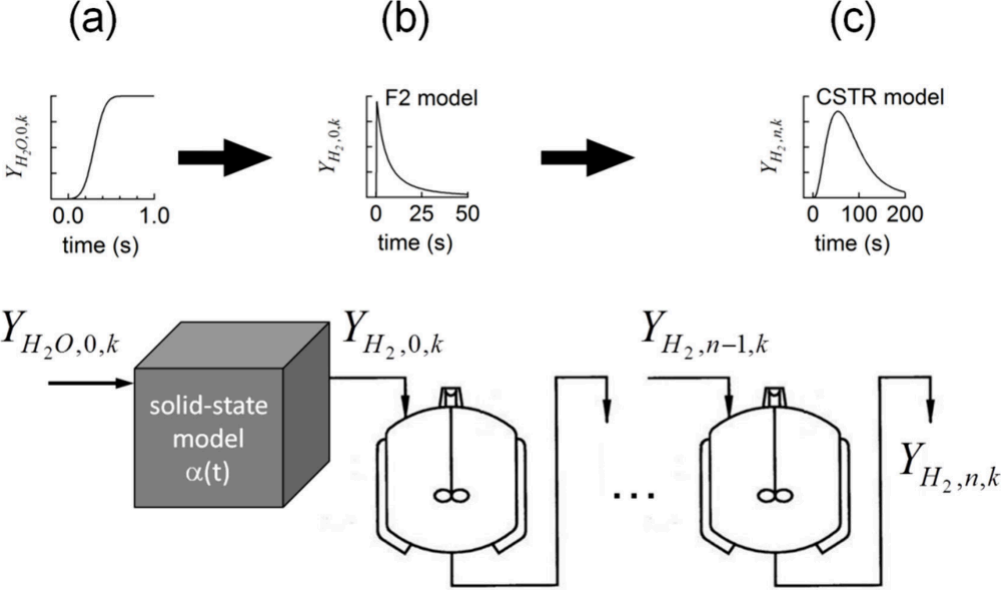
The kinetic model employed by Jonathan et al.^76^ is schematically depicted as follows: (a) a step function representing the introduction of steam, (b) an H_2_ evolution curve derived from the solid-state model, and (c) the H_2_ flow rate following its mixing and dispersion within a continuously stirred tank reactor (CSTR). Reproduced with permission from ref (76). Copyright 2013 RSC Publishing.

A. H. McDaniel et al. also employed this computational approach, using water-splitting as an example of oxidation chemistry. They introduced steam into the reactor volume through a step-function input that triggers the solid-state chemical reactions, as shown in Figure 7(a).^77^

They determined the shape of this step function by utilizing a transient 3D computational fluid dynamics model of the reactor inlet under different operational conditions and by gas-injection tracer studies. Subsequent chemical reactions are viewed as a black box, characterized by kinetic models from solid-state kinetic theory, as shown in Figure 7(b). These models monitor a single variable, α, symbolizing the reaction extent, adhering to the Arrhenius behavior according to the following equation:^78^
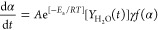
16where *Y*_H_2_O_ represents the mole fraction of steam in the inlet, and γ is an exponent determining the reliance on steam concentration. The example given in Figure 7(b) depicts a second order relation represented by the formula *f*(α) = (1 – α)^2^. The time duration of the transient pulse-like H_2_ input into the continuously stirred tank reactors (CSTR) is extended as a result of dispersion and mixing processes (shown in Figure 7(c)). Using the least-squares method, the kinetic model and its associated parameters are determined. When a single kinetic model does not align well with the experimental data, two parallel kinetic models are employed. This method enables the simultaneous calculation of all kinetic parameters, considering the impact of physical processes dependent on the experimental setup. However, it necessitates numerical reactor simulation and data-fitting calculations, offering a suitable approach to precisely investigate the oxidation kinetics.^78,79^

**Figure 8:**
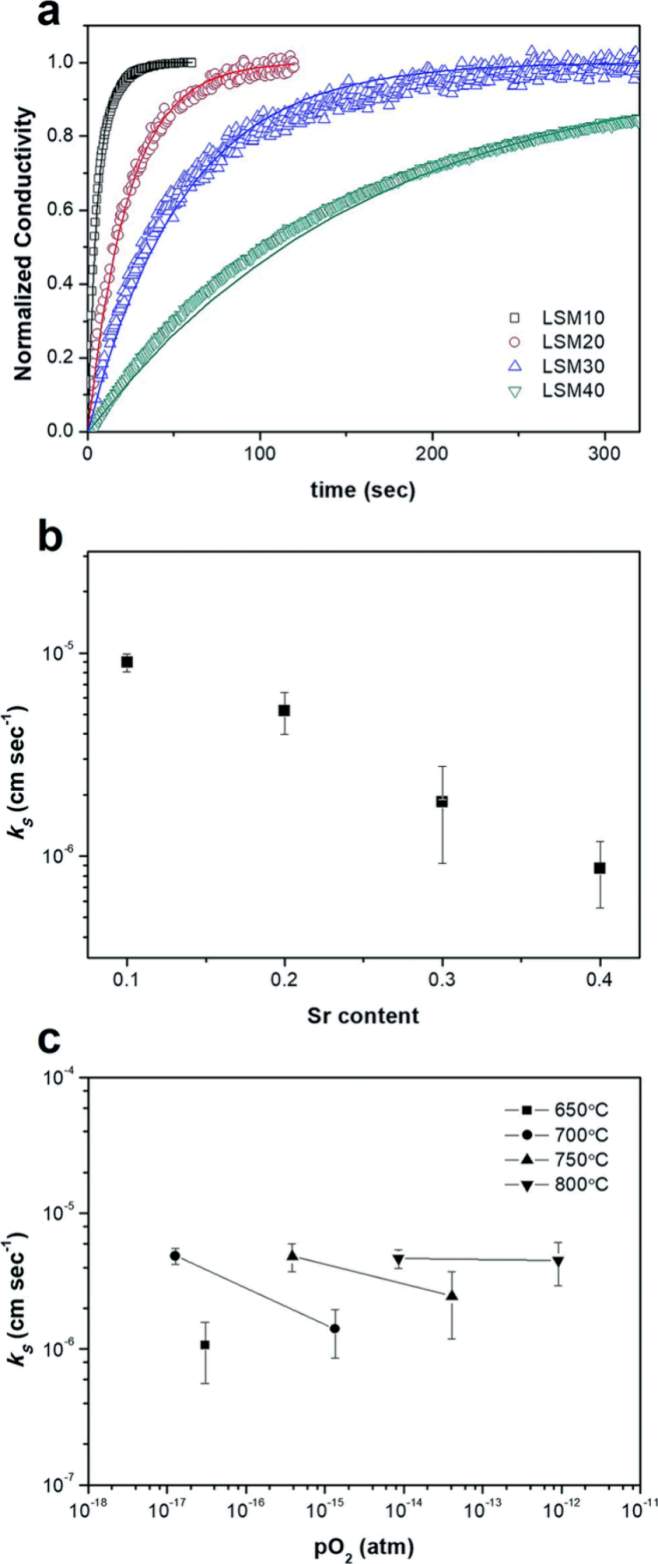
(a) Relaxation profiles of La_1–*x*_Sr_*x*_MnO_3−δ_ with different Sr contents at 800 °C, *P*_O_2__ = 9.0 × 10^–15^ atm, (b) a comparative analysis of the *K*_s_ values under same conditions, and (c) surface oxygen exchange coefficients of La_0.8_Sr_0.2_MnO_3−δ_ across different temperature and *P*_O_2__ values within a CO/CO_2_ environment. The solid lines in (a) depict the fitted profiles.^80^ Reproduced with permission from ref (80). Copyright 2018 RSC Publishing.

The electrical conductivity relaxation (ECR) method, with its long history dating back to its earliest use by Dünwald and Wagner in 1934, is considered a “classic” tool in some sense. WooChul Jung’s group measured the surface oxygen exchange kinetic parameters of (La,Sr)MnO_3−δ_ thin film using electrical conductivity relaxation (ECR) measurements. Two electrodes (Pt) were placed on the La_1–*x*_Sr_*x*_MnO_3−δ_ coating.^80^ In order to support the hypothesis of a first-order surface oxygen exchange reaction and the linear variation of conductivity with oxygen partial pressure, experiments were conducted for both the reduction and oxidation process. The experimental data were fitted by the following equation:^81^
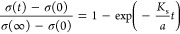
17where σ symbolizes the conductivity with respect to time, *K*_s_ stands for the surface reaction rate constant, and *a* is the sample thickness. The solid-state reaction rate constant *K*_s_ can be obtained from both experimental data and eq 17. The ECR method permits the investigation of surface oxygen exchange only and not the bulk. For example, YeonJu Kim et al. investigated the influence of the Sr concentration on the surface reaction kinetics of La_1–*x*_Sr_*x*_MnO_3−δ_ as shown in Figure 8 (a). Thin films of La_1–*x*_Sr_*x*_MnO_3−δ_ (where *x* = 0.1, 0.2, 0.3, 0.4) are synthesized through pulsed laser deposition, resulting in flat and dense surfaces. These films are subsequently analyzed for their *K*_s_ utilizing ECR under operational conditions (with temperatures ranging from 650 to 800 °C and oxygen partial pressures spanning from 2.9 × 10^–19^ to 9.0 × 10^–13^ atm). The Sr concentration increases, leading to a significant deceleration in oxygen exchange, as depicted in Figure 8 (b). Figure 8 (c) shows that for a given Sr concentration the oxygen exchange displays limited fluctuations across a broad range of *P*_O_2__ values near the 800 °C target temperature. These findings have practical implications for selecting an optimal oxygen carrier composition for enhanced fuel production performance.^80^

## 5 Development of Perovskite Oxides for Solar Thermochemical Water-Splitting

As previously mentioned, the two-step STCH cycle capitalizes on the redox behavior of metal oxides, allowing for the water-splitting reaction to occur at lower temperatures and directly controlling the amount of H_2_ produced by the quantity of oxide used.^82−85^ In this case, searching for a suitable redox material with satisfactory properties is the key research area. Theoretically, a metal oxide used in water-splitting should consider the following:

- Large reducibility (Δδ) of the materials at moderate temperatures.
- Suitable thermodynamic properties: materials suitable for H_2_O splitting require specific thermodynamic properties (reduction of enthalpy; reduction of entropy) within an appropriate window of values.^86^
- Fast redox kinetics.
- Morphological
- Phase stability.
- Synthesis method used to synthesize the STCH material.
- Cycling capability of the materials across the temperature range.

Most of the studies focus on material evaluation for STCH. Tables 1–10 collect and summarize the following performance metrics, consolidating units to a single format:

- Temperature range, which consists of the reduction temperature and the oxidation temperature.
- The amount of time the sample was exposed to the reduction temperature and oxidation temperature.
- The concentration of steam (H_2_O, % vol) used for the reoxidation of the material.
- The gas used to reduce the material and carry the steam.
- The amount of O_2_ released by the material at the end of the reduction time.

**Table 1:** STCH Performance of Mn-Based Perovskite Oxides under Inert Gas Conditions

composition	synthesis method	cycles	Δδ	temp. red./ox. (°C)	time red./ox. (min)	H_2_O (% vol)	gas red./ox.	O_2_ yield (μmol g^–1^)	H_2_ yield (μmol g^–1^)	average H_2_ production rate (μmol g^–1^ min^–1^)	ref
LaMnO_3−δ_	Pechini	3		1350/1000	1/60	84.0	N_2_/N_2_	112.8^a^	68.1^a^	1.14	(90)
La_0.9_Sr_0.1_MnO_3−δ_	solid-state	1	0.014	1400/800	12/8	20.0	Ar/Ar	24.6^a^	40.6^a^	5.08	(91)
La_0.7_Sr_0.3_MnO_3−δ_	solid-state	1	0.072	1400/800	60/31	20.0	Ar/Ar	151.8^a^	253.6^a^	8.18	(91)
La_0.6_Sr_0.4_MnO_3−δ_	solid-state	8	0.127	1400/800	70/65	20.0	Ar/Ar	218.8^a^	397.8^a^	6.12	(91)
La_0.8_Sr_0.2_MnO_3−δ_	solid-state	21	0.017	1400/800	47/16	20.0	Ar/Ar	64.7^a^	129.0^a^	8.06	(91)
La_0.8_Sr_0.2_MnO_3−δ_	commercial	1		1400/-	45/-		Ar/-	112.0			(95)
La_0.65_Sr_0.35_MnO_3−δ_	commercial	1		1400/1050	45/45	80.0	Ar/Ar	166.0	124.0	2.76	(95)
La_0.65_Sr_0.35_MnO_3−δ_	commercial	2		1400/1050		80.0	Ar/Ar		113.0		(95)
La_0.5_Sr_0.5_MnO_3−δ_	solid-state	1		1400/1000	45/45	80.0	Ar/Ar	298.0	195.0	4.33	(95)
La_0.5_Sr_0.5_MnO_3−δ_	solid-state	2		1400/1000		80.0	Ar/Ar		160		(95)
La_0.5_Sr_0.5_MnO_3−δ_	solid-state	1		1400/900	45/45	80.0	Ar/Ar	298.0	170.0	3.78	(95)
La_0.5_Sr_0.5_MnO_3−δ_	solid-state	2		1400/900			Ar/Ar		133		(95)
Sr_0.75_Ce_0.25_MnO_3−δ_	Pechini	3		1400/1000	5.5/20	40.0	Ar/Ar	104.0	224.0	11.2	(76)
Sr_0.75_Ce_0.25_MnO_3−δ_	Pechini	3		1350/850	5.5/20	40.0	Ar/Ar	68.0	98.0	4.9	(76)
La_0.65_Sr_0.35_MnO_3−δ_	commercial	1		1400/-	45/-		Ar/-	100.0			(102)
La_0.5_Sr_0.5_MnO_3−δ_	solid-state	1		1400/1000	100/100		N_2_/N_2_	193.0	308.0	3.08	(102)
La_0.65_Ca_0.35_MnO_3−δ_	solid-state	1		1400/1100	45/-		Ar/-	109.0			(102)
La_0.5_Ca_0.5_MnO_3−δ_	solid-state	1		1400/1000	100/100		N_2_/N_2_	272.0	407.0	4.07	(102)
La_0.35_Ca_0.65_MnO_3−δ_	solid-state	1		1400/1100	45/-		Ar/-	653.0			(102)
La_0.5_Sr_0.5_MnO_3−δ_	solid-state	1	0.088	1400/1100	45/-		Ar/-	198.0			(103)
Nd_0.5_Sr_0.5_MnO_3−δ_	solid-state	1	0.117	1400/1100	45/-		Ar/-	264.0			(103)
Sm_0.5_Sr_0.5_MnO_3−δ_	solid-state	1	0.135	1400/1100	45/-		Ar/-	301.0			(103)
Gd_0.5_Sr_0.5_MnO_3−δ_	solid-state	1	0.148	1400/1100	45/-		Ar/-	330.0			(103)
Dy_0.5_Sr_0.5_MnO_3−δ_	solid-state	1	0.179	1400/1100	45/-		Ar/-	393.0			(103)
Y_0.5_Sr_0.5_MnO_3−δ_	solid-state	1	0.192	1400/1100	45/140		N_2_/N_2_	481.0	320.0	2.29	(103)
La_0.5_Ca_0.5_MnO_3−δ_	solid-state	1	0.122	1400/1100	45/-		Ar/-	312.0			(103)
Nd_0.5_Ca_0.5_MnO_3−δ_	solid-state	1	0.135	1400/1100	45/-		Ar/-	343.0			(103)
Sm_0.5_Ca_0.5_MnO_3−δ_	solid-state	1	0.147	1400/1100	45/-		Ar/-	361.0			(103)
Gd_0.5_Ca_0.5_MnO_3−δ_	solid-state	1	0.134	1400/1100	45/-		Ar/-	404.0			(103)
Dy_0.5_Ca_0.5_MnO_3−δ_	solid-state	1	0.200	1400/1100	45/-		Ar/-	493.0			(103)
Y_0.5_Ca_0.5_MnO_3−δ_	solid-state	1	0.200	1400/1100	45/140		N_2_/N_2_	593.0	310.0	2.21	(103)
La_0.8_Sr_0.2_MnO_3−δ_	commercial	4	0.013	1400/1400			N_2_/N_2_	145.2	49.6		(97)
La_0.65_Sr_0.35_MnO_3−δ_	commercial	4	0.055	1400/1400			N_2_/N_2_	137.9	49.6		(97)
La_0.8_Sr_0.2_MnO_3−δ_	CALPHAD			1400/800		20.0		58.0^a^	116.1^a^		(98)
La_0.7_Sr_0.3_MnO_3−δ_	CALPHAD			1400/800		20.0		126.0^a^	250.5^a^		(98)
La_0.6_Sr_0.4_MnO_3−δ_	CALPHAD			1400/800		20.0		116.1^a^	392.9^a^		(98)
LaMnO_3−δ_	Pechini	1		1300/900	60/60	40.0	Ar/Ar	76.0	48.0	0.80	(110)
La_0.6_Sr_0.4_MnO_3−δ_	Pechini	1		1300/900	60/60	40.0	Ar/Ar	126.0	215.0	3.58	(110)
La_0.6_Ca_0.4_MnO_3−δ_	Pechini	1		1300/900	60/60	40.0	Ar/Ar	167.0	312.0	5.2	(110)
Ba_0.4_La_0.6_MnO_3−δ_	Pechini	1		1300/900	60/60	40.0	Ar/Ar	113.0	179.0	2.98	(110)
La_0.9_Sr_0.1_MnO_3−δ_		1	0.010	1385/840	40/5	20.0	Ar/Ar	TBD	TBD	0	(111)
La_0.8_Sr_0.2_MnO_3−δ_		1	0.040	1440/795	40/15	20.0	Ar/Ar	TBD	TBD	0	(111)
La_0.7_Sr_0.3_MnO_3−δ_		1	0.070	1440/810	40/30	20.0	Ar/Ar	TBD	TBD	0	(111)
La_0.6_Sr_0.4_MnO_3−δ_		1	0.130	1385/840	40/30	20.0	Ar/Ar	TBD	TBD	0	(111)
La_0.6_Sr_0.4_MnO_3−δ_	Pechini	1		1300/900	60/60	40.0	Ar/Ar	126.0	234.0	3.90	(104)
La_0.8_Ca_0.2_MnO_3−δ_	Pechini	1		1300/900	60/60	40.0	Ar/Ar	94.0	187.0	3.12	(104)
La_0.6_Ca_0.4_MnO_3−δ_	Pechini	1		1300/700	60/60	40.0	Ar/Ar	167.0	194.5	3.24	(104)
La_0.6_Ca_0.4_MnO_3−δ_	Pechini	1		1300/800	60/60	40.0	Ar/Ar	167.0	235.5	3.93	(104)
La_0.6_Ca_0.4_MnO_3−δ_	Pechini	6		1300/900	60/60	40.0	Ar/Ar	167.0	314.0	5.23	(104)
La_0.6_Ca_0.4_MnO_3−δ_	Pechini	1		1300/1000	60/60	40.0	Ar/Ar	167.0	267.5	4.46	(104)
La_0.6_Ca_0.4_MnO_3−δ_	Pechini	1		1300/1100	60/60	40.0	Ar/Ar	167.0	241.9	4.03	(104)
La_0.4_Ca_0.6_MnO_3−δ_	Pechini	1		1300/900	60/60	40.0	Ar/Ar	224.0	243.0	4.05	(104)
La_0.2_Ca_0.8_MnO_3−δ_	Pechini	1		1300/900	60/60	40.0	Ar/Ar	402.0	162.0	2.70	(104)
La_0.6_Sr_0.4_MnO_3−δ_	Pechini	1	0.058	1300/900	60/60	40.0	Ar/Ar	131.0	234.0	3.90	(105)
Ce_0.1_Sr_1.9_MnO_3−δ_	evaporation	2	0.180	1400/1000	5.5/20	40.0	Ar/Ar	240.0	218.0	10.9	(108)
Ce_0.2_Sr_1.8_MnO_3−δ_	evaporation	2		1400/1000	5.5/20	40.0	Ar/Ar	168.0	247.0	12.35	(108)
Ce_0.3_Sr_1.7_MnO_3−δ_	evaporation	2	0.090	1400/1000	5.5/20	40.0	Ar/Ar	129.0	166.0	8.30	(108)
SrMnO_3_	Pechini	1		1400/1000	5.5/16.7	50.0	Ar/Ar		0.0	0	(108)
La_0.6_Sr_0.4_MnO_3−δ_	Pechini		0.0038	1300/700	13.3/20	1.3	Ar/Ar		17.0	0.85	(101)
La_0.6_Sr_0.4_MnO_3−δ_	Pechini		0.0059	1300/500	13.3/20	1.3	Ar/Ar		27.0	1.35	(101)
La_0.6_Sr_0.4_MnO_3−δ_	Pechini		0.0121	1300/500	13.3/40	1.3	Ar/Ar		55.0	1.38	(101)
Sr_0.86_Ce_0.14_MnO_3−δ_	Pechini	1		1350/850	5.5/20	40.0	Ar/Ar		91.9	4.60	(109)
Sr_0.86_Ce_0.14_MnO_3−δ_	Pechini	1		1350/1000	5.5/20	40.0	Ar/Ar		177.1	8.86	(109)
Sr_0.86_Ce_0.14_MnO_3−δ_	Pechini	1		1400/850	5.5/20	40.0	Ar/Ar		153.2	7.66	(109)
Sr_0.86_Ce_0.14_MnO_3−δ_	Pechini	1		1400/1000	5.5/20	40.0	Ar/Ar		242.4	12.12	(109)
Sr_0.8_Ce_0.2_MnO_3−δ_	Pechini	1		1350/850	5.5/20	40.0	Ar/Ar		99.9	5.00	(109)
Sr_0.8_Ce_0.2_MnO_3−δ_	Pechini	1		1350/1000	5.5/20	40.0	Ar/Ar		154.5	7.73	(109)
Sr_0.8_Ce_0.2_MnO_3−δ_	Pechini	1		1400/850	5.5/20	40.0	Ar/Ar		158.5	7.93	(109)
Sr_0.8_Ce_0.2_MnO_3−δ_	Pechini	1		1400/1000	5.5/20	40.0	Ar/Ar		220.0	11.00	(109)
Sr_0.7_Ce_0.3_MnO_3−δ_	Pechini	1		1350/850	5.5/20	40.0	Ar/Ar		90.6	4.53	(109)
Sr_0.7_Ce_0.3_MnO_3−δ_	Pechini	1		1350/1000	5.5/20	40.0	Ar/Ar		163.8	8.19	(109)
Sr_0.7_Ce_0.3_MnO_3−δ_	Pechini	1		1400/850	5.5/20	40.0	Ar/Ar		210.4	10.52	(109)
Sr_0.7_Ce_0.3_MnO_3−δ_	Pechini	1		1400/1000	5.5/20	40.0	Ar/Ar		305.0	15.25	(109)
La_0.6_Sr_0.4_MnO_3_		1	0.0322	1500/800	20.5/4.3	50.0	Ar/Ar		147.32^#^	34.26	(88)
La_0.8_Sr_0.2_MnO_3_		1	0.0319	1500/800	14.7/5.7	50.0	Ar/Ar		138.39^#^	24.28	(88)
La_0.6_Sr_0.4_MnO_3_		1	0.0179	1400/800	31.0/5.4	50.0	Ar/Ar		80.36^#^	14.88	(88)
La_0.8_Sr_0.2_MnO_3_		1	0.015	1400/802	14.8/4.9	50.0	Ar/Ar		62.5^#^	12.76	(88)

a Value converted from mL g_material_^–1^. Standard volume occupied: 22400 mL mol^–1^.

Moreover, the average H_2_ production rates are also included (Tables 1–10), providing a more comprehensive view of the data and allowing for better comparisons.

- **Thermodynamic and kinetic influences**: The average H_2_ production rate is influenced by both the thermodynamic and kinetic properties of perovskite oxides. As shown in the tables, a higher Δδ indicates stronger thermodynamic properties, which are critical for the first step of the water-splitting process.^87^ However, the second step of water-splitting is primarily dependent on the kinetic properties of the perovskite material. This analysis indicates that both kinetics and thermodynamics are crucial parameters for evaluating the performance of perovskites in solar thermochemical water-splitting.^88^
- **Comparison with fluorite oxides**: In comparison, fluorite oxides, particularly CeO_2_, outperform perovskites in terms of H_2_ production rates.^26^ This is primarily due to their higher surface reactivity and faster oxygen exchange kinetics. Additionally, fluorite oxides exhibit better phase stability and resistance to sintering at high temperatures, ensuring stable performance over long-term cycling.^89^

### 5.1 Mn-Based Perovskite Oxides as Redox-Active Oxygen Exchange Materials

**Figure 9:**
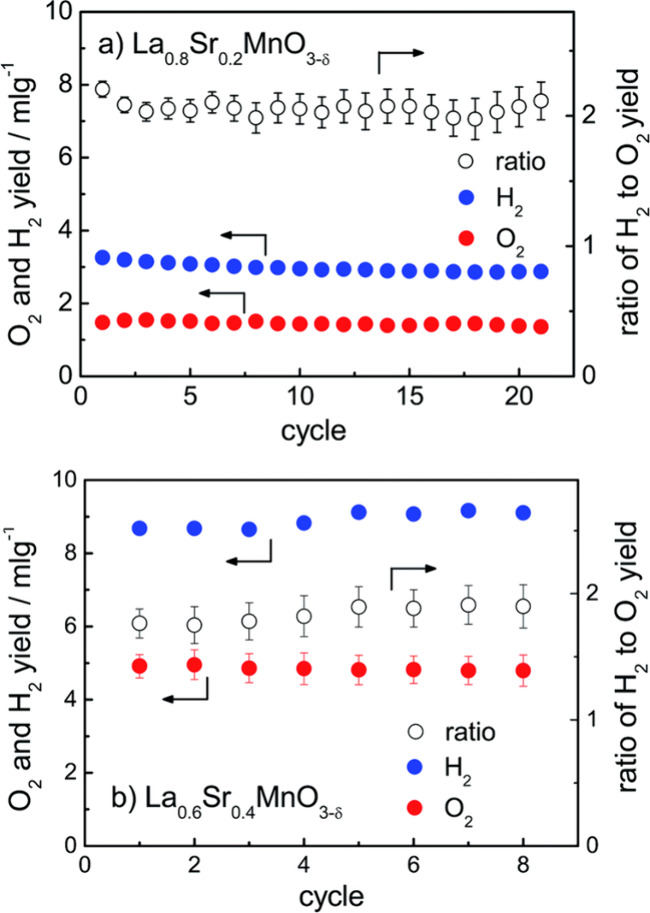
H_2_ and O_2_ production in La_0.8_Sr_0.2_MnO_3−δ_ and La_0.6_Sr_0.4_MnO_3−δ_, respectively. Reduction times for La_0.8_Sr_0.2_MnO_3−δ_ and La_0.6_Sr_0.4_MnO_3−δ_ were 47 and 70 min, and oxidation times were 16 and 65 min, respectively. The uncertainty is derived from calibration signal drift. Where omitted, error bars fall within the data symbol.^91^ Reproduced with permission from ref (91). Copyright 2014 RSC Publishing.

Lanthanum manganite (LaMnO_3−δ_) in near-stoichiometry contains an average +3 oxidation state, accompanied by Jahn–Teller distortion. Researchers like Nobuyuki Gokon et al. have observed that LaMnO_3_ displays high oxygen-release activity, although the oxygen release rate decreased with cycling.^90^ The introduction of Sr^2+^ can increase the reduction extent (Δδ) and improve fuel production compared to LaMnO_3−δ_.^91,92^ However, the ideal Sr content for substituting La lies between 0.3 and 0, as exceeding this range negatively impacts the reoxidation yield.^93^ Aldo Steinfeld’s group conducted an experimental and thermodynamic investigation of La_1–*x*_Sr_*x*_MnO_3−δ_ (*x* = 0.3, 0.4). They developed a defect model based on low-temperature oxygen nonstoichiometry data and extrapolated it to higher temperatures. Theoretical solar-to-fuel conversion efficiencies for La_0.6_Sr_0.4_MnO_3_ are 16% at 1800 K and 13% at 1600 K.^94^ Recently, Jiahui Lou et al. introduced a new model for rapidly evaluating nonstoichiometric oxides used in two-step solar thermochemical cycling, focusing on solar-to-fuel efficiency. It considers the oxide material thermodynamics and typical experimental conditions. The model’s application revealed that CeO_2_, under optimal cycling conditions, achieved the highest efficiency (12.9% at a reduction temperature of 1500 °C) compared to other materials, including 20 mol % Zr-doped CeO_2_ (10.1%), La_0.8_Sr_0.2_MnO_3_ (3.3%), and La_0.6_Sr_0.4_MnO_3_ (2.5%). Moreover, the model could be enhanced by considering reaction kinetics for oxidation temperatures under 800–1000 °C.^88^ The mixed ionic-electronic conductor La_1–*x*_Sr_*x*_MnO_3−δ_ (*x* = 0.2, 0.35, 0.5) is commonly used in fuel cells due to its reactivity toward oxygen vacancy formation upon heating and its ability to transport oxide ions, which is also vital during the second step of STCH. Moreover, Stéphane Abanades’s group highlighted the importance of significant ionic conductivity at high temperatures for materials used in water-splitting, which is beneficial for bulk diffusion processes involved during the reduction and oxidation steps. Additionally, the oxidation temperature must be sufficiently high to promote oxide ionic bulk diffusion, a key kinetic limitation of perovskite oxides. They investigated the capabilities of La_*x*_Sr_1–*x*_MnO_3_ (*x* = 0.8, 0.65, and 0.5) for the STCH reaction. Upon the substitution of Sr^2+^ for La^3+^, an increase in the extent of reduction was observed due to charge imbalances, resulting in a higher nominal oxidation state on the B-site of the perovskite. This led to oxygen production yields of 112, 166, and 298 μmol g^–1^ for Sr^2+^ substitutions of 20%, 35%, and 50%, respectively. Furthermore, in the observed mixed Mn^3+^/Mn^4+^ valence state, the higher the Mn^4+^ content, the greater the favorability for the reduction of the La_1–*x*_Sr_*x*_MnO_3−δ_. H_2_ production yields of 124 and 195 μmol g^–1^ were observed for La_0.65_Sr_0.35_MnO_3−δ_ and La_0.5_Sr_0.5_MnO_3−δ_, respectively. These yields were attributed mainly to the strong capability of the perovskite structure to accommodate large oxygen vacancies upon reduction, allowing for considerable H_2_ production. Ultimately, the thermal reduction and water-splitting capabilities of perovskites lie in the redox chemistry of the ABO_3_ structure and in the B–O bond properties, such as the B atomic ionization potential, the B–O bond energy, or the orbital overlap between O 2p and B 3d states.^95^ Similarly, Yang et al. studied the thermodynamics and kinetics of La_1–*x*_Sr_*x*_MnO_3−δ_ (*x* = 0.1, 0.2, 0.3, and 0.4). The authors noted several important factors: (1) Grain size decreased with increasing Sr^2+^ content, while porosity remained constant. (2) Oxygen release and hydrogen production exhibited monotonic increase with Sr^2+^ content, consistent with thermodynamic expectations. Nonetheless, there was an associated increase in the steam required for fuel production. (3) The kinetics of fuel production were significantly affected by the Sr^2+^ content. They also discovered that higher levels of strontium (Sr) doping led to reduced rates of steam-to-hydrogen conversion. Furthermore, with an increase in the Sr content, the rate of fuel production decreased, suggesting that intermediate compositions could provide the most favorable combination of properties. La_0.8_Sr_0.2_MnO_3−δ_ and La_0.6_Sr_0.4_MnO_3−δ_ exhibited consistent hydrogen and oxygen evolution over 21 and 8 consecutive test cycles, respectively. The former released an average of 64.7 μmol g^–1^ O_2_ and produced 129.0 μmol g^–1^ H_2_, while the latter released an average of 218.8 μmol g^–1^ O_2_ and produced 397.3 μmol g^–1^ H_2_, as shown in Figure 9.^91^

In addition to the study of La_0.6_Sr_0.4_MnO_3_ presented earlier, Luciani et al. also investigated La_0.6_Sr_0.4_MnO_3_, which exhibited a tetragonal structure for STCH applications. Treatment of La_0.6_Sr_0.4_MnO_3_ in a H_2_ stream results in the coexistence of tetragonal and hexagonal phases, indicating that reduction in a hydrogen atmosphere induces a phase transition in the perovskite structure.^96^ Later, Mara Orfila et al. investigated the STCH performance of two commercial perovskites with structure ABO_3_: La_0.8_Sr_0.2_MnO_3−δ_ (LSM-20) and La_0.65_Sr_0.35_MnO_3−δ_ (LSM-35). They reveal a slight dependence between the reduction temperature and the strontium quantity within the structure. Higher strontium contents (as found in LSM-35) result in lower reduction temperatures compared to LSM-20, which has a lower strontium content. This result can be attributed to the higher quantity of strontium in the perovskite, which leads to the formation of more oxygen vacancies to maintain electroneutrality. This increase in the number of oxygen vacancies improves the oxygen mobility within the perovskite network, thereby facilitating the reduction process. H_2_ production also increases with the doping level of Sr.^91,94,97^ Alexander H. Bork et al. used data from Yang et al. as input to evaluate La_1–*x*_Sr_*x*_MnO_3−δ_ using the calculation of phase diagrams (CALPHAD) method. This method enables the description of multicomponent systems based on a physicochemical principle. In contrast to previous thermodynamic evaluations of perovskites, which relied on a single literature source and were constrained to a limited temperature range, the CALPHAD approach incorporates all relevant data available in the literature. Moreover, the CALPHAD results obtained are comparable to those obtained by Chih-Kai Yang et al. for H_2_ production, with results of 116.1 and 392.9 μmol g^–1^ for La_0.8_Sr_0.2_MnO_3−δ_ and La_0.6_Sr_0.4_MnO_3−δ_, respectively.^98^

Additionally, Gager et al. discovered that porous (La_0.65_Sr_0.35_)_0.95_MnO_3−δ_ showed great promise as a catalyst for the STCH reaction. The use of porous ceramic foams offered several advantages, including a high specific area, significant porosity, and controlled permeability. The replica foams were carefully designed to have around 85% overall porosity, with about 65% of that being open porosity. Moreover, the foams retained their structure throughout 50 isothermal cycles at 1400 °C, while consistently yielding approximately 200 μmol g^–1^ of H_2_.^99^ In the field of STCH, besides delving into the impacts of doping, Lee et al. researched the effects of *P*_H_2_O_/*P*_H_2__ under isothermal cycling for La_0.65_Sr_0.35_MnO_3−δ_ and La_0.6_Sr_0.4_MnO_3−δ_. With *P*_O_2__ changes induced by changing *P*_H_2_O_ relative to *P*_H_2__, a general trend was observed for reduction extent, Δδ, and H_2_ production with increasing temperature and *P*_O_2__, with La_0.65_Sr_0.35_MnO_3−δ_ producing 306.0 g mol^–1^ and La_0.6_Sr_0.4_MnO_3−δ_ producing 355.5 g mol^–1^ at 1400 °C.^91,100^

Later, Jonathan R. Scheffe’s group conducted a study to investigate the morphological stability of La_0.6_Sr_0.4_MnO_3−δ_ during redox cycling using an infrared laser-based heating device. This device was employed to simulate the heat flux conditions expected in large-scale solar simulators. The study focused on these two porous materials and observed a correlation between the reaction extent and rates up to cycle 120, which was attributed to sintering and structural changes. In terms of water-splitting, the researchers found that ceria outperformed LSM40 due to LSM40’s lower effectiveness at low *P*_O_2__ during the oxidation process. Moreover, the substitution of Sr^2+^ into the LaMnO_3−δ_ perovskite causes rhombohedral distortions in space group *R*3̅*c*, generating a high concentration of Mn^4+^ due to the charge balance change induced by the replacement of La^3+^ with Sr^2+^. Furthermore, a concentration of Sr^2+^ ≥ 47% leads to a tetragonal structure (*I*4/*mcm*), and a concentration of Sr^2+^ > 70% leads to a cubic structure (Pm3m).^101^

The effect of Ca^2+^ doping into the lanthanum-manganite perovskite La_1–*x*_Ca_*x*_MnO_3−δ_ was also investigated. Sunita Dey et al. researched the impact of varying the Ca^2+^ content on LaMnO_3−δ_. They observed an increase in O_2_ release with an increase in Ca^2+^ doping. Notably, La_0.5_Ca_0.5_MnO_3−δ_ produced 316.0 μmol g^–1^ of O_2_, surpassing the production of the O_2_ La_0.5_Sr_0.5_MnO_3−δ_ (201 μmol g^–1^) and CeO_2_ (63 μmol g^–1^). Similarly, La_0.35_Ca_0.65_MnO_3−δ_ exhibited even higher O_2_ production 653.0 μmol g^–1^. During reoxidation, La_0.5_Sr_0.5_MnO_3−δ_ produced 308.0 μmol g^–1^ of H_2_, while La_0.5_Ca_0.5_MnO_3−δ_ produced 407.0 μmol g^–1^ of H_2_. This difference was attributed to the orthorhombic structure of Ca^2+^-doped LaMnO_3−δ_, which exhibited superior activity compared to the rhombohedral structure of its Sr^2+^-doped counterpart due to the former’s distortion in the octahedral MnO_6_. Additionally, the kinetics become sluggish with a high Ca^2+^ content.^102^ A few months later, Dey et al. demonstrated the remarkable relationship between distortion and size disorder in relation to the H_2_ yield of Ln_0.5_A_0.5_MnO_3_ (Ln = Lanthanide, A = Ca^2+^, Sr^2+^). Accordingly, the O_2_ released during the reduction step and the H_2_ produced during the water-splitting step depend on the size of the lanthanide ion present at the A-site. As the size of the rare-earth ion decreases, the yields of O_2_ and H_2_ increase, with the onset temperature of oxygen evolution shifting to lower temperatures. This effect is attributed to small rare-earth ions, such as yttrium, having the highest lattice distortion and the smallest tolerance factor, indicating an increase in the tilting of the MnO_6_ octahedra, which promotes the release of oxide ions. Specifically, a decrease in the tolerance factor leads to a significant reduction in the Mn–O–Mn bond angles, diminishing the spatial overlap between the Mn e_g_ and O 2pσ orbitals. The mismatch between the radii of the lanthanide ion and the alkaline-earth ion causes high site disorder where the oxygen is greatly displaced from the mean position, acting as the driving force for oxygen evolution. Thus, Y_0.5_Sr_0.5_MnO_3−δ_ and Y_0.5_Ca_0.5_MnO_3−δ_ released the highest amount of O_2_, with 481.0 and 593.0 μmol g^–1^, respectively. While Y_0.5_Ca_0.5_MnO_3−δ_ released the highest amount of O_2_, Y_0.5_Sr_0.5_MnO_3−δ_ produced the highest amount of H_2_ with 320.0 μmol g^–1^ versus 310.0 μmol g^–1^.^103^

Lulu Wang et al. also investigated the effect of different Ca dopant levels on LaMnO_3−δ_. With increasing Ca^2+^ dopant levels, the temperature required to kick-start the evolution of the O_2_ significantly decreased and the rate of release of the O_2_ increased rapidly. Nevertheless, the trend for H_2_ production performance was not in full accordance with the O_2_ evolution trend, improving with Ca^2+^ dopant level from 0.2 to 0.4 and then decreasing thereafter. La_0.6_Ca_0.4_MnO_3_ displays the highest hydrogen production (314 μmol g^–1^) at the red./ox. temperature sof 1300 °C/900 °C and exhibits superior stability with stable hydrogen and oxygen production.^104^ Subsequently, their research group elucidated a linear relationship between H_2_ generation and the extent of oxygen vacancy (δ) in La_0.6_Sr_0.4_BO_3_ (B = Cr, Mn, Fe, Co, and Ni).^105^ Christopher L. Muhich et al. performed a thermodynamic analysis of six candidates. According to the solar-to-fuel conversion efficiency from high to low, the order follows Zr-doped CeO_2_ > undoped CeO_2_ > La_0.6_Ca_0.4_MnO_3_ > La_0.6_Ca_0.4_Mn_0.6_Al_0.4_O_3_ > La_0.6_Sr_0.4_MnO_3_ > La_0.6_Sr_0.4_Mn_0.6_Al_0.4_O_3_. This ranking is attributed to their relative reoxidizability and reducibility.^106^

Teruki Motohashi et al. investigated the effects of lanthanoid (Ln = La, Nd, Gd, Y) substitution on BaLnMn_2_O_5+δ_, an A-site-ordered double perovskite. Interestingly, they observed that the water-splitting reaction occurs only once the oxygen content in the sample is less than oxygenated O_5.5_. This points to the exclusive activity of Mn^2+^, while Mn^3+^ and Mn^4+^ remain inactive. Additionally, the reoxygenation rate from O_5.0_ to O_5.5_ is influenced by an increase in coordination number at the Ln^3+^ site. Larger Ln members (e.g., La^3+^) exhibit greater stability in the deoxygenated O_5.0_ form, resulting in a higher endothermic oxygenation enthalpy and stronger reductive reactivity. Consequently, BaLaMn_2_O_5+δ_ exhibited the highest H_2_ production (1093.2 μmol g^–1^) and the shortest reduction time. As the Ln^3+^ size decreased, H_2_ production decreased and reduction time increased, culminating with BaYMn_2_O_5+δ_ showing almost undetectable H_2_ production (14.3 μmol g^–1^). These differences in reactivity might be explained by considering electrostatic interactions within the crystal lattice.^107^

Débora R. Barcellos et al. discovered a Ruddlesden–Popper phase layered perovskite, Ce_*x*_Sr_2–*x*_MnO_4_, and its applicability for STCH through DTF calculations. Accordingly, the suitability of an oxide for STCH is tied to the energy required to create and fill oxygen vacancies during the STCH cycling. The oxygen vacancy formation energy, *E*_v_, directly impacts the driving force for oxide reoxidation in the presence of steam and must be low enough to allow a feasible level of nonstoichiometry during reduction, yet high enough to overcome the O–H bond strength of water during oxidation. Through DFT calculations, the oxygen vacancy formation energy of Ce_*x*_Sr_2–*x*_MnO_4_ was computed to range from 1.8 to 2.7 eV, implying its good suitability for water-splitting (1.5–5 eV). Coupled with the enhanced oxygen ion mobility characteristic of layered perovskites, Ce_*x*_Sr_2–*x*_MnO_4_ demonstrated complete redox reversibility, with Ce_0.1_Sr_1.9_MnO_4_ having an oxygen release of 240.0 μmol g^–1^, Ca_0.2_Sr_1.8_MnO_4_ having a release of 168.0 μmol g^–1^, and Ca_0.3_Sr_1.7_MnO_4_ having a release of 129.0 μmol g^–1^, equating to hydrogen productions of 218.0, 247.0, and 166.0 μmol g^–1^, respectively. Conversely, Ce_0.1_Sr_1.9_MnO_4_ had the lowest oxygen vacancy formation energy and thus had the largest oxygen evolution, while Ca_0.3_Sr_1.7_MnO_4_ had the highest oxygen vacancy formation energy but the lowest oxygen evolution. Nevertheless, Ca_0.2_Sr_1.8_MnO_4_ had the highest H_2_ production because it balanced a moderate extent of reduction with an adequate driving force by having an intermediate oxygen vacancy formation energy.^108^

Recently, Bergenson-Keller et al. compared the work of Débora R. Barcellos et al. on the Ruddlesden–Popper layered perovskite Ce_*x*_Sr_2–*x*_MnO_4_ with the perovskite Sr_1–*x*_Ce_*x*_MnO_3−δ_. SrMnO_3−δ_ presented several phase transitions due to the concentration of Ce^2+^ used, starting off as hexagonal (*P*6_3_/*mmc*), cubic (Pm3m) at 5 mol % Ce^2+^, tetragonal (*I*4/*mcm*) at 8 mol % Ce^2+^, and orthorhombic (*Imma*) at 35 mol % Ce^2+^. At elevated temperatures, samples with ≤30 mol % Ce^2+^ transitioned to the *Pm*3*m* phase, while samples with 40 mol % Ce^2+^ transitioned to *I*4/*mcm* and then to *Pm*3*m*. The cerium-doped strontium manganite perovskite and its Ruddlesden–Popper counterpart demonstrated opposite trends for H_2_ production with an increasing Ce^2+^ content. Sr_0.8_Ce_0.2_MnO_3−δ_ produced the lowest H_2_ yield at 220.0 μmol g^–1^, while Sr_0.7_Ce_0.3_MnO_3−δ_ produced the highest at 305.0 μmol g^–1^, yet Ce_0.2_Sr_1.8_MnO_3−δ_ produced the highest at 247.0 μmol g^–1^ and Ce_0.3_Sr_1.7_MnO_3−δ_ produced the lowest at 166.0 μmol g^–1^ for their respective families. While the Ruddlesden–Popper family was expected to outperform its perovskite counterpart based on thermal reduction screening, the results proved otherwise. This was attributed to possible variability within the morphology or secondary-phase distribution within each sample and/or viability in the testing procedure, since both families were tested a year apart from each other. Moreover, the authors stress that under a local steam-to-hydrogen ratio deviation from pure steam conditions of 10 000:1, the hydrogen production of Sr_1–*x*_Ce_*x*_MnO_3−δ_ would be suppressed from that of Ce_*x*_Sr_2–*x*_MnO_4_. Nevertheless, a direct correlation between structure, Ce^2+^ content, and H_2_ yield could not be discerned.^109^Table 1 summarizes the STCH performance of Mn-based perovskite oxides under inert gas conditions.

Table 2 summarizes the STCH performance of Mn-based perovskite oxides under reducing gas conditions.

**Table 2:** STCH Performance of Mn-Based Perovskite Oxides under Reducing Gas Conditions

composition	synthesis method	cycles	Δδ	temp. red./ox. (°C)	time red./ox. (min)	H_2_O (%vol)	gas red./ox.	H_2_ yield (μmol g^–1^)	average H_2_ production rate (μmol g^–1^ min^–1^)	ref
BaYMn_2_O_5+δ_	citrate/encapsulation	1	0.5	500/500	-/150	2.3	N_2_–H_2_/N_2_	14.3^a^		(107)
BaGdMn_2_O_5+δ_	citrate/encapsulation	1	0.5	500/500	-/17500	2.3	N_2_–H_2_/N_2_	646.7^a^		(107)
BaNdMn_2_O_5+δ_	citrate/encapsulation	1	0.5	500/500	-/2000	2.3	N_2_–H_2_/N_2_	1064.6^a^		(107)
BaLaMn_2_O_5+δ_	citrate/encapsulation	1	0.5	500/500	-/1500	2.3	N_2_–H_2_/N_2_	1093.2^a^	0.73	(107)
(La_0.65_Sr_0.35_)_0.95_MnO_3−δ_	commercial	50	0.05	1400/1400	30/10		Ar–H_2_/Ar	200.0	6.67	(99)
La_0.65_Sr_0.35_MnO_3−δ_	commercial	1	0.092	1300/1300		24.82	Ar–H_2_^b^/Ar–H_2_	147.5		(92)
La_0.65_Sr_0.35_MnO_3−δ_	commercial	1	0.115	1350/1350		24.82	Ar–H_2_^b^/Ar–H_2_	234.5		(92)
La_0.65_Sr_0.35_MnO_3−δ_	commercial	1	0.131	1400/1400		24.82	Ar–H_2_^b^/Ar–H_2_	306.0		(92)
La_0.6_Sr_0.4_MnO_3−δ_	Pechini	1	0.087	1300/1300		24.82	Ar–H_2_^b^/Ar–H_2_	156.0		(92)
La_0.6_Sr_0.4_MnO_3−δ_	Pechini	1	0.123	1350/1350		24.82	Ar–H_2_^b^/Ar–H_2_	273.0		(92)
La_0.6_Sr_0.4_MnO_3−δ_	Pechini	1	0.144	1400/1400		24.82	Ar–H_2_^b^/Ar–H_2_	355.5		(92)

a Value converted from mL g_material_^–1^. Standard volume occupied: 22400 mL mol^–1^).

b Steam injected during reduction step. 1300 °C ≈ 11.66 vol % H_2_O, 1350 °C ≈ 6.69 vol % H_2_O, 1400 °C ≈ 4.05 vol % H_2_O.

### 5.2 MnAl-Based Perovskite Oxides as Redox-Active Oxygen Exchange Materials

**Figure 10:**
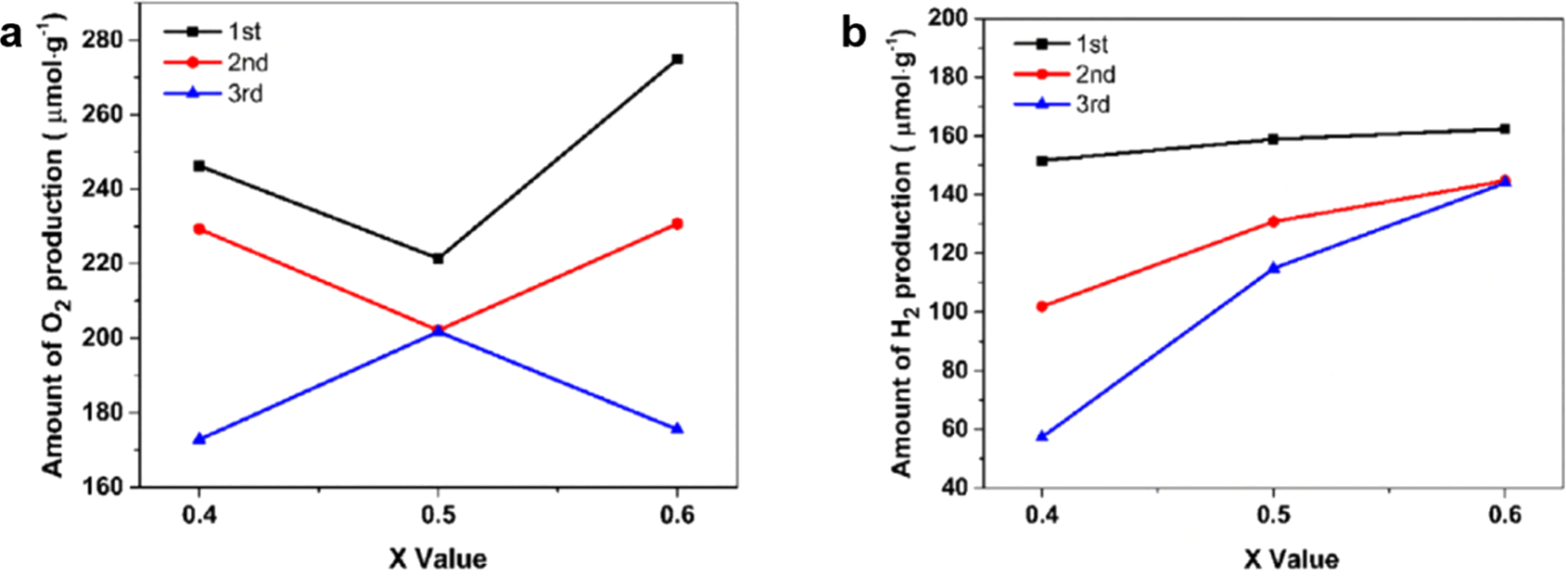
(a) The oxygen production and (b) the hydrogen production over three consecutive cycles for La_0.4_Sr_0.6_Mn_1–*x*_Al_*x*_ (*x* = 0.4, 0.5, and 0.6).^115^ Reproduced with permission from ref (115). Copyright 2022 Elsevier.

As mentioned previously, La–Mn perovskites often exhibit incomplete reoxidation. However, doping with aluminum (Al) at the B-site can enhance the efficiency of reoxidation and increase the extent of reduction.^112^ For example, McDaniel et al. investigated three perovskites (La_0.4_Sr_0.6_Mn_0.6_Al_0.4_O_3−δ_, La_0.6_Sr_0.4_Mn_0.6_Al_0.4_O_3−δ_, and La_0.4_Sr_0.6_Mn_0.4_Al_0.6_O_3−δ_) in the field of STCH and demonstrated that it is possible to achieve significant improvements in redox capacity and a dramatic increase in redox thermodynamics by tuning the combination of Mn and Al on the B-site of the perovskite structure. Lanthanum aluminate is known to remain stable in both oxidizing and reducing environments due to its cations maintaining a consistent oxidation state. McDaniel et al. introduced redox activity and increased the extent of reduction by doping Mn^2+^/Mn^3+^/Mn^4+^ on the B-site and Sr^2+^ on the A-site of LaAlO_3−δ_. La_0.6_Sr_0.4_Mn_0.6_Al_0.4_O_3−δ_ (known as LSMA6464) produced 307.0 μmol g^–1^ of H_2_, surpassing the production of 220.0 μmol g^–1^ achieved by La_0.6_Sr_0.4_Mn_0.4_Al_0.6_O_3−δ_ through a 40% to 60% increase in Mn content. This indicates the importance of Mn inducing higher redox activity. In contrast, La_0.4_Sr_0.6_Mn_0.6_Al_0.4_O_3−δ_ produced 277.0 μmol g^–1^ of H_2_, indicating the negative effect of introducing high amount of Sr^2+^ doping on the A-site.^77,113^ Ann M. Deml et al. put forward the sensitivity of oxygen vacancy formation energies (*E*_v_) to composition. In the Sr_*x*_La_1–*x*_Mn_*y*_Al_1–y_O_3_ composition spectrum, *E*_v_ spans a wide range from near 0 to over 3 eV, offering significant control over the V_o_ formation energetics and redox properties. They observe that *E*_v_ decreases with higher Sr mole fractions (χSr) and either increases or remains constant with increasing Mn mole fractions (χMn). As a result, compositions with lower χSr and higher χMn tend to have higher *E*_v_. Additionally, they also used density functional theory (DFT) to identify LSMA6464 as an optimal LSMA composition.^114^ M. Ezbiri et al. reported that perovskites typically show decreasing enthalpy changes as nonstoichiometry increases, with the most pronounced effect observed in LSMA6464, making it an attractive candidate for H_2_O splitting.^87^ Furthermore, they also measured the equilibrium thermodynamics of La_0.6_Ca_0.4_Mn_0.8_Al_0.2_O_3−δ_ and the perovskite family La_*x*_Sr_1–*x*_Mn_*y*_Al_1–*y*_O_3−δ_ (0 ≤ *x* ≤ 1, 0 ≤ *y* ≤ 1) in the temperature range from 1573 to 1773 K and from 0.206 to 180 mbar O_2_, demonstrating a tunable reduction extent that increases with higher Sr content.^87^ Additionally, Aldo Steinfeld’s group measured the oxygen nonstoichiometry of La_0.6_A_0.4_Mn_1–*y*_Al_*y*_O_3_ (A = Ca, Sr, and *y* = 0, 0.4) under the same temperature conditions as M. Ezbiri et al. and at a range of *P*_O_2__ from 4.5066 × 10^–2^ to 9.9 × 10^–5^ bar. They discovered that the oxygen nonstoichiometry is higher when the Sr^2+^ is replaced with Ca^2+^ in La_0.6_Sr_0.4_MnO_3_, but it also notably increases when 40 mol % Al is doped into the Mn-site.^25^ Berke Piskin’s group also investigated Al-substituted La_0.4_Sr_0.6_Mn_1–*x*_Al_*x*_O_3_ (*x* = 0.4, 0.5, and 0.6). The samples were studied through three continuous thermochemical cycles at reduction/oxidation temperatures of 1350 °C/800 °C. LSMA4646 exhibited the highest H_2_ and O_2_ production of 144 μmol g^–1^ and 275 μmol g^–1^, respectively. This study suggested that increasing the amount of Al can improve hydrogen and oxygen production (as presented in Figure 10), as well as the reoxidation capacity, and enhance structural stability during redox cycling.^115^

In addition, Ezbiri et al. conducted an extensive characterization of the reduction extent capacity of La_1–*x*_Sr_*x*_Mn_*y*_Al_1–*y*_O_3−δ_ perovskites. They observed an increase in oxygen nonstoichiometry with the increase in Sr^2+^ while keeping Al^3+^ constant. However, when Al^3+^ was varied while Sr^2+^ was kept constant, no significant change was observed. The authors also noted the presence of Mn^2+^ surface enrichment in all compositions except for La_0.8_Sr_0.2_Mn_0.8_Al_0.2_O_3−δ_ and La_0.8_Sr_0.2_Mn_0.2_Al_0.8_O_3−δ_, which were the two samples with the lowest oxygen nonstoichiometry. While this observation indicates the redox activity of Mn, samples with a large reduction extent did not necessarily contain a large concentration of Mn^2+^, indicating no correlation between the two. In the absence of Al, Sr surface enrichment occurs rather than Mn enrichment. Ultimately, Mn ions were found to be the redox-active sites for reduction, with Al-doping leading to Mn cation surface enrichment, which are the main enhancers of the reduction extent of Al-doped La_1–*x*_Sr_*x*_MnO_3−δ_ and La_1–*x*_Ca_*x*_MnO_3−δ_.^116^

Consequently, Wang et al. continued to characterize La_1–*x*_Ca_*x*_Mn_1–*y*_Al_*y*_O_3−δ_ for water-splitting applications. Samples with varying Ca^2+^ content (i.e., La_1–*x*_Ca_*x*_Mn_0.6_Al_0.4_O_3−δ_) had an improved O_2_ evolution performance with a positive linear trend and a lower onset temperature for O_2_ evolution with increasing Ca^2+^ concentration. However, H_2_ production peaked at 40% Ca^+^ content and decreased thereafter in a parabola-shaped trend. This anomaly is attributed to the detrimental effect of high Ca content on the thermodynamic water-splitting, since only 30% of the O_2_ released with 80% Ca^3+^ content was recovered. Accordingly, a maximum O_2_ evolution of 419.0 μmol g^–1^ was achieved by La_0.2_Ca_0.6_Mn_0.6_Al_0.4_O_3−δ_, and a maximum H_2_ production of 429.0 μmol g^–1^ was achieved by La_0.6_Ca_0.4_Mn_0.6_Al_0.4_O_3−δ_. Thereafter, the authors varied the Al^3+^ content while maintaining the 40% Ca^3+^ content (i.e., La_0.6_Ca_0.4_Mn_1–*y*_Al_*y*_O_3−δ_), with O_2_ release and H_2_ production increasing with increasing Al^3+^ up until 40% Al^3+^ content and decreasing thereafter. The variation of Al^3+^ had a slight effect on the thermodynamics of the perovskite, as all samples had high reoxidation yields.^117^ As mentioned in the previous section, Lulu Wang et al. also sought enhancement by substituting Mn^3+^ with a Al^3+^ metal ion on the B-site, resulting in an enhanced oxygen release of 184.0 and H_2_ production of 339.0 μmol g^–1^ for La_0.6_Ca_0.4_Mn_0.8_Al_0.2_O_3−δ_.^110^ Moreover, Aldo Steinfeld’s group investigated the solar-to-fuel energy conversion efficiency of La_0.6_Ca_0.4_Mn_0.6_Al_0.4_O_3_ (LCMA) and La_0.6_Sr_0.4_Mn_0.6_Al_0.4_O_3_ (LSMA) and uncovered that investigated materials were more or less sensitive to different parameters, where LCMA and LSMA are associated with gas–gas heat recuperation.^106^

Moreover, Gokon et al. investigated the effects of concurrent Sr^2+^ and Mn ion substitution in La_1–*x*_Sr_*x*_Mn_*x*_Al_1–*x*_O_3−δ_ and concurrent Sr^2+^ and Al^3+^ ion substitution in La_1–*y*_Sr_*y*_Mn_1–*y*_Al_*y*_O_3−δ_ on oxygen release and H_2_ production for thermochemical water-splitting. Accordingly, in La_1–*x*_Sr_*x*_Mn_*x*_Al_1–*x*_O_3−δ_ the concurrent increasing substitution of Sr^2+^ and Mn ions caused the formation of lattice defects in the perovskite structure due to changes in the charge neutrality, which enhanced oxygen release during reduction due to the reaction mechanism involving the Mn^4+^ to Mn^3+^ reduction transition. Under the first thermochemical cycle, 156.3–674.1 μmol g^–1^ of O_2_ was released by the samples, with the amount increasing with the concurrent increase of Sr^2+^ and Mn ion content, corresponding to a H_2_ production of 22.3–129.5 μmol g^–1^ with the maximum occurring at *x* = 0.5. The disparity between the release of the O_2_ and the production of the H_2_ deals with the different mechanisms for each: O_2_ release from a perovskite oxide is an oxygen diffusion process of surface-to-bulk and bulk-to-bulk from the material to an inert atmosphere, while H_2_ production is a chemical reaction of the reduced perovskite oxide with steam. Thus, the samples are not fully reversible.^90^ Recently, Carrillo et al. investigated the oxygen nonstoichiometry and defect equilibria of Y_1–*x*_Sr_*x*_Mn_1–*y*_Al_*y*_O_3−δ_ though reduction experiments conducted at different *P*_O_2__ and temperatures, testing them against the Mn^4+^/Mn^3+^ and Mn^4+^/Mn^2+^ defect models. Accordingly, under low *P*_O_2__, both models fail to fit the reduction extent data of Y_0.8_Sr_0.2_Mn_0.6_Al_0.4_O_3−δ_ and Y_0.9_Sr_0.1_Mn_0.6_Al_0.4_O_3−δ_. Yet, under high *P*_O_2__, both model predictions fit well for Y_0.8_Sr_0.2_Mn_0.6_Al_0.4_O_3−δ_. The authors attribute this disagreement between the models and the data obtained under low *P*_O_2__ to changes in the crystal structure (phase impurities) observed after cycling. Furthermore, for Y_0.8_Sr_0.2_Mn_0.4_Al_0.6_O_3−δ_, the Mn^4+^/Mn^3+^ model succeeded for all measured data under all temperate conditions, under both low and high *P*_O_2__. The authors concluded that the Y_1–*x*_Sr_*x*_Mn_1–*y*_Al_*y*_O_3−δ_ perovskites are not ideal candidates for STCH due to their low sensitivity to reduction under changes in *P*_O_2__. They also state that these samples require a substantial excess of oxidant to achieve high reoxidation rates and hydrogen (H_2_) production.^118^Table 3 presents a detailed overview of STCH performance of MnAl-based perovskite oxides under inert gas conditions.

**Table 3:** STCH Performance of MnAl-Based Perovskite Oxides under Inert Gas Conditions

composition	synthesis method	cycles	Δδ	temp. red./ox. (°C)	time red./ox. (min)	H_2_O (%vol)	gas red./ox.	O_2_ yield (μmol g^–1^)	H_2_ yield (μmol g^–1^)	average H_2_ production rate (μmol g^–1^ min^–1^)	ref
La_0.6_Sr_0.4_Mn_0.6_Al_0.4_O_3−δ_	modified Pechini	1	0.15	1400/-			Ar/O_2_				(112)
La_0.6_Ca_0.4_Mn_0.6_Al_0.4_O_3−δ_	Modified Pechini	1	0.13	1400/-			Ar/O_2_				(112)
La_0.4_Sr_0.6_Mn_0.6_Al_0.4_O_3−δ_;	modified Pechini	1		1350/1000		40.0	He/He		307.0		(77, 113)
La_0.6_Sr_0.4_Mn_0.6_Al_0.4_O_3−δ_;	modified Pechini	1		1350/1000		40.0	He/He		277.0		(77, 113)
La_0.4_Sr_0.6_Mn_0.4_Al_0.6_O_3−δ_	modified Pechini	1		1350/1000		40.0	He/He		220.0		(77, 113)
La_0.8_Ca_0.2_Mn_0.6_Al_0.4_O_3_	sol–gel	1		1400/1000	60/60	40.0	Ar/Ar	176.0	334.0	5.57	(99)
La_0.6_Ca_0.4_Mn_0.6_Al_0.4_O_3_	sol–gel	1		1400/1000	60/60	40.0	Ar/Ar	231.0	429.0	7.15	(99)
La_0.4_Ca_0.6_Mn_0.6_Al_0.4_O_3_	sol–gel	1		1400/1000	60/60	40.0	Ar/Ar	280.0	364.0	6.07	(99)
La_0.2_Ca_0.8_Mn_0.6_Al_0.4_O_3_	sol–gel	1		1400/1000	60/60	40.0	Ar/Ar	419.0	260.0	4.33	(99)
La_0.6_Ca_0.4_Mn_0.8_Al_0.2_O_3−δ_	Pechini	1		1300/900	60/60	40.0	Ar/Ar	184.0	339.0	5.65	(110)
La_0.8_Sr_0.2_Mn_0.8_Al_0.2_O_3−δ_	Pechini	1	0.034	1350/-	225/-		Ar/-				(116)
La_0.6_Sr_0.4_Mn_0.8_Al_0.2_O_3−δ_	Pechini	1	0.116	1350/-	225/-		Ar/-				(116)
La_0.4_Sr_0.6_Mn_0.8_Al_0.2_O_3−δ_	Pechini	1	0.270	1350/-	225/-		Ar/-				(116)
La_0.2_Sr_0.8_Mn_0.8_Al_0.2_O_3−δ_	Pechini	1	0.351	1350/-	225/-		Ar/-				(116)
La_0.8_Sr_0.2_Mn_0.6_Al_0.4_O_3−δ_	Pechini	1	0.028	1350/-	225/-		Ar/-				(116)
La_0.6_Sr_0.4_Mn_0.6_Al_0.4_O_3−δ_	Pechini	1	0.171	1350/-	225/-		Ar/-				(116)
La_0.4_Sr_0.6_Mn_0.6_Al_0.4_O_3−δ_	Pechini	1	0.208	1350/-	225/-		Ar/-				(116)
La_0.8_Sr_0.2_Mn_0.4_Al_0.6_O_3−δ_	Pechini	1	0.035	1350/-	225/-		Ar/-				(116)
La_0.6_Sr_0.4_Mn_0.4_Al_0.6_O_3−δ_	Pechini	1	0.134	1350/-	225/-		Ar/-				(116)
La_0.8_Sr_0.2_Mn_0.2_Al_0.8_O_3−δ_	Pechini	1	0.049	1350/-	225/-		Ar/-				(116)
La_0.6_Ca_0.4_Mn_0.6_Al_0.4_O_3−δ_	Pechini	1	0.153	1350/-	225/-		Ar/-				(116)
La_0.6_Ca_0.4_Mn_0.8_Al_0.2_O_3−δ_	Pechini	1		1400/1000	60/60	40.0	Ar/Ar	201.0	374.0	0	(117)
La_0.8_Ca_0.2_Mn_0.6_Al_0.4_O_3−δ_	Pechini	1		1400/1000	60/60	40.0	Ar/Ar	176.0	334.0	0	(117)
La_0.6_Ca_0.4_Mn_0.6_Al_0.4_O_3−δ_	Pechini	5		1400/1000	60/60	40.0	Ar/Ar	231.0	429.0	0	(117)
La_0.4_Ca_0.6_Mn_0.6_Al_0.4_O_3−δ_	Pechini	1		1400/1000	60/60	40.0	Ar/Ar	280.0	364.0	0	(117)
La_0.2_Ca_0.8_Mn_0.6_Al_0.4_O_3−δ_	Pechini	1		1400/1000	60/60	40.0	Ar/Ar	419.0	260.0	0	(117)
La_0.6_Ca_0.4_Mn_0.4_Al_0.6_O_3−δ_	Pechini	1		1400/1000	60/60	40.0	Ar/Ar	186.0	349.0	0	(117)
La_0.6_Ca_0.4_Mn_0.2_Al_0.8_O_3−δ_	Pechini	1		1400/1000	60/60	40.0	Ar/Ar	126.0	239.0	0	(117)
La_0.9_Sr_0.1_Mn_0.9_Al_0.1_O_3−δ_	Pechini	3		1350/1000	1/60	84.0	N_2_/N_2_	55.2^a^	72.1^a^	0	(90)
La_0.8_Sr_0.2_Mn_0.8_Al_0.2_O_3−δ_	Pechini	3		1350/1000	1/60	84.0	N_2_/N_2_	85.2^a^	76.8^a^	0	(90)
La_0.7_Sr_0.2_Mn_0.7_Al_0.3_O_3−δ_	Pechini	3		1350/1000	1/60	84.0	N_2_/N_2_	107.5^a^	125.0^a^	0	(90)
La_0.3_Sr_0.7_Mn_0.7_Al_0.3_O_3−δ_	Pechini	2		1350/1000	1/60	50.0	N_2_/N_2_	674.1^a^	69.3^a^	0	(90)
La_0.3_Sr_0.7_Mn_0.7_Al_0.3_O_3−δ_	Pechini	1		1350/1000	1/60	84.0	N_2_/N_2_		116.1^a^	1.94	(90)
La_0.6_Sr_0.4_Mn_0.6_Al_0.4_O_3−δ_	Pechini	3		1350/1000	1/60	84.0	N_2_/N_2_	103.4^a^	58.7^a^	0.98	(90)
La_0.4_Sr_0.6_Mn_0.6_Al_0.4_O_3−δ_	Pechini	2		1350/1000	1/60	50.0	N_2_/N_2_	535.7^a^	75.7^a^	1.26	(90)
La_0.4_Sr_0.6_Mn_0.6_Al_0.4_O_3−δ_	Pechini	1		1350/1000	1/60	84.0	N_2_/N_2_		147.3^a^	2.46	(90)
La_0.5_Sr_0.5_Mn_0.5_Al_0.5_O_3−δ_	Pechini	2		1350/1000	1/60	50.0	N_2_/N_2_	405.5^a^	129.5^a^	2.16	(90)
La_0.5_Sr_0.5_Mn_0.5_Al_0.5_O_3−δ_	Pechini	1		1350/1000	1/60	84.0	N_2_/N_2_		129.5^a^	2.16	(90)
La_0.6_Sr_0.4_Mn_0.4_Al_0.6_O_3−δ_	Pechini	2		1350/1000	1/60	50.0	N_2_/N_2_	338.5^a^	66.5^a^	1.11	(90)
La_0.7_Sr_0.3_Mn_0.3_Al_0.7_O_3−δ_	Pechini	2		1350/1000	1/60	50.0	N_2_/N_2_	156.3^a^	22.3^a^	0.37	(90)
La_0.6_Sr_0.4_Mn_0.6_Al_0.4_O_3−δ_	Pechini	3	0.086	1400/1000	5.5/20	40.0	Ar/Ar		292.0	14.60	(26)
La_0.6_Sr_0.4_Mn_0.6_Al_0.4_O_3−δ_	Pechini	1		1400/850	5.5/20	40.0	Ar/Ar		256.0	12.80	(26)
La_0.6_Sr_0.4_Mn_0.6_Al_0.4_O_3−δ_	Pechini	1		1400/750	5.5/20	40.0	Ar/Ar		205.0	10.25	(26)
La_0.6_Sr_0.4_Mn_0.6_Al_0.4_O_3−δ_	Pechini	1		1350/1000	5.5/20	40.0	N_2_/N_2_		224.0	11.20	(26)
La_0.6_Sr_0.4_Mn_0.6_Al_0.4_O_3−δ_	Pechini	3		1350/850	5.5/20	40.0	Ar/Ar	163.0	194.0	9.70	(26)
La_0.6_Sr_0.4_Mn_0.6_Al_0.4_O_3−δ_	Pechini	1		1350/850	5.5/20	40.0	Ar/Ar–H_2_^b^		54.7	2.74	(26)
La_0.6_Sr_0.4_Mn_0.6_Al_0.4_O_3−δ_	Pechini	1		1350/850	5.5/20	40.0	Ar/Ar–H_2_^c^		32.4	1.62	(26)
La_0.6_Sr_0.4_Mn_0.6_Al_0.4_O_3−δ_	Pechini	1		1350/850	5.5/20	40.0	Ar/Ar–H_2_^d^		27.7	1.39	(26)
La_0.6_Sr_0.4_Mn_0.6_Al_0.4_O_3−δ_	Pechini	1		1350/850	5.5/20	40.0	Ar/Ar–H_2_^e^		2.4	0.12	(26)
La_0.6_Sr_0.4_Mn_0.6_Al_0.4_O_3−δ_	Pechini	1		1350/850	5.5/20	40.0	Ar/Ar–H_2_^f^		0.0	0	(26)
La_0.6_Sr_0.4_Mn_0.6_Al_0.4_O_3−δ_	Pechini	1		1350/750	5.5/20	40.0	Ar/Ar		150.0	7.50	(26)
La_0.6_Sr_0.4_Mn_0.6_Al_0.4_O_3−δ_	Pechini	1		1250/1000	5.5/20	40.0	Ar/Ar		105.0	5.25	(26)
La_0.6_Sr_0.4_Mn_0.6_Al_0.4_O_3−δ_	Pechini	1		1250/850	5.5/20	40.0	Ar/Ar		89.0	4.45	(26)
La_0.6_Sr_0.4_Mn_0.6_Al_0.4_O_3−δ_	Pechini	1		1250/750	5.5/20	40.0	Ar/Ar		74.0	3.70	(26)
La_0.6_Sr_0.4_Mn_0.6_Al_0.4_O_3−δ_	Combustion	3	0.040	1350/800	40/-	100.0	Ar/-	103.0	254.0		(119)

a Value converted from mL g_material_^–1^ (Ncm^3^ g_material_^–1^). Standard volume occupied: 22400 mL mol^–1^).

b H_2_O:H_2_ = 1333.

c H_2_O:H_2_ = 1000.

d H_2_O:H_2_ = 750.

e H_2_O:H_2_ = 500.

f H_2_O:H_2_ = 285.

Table 4 presents an overview of the STCH performance of MnAl-based perovskite oxides under reducing gas conditions.

**Table 4:** STCH Performance of MnAl-Based Perovskite Oxides under Reducing Gas Conditions

composition	synthesis method	cycles	Δδ	temp. red./ox. (°C)	time red./ox. (min)	gas red./ox.	average H_2_ production rate (μmol g^–1^ min^–1^)	ref
Y_0.8_Sr_0.2_Mn_0.6_Al_0.4_O_3−δ_	Pechini	8	0.3925	900/900	1/5	aAr–H_2_/Ar–O_2_	0	(118)
Y_0.8_Sr_0.2_Mn_0.4_Al_0.6_O_3−δ_	Pechini	8	0.3319	900/900	1/5	aAr–H_2_/Ar–O_2_	0	(118)
Y_0.9_Sr_0.1_Mn_0.6_Al_0.4_O_3−δ_	Pechini	8	0.3388	900/900	1/5	aAr–H_2_/Ar–O_2_	0	(118)

a Injection of Ar–H_2_ (*P*_H_2__ = 0.01 atm) began at 300 °C. Injection of Ar–O_2_ (*P*_O_2__ = 0.15 atm) continued with cooldown to 300 °C.

### 5.3 MnX-Based Perovskite Oxides As Redox-Active Oxygen Exchange Materials

In addition to investigating Al doping in the Mn site, researchers have also explored doping with other elements (X = Ce, Sc, Ga, Cr, Fe, Co, Ti, and Mg) in the Mn site. For example, Ryan P. O’Hayre’s group discovered that BaCe_0.25_Mn_0.75_O_3_ (BCM) exhibits nearly three times the amount of hydrogen compared to ceria when reduced at 1350 °C. Furthermore, this material demonstrates a greater tendency for H_2_O splitting and faster oxidation kinetics compared to the Mn-based perovskite La_1–*x*_Sr_*x*_Mn_*y*_Al_1–*y*_ (*x*, *y* = 0.4, 0.6).^26^ Additionally, Trindell et al. also investigated the potential of BaCe_0.25_Mn_0.75_O_3_ (BCM) as an active material for solar thermochemical processes. Previous studies on BCM have faced challenges due to the presence of secondary phases, which hindered the analysis of stability and performance. This study presents two synthesis approaches for producing nearly single-phase samples of BCM: solid-state and sol–gel routes. It demonstrates that both methods can yield a high-purity 12R hexagonal perovskite phase of BCM (>97 wt %). The study also examines the anisotropy of thermal expansion in BCM and investigates the impact of high-temperature redox cycling on its stability and phase fraction. The results provide valuable insights into the synthesis and performance of BCM for solar thermochemical applications.^120^ In addition, the 12R hexagonal perovskite structure of BCM exhibits an interesting characteristic wherein it tends to partially transform into a 6H polytype when exposed to high temperatures and reducing environments. This transformation occurs during the initial stage of the thermochemical cycle and can help to prevent the degradation of this complex oxide material. An analogous compound, BaNb_0.25_Mn_0.75_O_3_ (BNM), which also possesses a 12R structure, was successfully synthesized. Remarkably, BNM undergoes almost complete conversion into the 6H structure under the same reaction conditions as BCM.^121^ Moreover, Roychoudhury et al. investigated the electronic structure of BaCe_0.25_Mn_0.75_O_3−δ_ before and after thermal reduction using a combination of experimental K-edge X-ray absorption spectroscopy (XAS) and first-principles calculations. The computed projected density of states (PDOS) and orbital plots propose a simplified model that elucidates the interaction between oxygen and metal atoms within the material. Experimental measurements confirm a noticeable reduction in the intensity of the first peak in the O K-edge spectrum for the reduced crystal due to oxygen removal.^122^

Debora R. Barcellos et al.’s initial attempt to synthesize B-site doping of SrMnO_3−δ_ by Ce led to the development of the novel Ruddlesden–Popper (layered perovskite) phase discussed previously. SrCe_*y*_Mn_1–*y*_O_3_ (y = 0, 0.3, 0.5, 0.7, 1.0) were investigated. In all cases, a mixture of SrCeO_3_ and/or SrMnO_3_, CeO_2_, and Ce_*x*_Sr_2–*x*_MnO_4_ was developed as secondary phases though the compositional range, despite the tolerance factor calculation predicting a cubic phase for doped samples. Two samples were tested for STCH production. The first one, *y* = 0.5, contained 8% SrCeO_3_, 53% Ce_*x*_Sr_2–*x*_MnO_4_, 27% CeO_2_, and 12% SrMnO_3_ and produced 140.0 μmol g^–1^ of H_2_. The second one, *y* = 0.7, contained 52% SrCeO_3_, 33% Ce_*x*_Sr_2–*x*_MnO_4_, and 15% CeO_2_, and it produced 100.0 μmol g^–1^ of H_2_. As a result, only CeO_2_ and the RP phase are thermochemically active, with the latter one showing higher activity as a higher concentration presented a higher H_2_ yield.^108^ Su Jeong Heo et al. investigated the possibility of double Ce substitution of the (Ba,Sr)MnO_3−δ_ perovskite (i.e., (Ba,Sr)_1–*x*_Ce_*x*_Ce_*y*_Mn_1–*y*_O_3−δ_) following the results obtained from Débora R. Barcellos et al. The initial models of single- and double-site substitution using the Goldschmidt tolerance factor were applied to the composition space of a (Ba,Sr)_1–*x*_Ce_*x*_Ce_*y*_Mn_1–*y*_O_3−δ_ quaternary phase diagram and a BaMnO_3_–SrMnO_3_–CeO_2_ ternary phase diagram, respectively. These models demonstrated the possible structural changes induced in (Ba,Sr)MnO_3−δ_ due to reductions in the tolerance factor. The authors concluded that the double-site substitution of Ce in (Ba,Sr)MnO_3−δ_ expands the composition range of stability for 10H (e.g., BaCe_0.25_Mn_0.75_O_3−δ_) and 3C (e.g., Sr_0.75_Ce_0.25_MnO_3−δ_) perovskite structures, which are known to be suitable for STCH applications. Moreover, the introduction of Sr^2+^ into Ba(Ce,Mn)O_3_ increases the occurrence of 10H phase formation from 25% Ce to approximately 32% Ce, overcoming the restriction of having an exact Ce/Mn ratio of 0.25/0.75 for BaCe_0.25_Mn_0.75_O_3−δ_.^123^ In recent years, E. A. Carter’s research group has specifically considered the incorporation of only Ce and/or Ca on the A-site of perovskite structures due to their similar ionic sizes and the potential redox activity of Ce^4+^. Furthermore, they have identified promising candidates, including Ca_0.5_Ce_0.5_MnO_3_, Ca_0.5_Ce_0.5_FeO_3_, and Ca_0.5_Ce_0.5_VO_3_, based on their favorable oxygen vacancy formation energies and thermodynamic stability. Notably, Ca_0.5_Ce_0.5_MnO_3_ stands out as an exceptional candidate for STCH applications. This compound exhibits simultaneous reduction of both Ce^4+^ on the A-site and Mn^3+^ on the B-site, making it particularly promising due to its predicted higher reduction entropy compared to CeO_2_.^124^ They have also introduced a new redox-active perovskite, Ca_2/3_Ce_1/3_Ti_1/3_Mn_2/3_O_3_, which exhibits a high-purity phase. Experimental and modeling results have demonstrated that the main redox-active species in this perovskite is Ce^4+^, undergoing a reduction from Ce^4+^ to Ce^3+^. This marks the first report of a perovskite with both a reducible A-site and reducible Ce^4+^, which distinguishes it from other known reducible perovskites where the active element is invariably located on the B-site.^125^

C. N. R. Rao’s group first explored La_0.5_Sr_0.5_Mn_0.95_Sc_0.05_O_3_ in thermochemical water-splitting. They reported that substitution of even 5% Sc^3+^ (*x* = 0.05) results in an outstanding performance. For instance, the yield of the O_2_ is almost twice that of the parent perovskite La_0.5_Sr_0.5_MnO_3_. Furthermore, La_0.5_Sr_0.5_Mn_0.95_Sc_0.05_O_3_ produced 250 μmol g^–1^ of H_2_ at 1100 °C.^126^ In addition, Lulu Wang et al. also conducted an investigation involving the doping of Ga^3+^ ions on the B-site of perovskites. This study led to the discovery of a novel perovskite composition, La_0.6_Ca_0.4_Mn_0.8_Ga_0.2_O_3_, exhibiting an exceptional water-splitting performance. It generated a noteworthy 401 μmol g^–1^ of H_2_ at relatively low thermochemical cycle temperatures between 1300 and 900 °C.^110^ Remarkably, this as-prepared perovskite demonstrated 12× higher H_2_ production compared to the benchmark CeO_2_ catalyst under the same experimental conditions. Additionally, this innovative perovskite demonstrated remarkable stability and steady-state redox activity throughout the water-splitting cycles.^110^

La_0.7_Sr_0.3_Mn_1–*z*_Cr_*z*_O_3_ was examined by Nobuyuki Gokon et al. They reported that lower amounts of Cr (*z* = 0.1 and 0.2) promote oxidation kinetics, and a small Cr content in LaSrMnO_3_ can enhance the overall thermochemical efficiency.^90^ They explored the influence of the thermal reduction temperatures of 1000–1350 °C on O_2_/H_2_ productivity and repeatability with water-splitting at 1200 °C. La_0.7_Sr_0.3_Mn_0.9_Cr_0.1_O_3_ exhibited notable oxygen evolution rates within the temperature range of 1350–1300 °C, while demonstrating comparable rates in the range of 1000–1200 °C. Following treatment at temperatures of 1350–1300 °C during the thermal reduction (TR) process, the resulting samples exhibited superior hydrogen production rates. Conversely, during TR at 1000–1200 °C, the sample exhibited favorable reactivity and consistency in both oxygen release and hydrogen production, even at lower production levels, without encountering coagulation or sintering issues.^127^ Berke Piskin’s group investigated the effect of a range of calcium concentrations (*x* = 0, 0.2, 0.4, 0.6, 0.8) in La_1–*x*_Ca_*x*_Mn_0.8_Co_0.2_O_3_ (LCMC) perovskite-type oxide and explored the hydrogen production about their kinetics, structural properties, H_2_/O_2_ production capacities, and cyclabilities. Among these compositions, LCMC6282 and LCMC8282 displayed hydrogen productions of 88 and 256 μmol g^–1^, respectively. However, all materials examined suffer from poor cyclability.^128^

In addition to the study of La_0.6_Sr_0.4_MnO_3_ presented earlier, Luciani et al. studied La_0.6_Sr_0.4_Mn_1–*x*_Fe_*x*_O_3_ (*x* = 0, 0.2, 0.4, and 0.6) for STCH applications via reduction through the usage of H_2_ balanced in N_2_. Thus, *x* = 0 presented a tetragonal structure, *x* = 0.2 represented the coexistence of tetragonal and orthorhombic structures, *x* = 0.4 and *x* = 0.6 represented single rhombohedral structures, and *x* = 1.0 presented both rhombohedral and tetragonal structures with a minor presence of the SrLaFeO_4_ phase. *x* = 1.0 was the most reducible of the samples, showing both the lowest reduction temperature (i. e., 400 °C) and the highest O_2_ production. For *x* = 0.4 and *x* = 0.6, the start of reduction occurred at temperatures higher that for *x* = 0, with the presence of a small reduction phenomenon at low temperatures becoming more apparent with increasing Fe content. The reduced amount of released oxygen for *x* = 0.2 and *x* = 0.4 was ascribed to the preferential substitution of Fe^3+^ ions to Mn^4+^ species, which accounts for the decrease in the overall reduction behavior. After water-splitting tests, the secondary phase (i.e., SrLaFeO_4_) in *x* = 1.0 disappeared while the tetragonal phase became dominant; for *x* = 0, the presence of both tetragonal and hexagonal phases demonstrated that reduction in a hydrogen atmosphere leads to a phase transition in the perovskite structure. The authors highlighted that no reduced phase is detected after H_2_ reduction; rather, any change is related to the conversion from one perovskite structure to another. Production of H_2_ steadily increases with increasing Fe content, with the onset temperature of H_2_ production also increasing with the Fe content, while oxidation is preferred by the Mn content through a decrease in the onset temperature of O_2_ evolution.^96^ LaFe_1–*y*_Mn_*y*_O_3_ (*y* = 0, 0.05, 0.1, 0.2, 0.3, 0.4, 0.5) (25 wt %)/SiO_2_ were investigated by Zhenpan Chen et al. Relative to undoped LaFeO_3_/SiO_2_, the addition of approximately 5 at. % Mn leads to an approximately 20% increase in the maximum rate of oxygen release. However, with higher Mn doping levels, the maximum oxygen release rate begins declining. Concurrently, the highest rate of hydrogen release experiences a gradual reduction as the Mn content increases, attributed to the diminishing thermodynamic impetus behind the water-splitting reaction.^129^ G. Luciani et al. also explored La_0.6_Sr_0.4_Mn_1–*x*_Fe_*x*_O_3−δ_ (*x* = 0.2, 0.4, and 0.6). The increase of the iron content corresponds to a progression in the reduction stage, as indicated by the trend of the initial reduction temperatures. Conversely, heightened levels of manganese appear to enhance the activity to water-splitting.^96^

**Figure 11:**
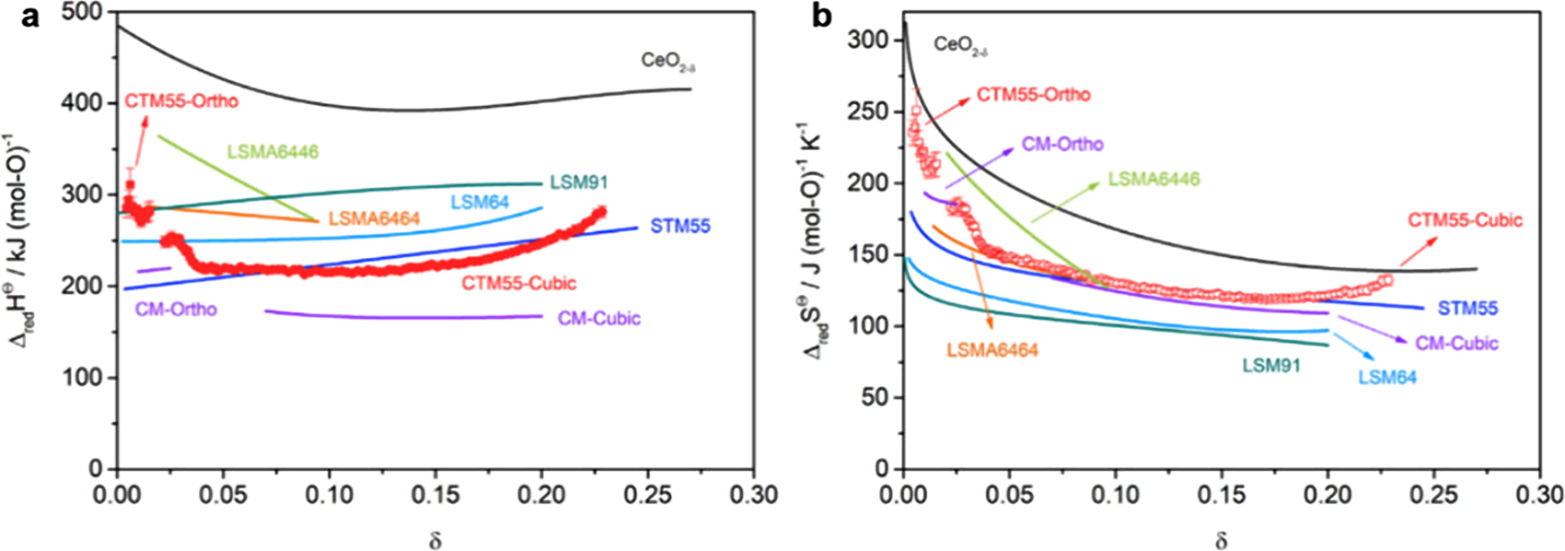
(a) Standard enthalpy of reduction and (b) standard entropy of reduction for a series of oxides, including CaMnO_3_ (CM) and SrTi_0.5_Mn_0.5_O_3−δ_ (STM55).^66^ Reproduced from ref (66). Available under a Creative Commons CC-BY-NC-ND license. Copyright 2020 Qian and co-workers.

Xin Qian et al. explored the redox thermodynamics of SrTi_0.5_Mn_0.5_O_3−δ_ for water-splitting. The authors mention that a material that readily releases oxygen in the thermal reduction step will typically have a small driving force for reoxidation by steam during the water-splitting process. Under nonisothermal conditions, the previously mentioned contradictions can be addressed by developing a material with a significant entropy of reduction, primarily driven by configurational entropy arising from the creation of oxygen vacancies. Regarding the entropy of reduction, that of the sample is comparable to those of undoped or lightly B-site-doped alkaline manganates. Additionally, it is substantially larger than that of La_0.6_Sr_0.4_MnO_3_, which is a material known for its suitability in STCH applications. The enthalpy of reduction for SrTi_0.5_Mn_0.5_O_3−δ_ was found to be higher than those of undoped or lightly B-site-doped alkaline manganates such as CaMnO_3_ (i.e., 161–178 kJ (mol-O)^−1^), as shown in Figure 11, and SrMnO_3_ (i.e., 147 kJ (mol-O)^−1^).^66^

Upon multiple cycles, three features became evident: (1) oxygen release is faster than hydrogen production, as expected from the quasi-equilibrium behavior and thermodynamic properties of the material; (2) there is no evidence of decay in hydrogen production between cycles; and (3) the H_2_/O_2_ ratio remains consistently close to 2:1 for all cycles, indicating the full utilization of oxygen vacancies generated during reduction.^127^ On the next year, the same group of authors (i.e., Xin Qian et al.) explored the properties and STCH performance of CaTi_0.5_Mn_0.5_O_3−δ_. The perovskite adopted the GdFeO_3_-type phase with an orthorhombic distortion, with a random arrangement of Ti and Mn on the B-site. The behavior of CaTi_0.5_Mn_0.5_O_3−δ_ exactly resembled that of CaMnO_3_.^66^ CaMnO_3_ is considered a promising candidate in thermochemical heat storage, as it can reversibly release oxygen over a wide range of oxygen partial pressures and temperatures. However, it undergoes decomposition at a temperature of ≥1100 °C and *P*_O_2__ ≤ 0.008 atm. Sossina M. Haile’s group explored two doping levels, *x* = 0.1 and 0.3, in CaFe_*x*_Mn_1–*x*_O_3−δ_ to overcome this shortage and extend the operating temperature. They discovered that CaFe_0.1_Mn_0.9_O_3−δ_ can prevent CaMnO_3_ decomposition up to 1200 °C at *P*_O_2__ = 0.008 atm.^67^ In situ analysis in air of CaTi_0.5_Mn_0.5_O_3−δ_ showed the reversible transitions between the orthorhombic and cubic phases, with the latter being the only phase present after 1200 °C. Ex situ analysis revealed a minimum stability condition of 1600 °C under air and 1450 °C under *P*_O_2__ = 4.20 × 10^–5^ atm for the sample, with the latter showing the presence of stoichiometric CaTiO_3_ and Ca_0.5_Mn_0.5_O that implied the complete reduction of Mn into the 2+ oxidation state while Ti retained its 4+ oxidation state. Through the mass loss profile studies described earlier, both the reduction enthalpy and reduction entropy of the orthorhombic phase are larger than those of the cubic phase, with the entropy approaching that of CeO_2−δ_ and similar to that of La_0.6_Sr_0.4_Mn_0.4_Al_0.6_O_3−δ_, and the enthalpy being moderate and approaching those of La_0.6_Sr_0.4_Mn_0.6_Al_0.4_O_3−δ_ and La_0.9_Sr_0.1_MnO_3−δ_. Nevertheless, the cubic region showed thermodynamic properties similar to that of Sr_0.5_Ti_0.5_MnO_3−δ_, suggesting a cubic or near-cubic perovskite presents a low enthalpy of reduction and is the phase present under high δ, as seen under STCH conditions. Overall, the instruction of Ti into the CaMnO_3_ perovskite increased the enthalpy while having little effect on the entropy, which is highly desirable for water-splitting applications.^66^ Greta R. Patzke’s group investigated the potential of cation-deficient oxides (Ce_*x*_Sr_1–*x*_)_0.95_Ti_0.5_Mn_0.5_O_3−δ_ (*x* = 0, 0.10, 0.15, and 0.20; CSTM) as the redox materials for solar-driven thermochemical (STC) fuel production. With the substitution of Ce, the enthalpy and entropy change increase simultaneously. (Ce_0.2_Sr_0.8_)_0.95_Ti_0.5_Mn_0.5_O_3−δ_ (CSTM20) has a combination of a moderate enthalpy change of about 238 kJ (mol-O)^−1^ and a large entropy change of about 141 J(mol-O)^−1^K^–1^, which created a favorable thermodynamic property for H_2_O splitting. Both Ce and Mn are redox-active centers that experience concurrent reduction during redox cycling, as confirmed by X-ray absorption spectroscopy (XAS).^130^

La_0.5_Sr_0.5_Mn_0.9_Mg_0.1_O_3_ was investigated as a perovskite coating by Haeussler et al. due to its thermochemical stability and high fuel productivity. Anita Haeussler et al. focused on the thermochemical performance of a composite reactive material composed of reticulated ceria foam with a uniform perovskite coating consisting of La_0.5_Sr_0.5_Mn_0.9_Mg_0.1_O_3_ to enhance the reduction extent in comparison with pure ceria. Ceria, in this regard, has limitations due to its moderate reduction extent at temperatures up to 1400 °C, while perovskites offer a higher oxygen exchange capacity under similar conditions. Nevertheless, the reoxidation of perovskites is slow and incomplete due to their thermodynamic limitations, whereas ceria reoxidation is thermodynamically more favorable. Therefore, the addition of a thin layer of perovskite to ceria should enhance its reduction ability, as the perovskite is expected to promote oxygen diffusion within ceria.^131^ Two year later, Haeussler et al. explored a wide range of ABO_3_ compositions (with A = La, Y, Sr, Ca, Pr, Ce, Sm and B = Co, Mn, Fe, Al, Ga, Mg, Cr) to identify suitable formulations for thermochemical cycles that could potentially achievea high solar-to-fuel conversion efficiency. Stéphane Abanades’s group also investigated La_0.5_Sr_0.5_Mn_0.9_Mg_0.1_O_3_ and discovered that the presence of a Mg^2+^ ion can stabilize the perovskite structure, as the thermochemical cycles measured in TGA did not induce sintering. Mg is promising as a dopant, it can enhance the oxidation degree of Mn even though Mg^2+^ is not involved in redox reactions.^132^Table 5 provides a comprehensive overview of the solar thermochemical hydrogen (STCH) production performance of MnX-based perovskite oxides when subjected to inert gas conditions.

**Table 5:** STCH Performance of MnX-Based Perovskite Oxides under Inert Gas Conditions

composition	synthesis method	cycles	Δδ	temp. red./ox. (°C)	time red./ox. (min)	H_2_O (%vol)	gas red./ox.	O_2_ yield (μmol g^–1^)	H_2_ yield (μmol g^–1^)	average H_2_ production rate (μmol g^–1^ min^–1^)	ref
BaCe_0.05_Mn_0.95_O_3−δ_	Pechini		0.345				N_2_/air				(26)
BaCe_0.15_Mn_0.85_O_3−δ_	Pechini		0.228				N_2_/air				(26)
BaCe_0.25_Mn_0.75_O_3−δ_	Pechini	3	0.183	1400/1000	5.5/20	40.0	Ar/Ar		181.0	9.05	(26)
BaCe_0.25_Mn_0.75_O_3−δ_	Pechini	3		1400/850	5.5/20	40.0	Ar/Ar		165.0	8.25	(26)
BaCe_0.25_Mn_0.75_O_3−δ_	Pechini	3	0.183	1400/750	5.5/20	40.0	Ar/Ar		136.0	6.80	(26)
BaCe_0.25_Mn_0.75_O_3−δ_	Pechini	3		1350/1000	5.5/20	40.0	Ar/Ar		146.0	7.30	(26)
BaCe_0.25_Mn_0.75_O_3−δ_	Pechini	3		1350/850	5.5/20	40.0	Ar/Ar	82.0	140.0	7.00	(26)
BaCe_0.25_Mn_0.75_O_3−δ_	Pechini	50		1350/850	3.5/10	40.0	Ar/Ar	65.0	138.0	13.80	(26)
BaCe_0.25_Mn_0.75_O_3−δ_	Pechini	1		1350/850	5.5/20	40.0	Ar/Ar–H_2_^b^		98.2	4.91	(26)
BaCe_0.25_Mn_0.75_O_3−δ_	Pechini	1		1350/850	5.5/20	40.0	Ar/Ar–H_2_^c^		93.5	4.68	(26)
BaCe_0.25_Mn_0.75_O_3−δ_	Pechini	1		1350/850	5.5/20	40.0	Ar/Ar–H_2_^d^		67.1	3.36	(26)
BaCe_0.25_Mn_0.75_O_3−δ_	Pechini	1		1350/850	5.5/20	40.0	Ar/Ar–H_2_^e^		64.1	3.21	(26)
BaCe_0.25_Mn_0.75_O_3−δ_	Pechini	1		1350/850	5.5/20	40.0	Ar/Ar–H_2_^f^		26.5	1.33	(26)
BaCe_0.25_Mn_0.75_O_3−δ_	Pechini	3		1350/750	5.5/20	40.0	Ar/Ar		99.0	4.95	(26)
BaCe_0.25_Mn_0.75_O_3−δ_	Pechini	3		1250/1000	5.5/20	40.0	Ar/Ar		86.0	4.30	(26)
BaCe_0.25_Mn_0.75_O_3−δ_	Pechini	3		1250/850	5.5/20	40.0	Ar/Ar		56.0	2.80	(26)
BaCe_0.25_Mn_0.75_O_3−δ_	Pechini	3		1250/750	5.5/20	40.0	Ar/Ar		52.0	2.60	(26)
BaCe_0.50_Mn_0.50_O_3−δ_	Pechini	3	0.104	1350/1000	5.5/20	40.0	Ar/Ar		97.0	4.85	(26)
BaCe_0.75_Mn_0.25_O_3−δ_	Pechini	3	0.158	1350/1000	5.5/20	40.0	Ar/Ar		40.5	2.03	(26)
SrCe_0.3_Mn_0.7_O_3−δ_	Pechini	1		1400/1000	5.5/16.7	50.0	Ar/Ar				(108)
SrCe_0.5_Mn_0.5_O_3−δ_	Pechini	1		1400/1000	5.5/16.7	50.0	Ar/Ar		140.0	8.38	(108)
SrCe_0.7_Mn_0.3_O_3−δ_	Pechini	1		1400/1000	5.5/16.7	50.0	Ar/Ar		100.0	5.99	(108)
Sr_0.75_Ce_0.25_MnO_3−δ_	Pechini	3		1400/1000	5.5/20	40.0	Ar/Ar	104.0	224.0	11.20	(123)
Sr_0.75_Ce_0.25_MnO_3−δ_	Pechini	3		1350/850	5.5/20	40.0	Ar/Ar	68.0	98.0	4.90	(123)
La_0.5_Sr_0.5_Mn_0.95_Sc_0.05_O_3−δ_	Pechini	3	0.21	1400/1100	45/		Ar/-	417.0			(126)
La_0.5_Sr_0.5_Mn_0.95_Sc_0.05_O_3−δ_	Pechini	1	0.21	1400/1100	45/100		N_2_/N_2_	390.0	250.0	2.50	(126)
La_0.5_Sr_0.5_Mn_0.9_Sc_0.1_O_3−δ_	Pechini	1	0.21	1400/1100	45/		Ar/-	426.0			(126)
La_0.6_Ca_0.4_Mn_0.8_Ga_0.2_O_3−δ_	Pechini	6		1300/900	60/60	40.0	Ar/Ar	212.0	401.0	6.68	(110)
La_0.6_Ca_0.4_Mn_0.8_Ga_0.2_O_3−δ_	Pechini	1		1400/900	60/60	40.0	Ar/Ar	320.0	473.0	7.88	(110)
La_0.7_Sr_0.3_Mn_0.9_Cr_0.1_O_3−δ_	Pechini	3		1350/1000	1^a^/60	84.0	N_2_/N_2_	96.2^g^	145.8^g^	2.43	(90)
La_0.7_Sr_0.3_Mn_0.9_Cr_0.1_O_3−δ_	Pechini	3		1350/1200	1^a^/60	84.0	N_2_/N_2_	107.6^g^	160.9^g^	2.68	(90)
La_0.7_Sr_0.3_Mn_0.8_Cr_0.2_O_3−δ_	Pechini	3		1350/1000	1^a^/60	84.0	N_2_/N_2_	97.7^g^	113.9^g^	1.90	(90)
La_0.7_Sr_0.3_Mn_0.8_Cr_0.2_O_3−δ_	Pechini	3		1350/1100	1^a^/60	84.0	N_2_/N_2_	106.8^g^	133.0^g^	2.22	(90)
La_0.7_Sr_0.3_Mn_0.8_Cr_0.2_O_3−δ_	Pechini	3		1350/1200	1^a^/60	84.0	N_2_/N_2_	102.3^g^	160.2^g^	2.67	(90)
La_0.7_Sr_0.3_Mn_0.7_Cr_0.3_O_3−δ_	Pechini	3		1350/1000	1^a^/60	84.0	N_2_/N_2_	86.7^g^	123.9^g^	2.07	(90)
La_0.7_Sr_0.3_Mn_0.6_Cr_0.4_O_3−δ_	Pechini	3		1350/1000	1^a^/60	84.0	N_2_/N_2_	87.7^g^	93.4^g^	1.56	(90)
La_0.7_Sr_0.3_Mn_0.5_Cr_0.5_O_3−δ_	Pechini	3		1350/1000	1^a^/60	84.0	N_2_/N_2_	85.3^g^	73.8^g^	1.23	(90)
La_0.7_Sr_0.3_Mn_0.3_Cr_0.7_O_3−δ_	Pechini	3		1350/1000	1^a^/60	84.0	N_2_/N_2_	75.3^g^	56.2^g^	0.94	(90)
La_0.7_Sr_0.3_Mn_0.9_Cr_0.1_O_3−δ_	Pechini	3		1350/1200	30^a^/60	84.0	N_2_/N_2_	107.1^g^	160.1^g^	2.67	(127)
La_0.7_Sr_0.3_Mn_0.9_Cr_0.1_O_3−δ_	Pechini	3		1300/1200	30^a^/60	84.0	N_2_/N_2_	92.6^g^	277.8^g^	4.63	(127)
La_0.7_Sr_0.3_Mn_0.9_Cr_0.1_O_3−δ_	Pechini	3		1250/1200	30^a^/60	84.0	N_2_/N_2_	89.7^g^	135.0^g^	2.25	(127)
La_0.7_Sr_0.3_Mn_0.9_Cr_0.1_O_3−δ_	Pechini	3		1200/1200	30^a^/60	84.0	N_2_/N_2_	74.3^g^	82.9^g^	1.38	(127)
La_0.7_Sr_0.3_Mn_0.9_Cr_0.1_O_3−δ_	Pechini	3		1100/1200	30^a^/60	84.0	N_2_/N_2_	29.9^g^	70.4^g^	1.17	(127)
La_0.7_Sr_0.3_Mn_0.9_Cr_0.1_O_3−δ_	Pechini	3		1000/1200	30^a^/60	84.0	N_2_/N_2_	22.2^g^	117.7^g^	1.96	(127)
LaFe_0.95_Mn_0.05_O_3−δ_(25 wt %)/SiO_2_	combustion	1		1350/1100	40/15	100.0	Ar/-		87.1^g^	5.81	(129)
LaFe_0.9_Mn_0.1_O_3−δ_(25 wt %)/SiO_2_	combustion	1		1350/1100	40/15	100.0	Ar/-	117.6^g^	78.3^g^	5.22	(129)
LaFe_0.9_Mn_0.1_O_3−δ_(25 wt %)/SiO_2_	combustion	1		1350/1100	40/60	100.0	Ar/-	117.6^g^	100.4^g^	1.67	(129)
LaFe_0.8_Mn_0.2_O_3−δ_(25 wt %)/SiO_2_	combustion	1		1350/1100	40/15	100.0	Ar/-		67.8^g^	4.52	(129)
LaFe_0.7_Mn_0.3_O_3−δ_(25 wt %)/SiO_2_	combustion	1		1350/1100	40/15	100.0	Ar/-		50.0^g^	3.33	(129)
LaFe_0.6_Mn_0.4_O_3−δ_(25 wt %)/SiO_2_	combustion	1		1350/1100	40/15	100.0	Ar/-		43.8^g^		(129)
LaFe_0.5_Mn_0.5_O_3−δ_(25 wt %)/SiO_2_	combustion	1		1350/1100	40/15	100.0	Ar/-		35.3^g^	2.35	(129)
Sr_0.75_Ce_0.25_MnO_3−δ_	Pechini	3		1400/1000	5.5/20	40.0	Ar/Ar	104.0	224.0	11.20	(76)
Sr_0.75_Ce_0.25_MnO_3−δ_	Pechini	3		1350/850	5.5/20	40.0	Ar/Ar	68.0	98.0	4.90	(76)
La_0.8_Ca_0.2_Mn_0.8_Co_0.2_O_3−δ_	sol–gel	1		1400/800	120/120		Ar/Ar	186.0	256.0	2.13	(128)
La_0.6_Ca_0.4_Mn_0.8_Co_0.2_O_3−δ_	sol–gel	1		1400/800	120/120		Ar/Ar	275.0	88.0	0.73	(128)
La_0.4_Ca_0.6_Mn_0.8_Co_0.2_O_3−δ_	sol–gel	1		1400/800	120/120		Ar/Ar	722.0	6.0	0.05	(128)
La_0.2_Ca_0.8_Mn_0.8_Co_0.2_O_3−δ_	sol–gel	1		1400/800	120/120		Ar/Ar	924.0	27.0	0.23	(128)
La_0.8_Ca_0.2_Mn_0.8_Co_0.2_O_3−δ_	sol–gel	2		1400/800	120/120		Ar/Ar	183.0	82.0	0.68	(128)
La_0.6_Ca_0.4_Mn_0.8_Co_0.2_O_3−δ_	sol–gel	2		1400/800	120/120		Ar/Ar	214.0	78.0	0.65	(128)
La_0.8_Ca_0.2_Mn_0.8_Co_0.2_O_3−δ_	sol–gel	3		1400/800	120/120		Ar/Ar	159.0	44.0	0.37	(128)
La_0.6_Ca_0.4_Mn_0.8_Co_0.2_O_3−δ_	sol–gel	3		1400/800	120/120		Ar/Ar	167.0	54.0	0.45	(128)
Ca_2/3_Ce_1/3_Ti_1/3_Mn_2/3_O_3_	solid-state	1		1350/850	∼30/30	40.0	Ar/Ar		298.8	9.96	(125)
Ca_2/3_Ce_1/3_Ti_1/3_Mn_2/3_O_3_	solid-state	2		1350/850	∼30/30	40.0	Ar/Ar		307.4	10.25	(125)
SrTi_0.5_Mn_0.5_O_3−δ_	solid-state	10		1400/800	30/60	20.0	Ar/Ar	85.3^g^	165.6^g^	2.76	(127)
SrTi_0.5_Mn_0.5_O_3−δ_	solid-state	10		1400/1000	30/60	20.0	Ar/Ar	116.5^g^	237.1^g^	3.95	(127)
SrTi_0.5_Mn_0.5_O_3−δ_	solid-state	10	0.230	1400/1000	30/60	40.0	Ar/Ar	152.7^g^	308.5^g^	5.14	(127)
SrTi_0.5_Mn_0.5_O_3−δ_	solid-state	8		1400/1100	30/60	40.0	Ar/Ar	183.5^g^	371.0^g^	6.18	(127)
SrTi_0.5_Mn_0.5_O_3−δ_	solid-state	10		1400/1200	30/60	40.0	Ar/Ar	134.4^g^	268.3^g^	4.47	(127)
SrTi_0.5_Mn_0.5_O_3−δ_	solid-state	10		1350/1100	30/60	40.0	Ar/Ar	143.8^g^	281.7^g^	4.70	(127)
SrTi_0.5_Mn_0.5_O_3−δ_	solid-state	10		1350/1100	30/60	40.0^!^	Ar/Ar	166.1^g^	330.8^g^	5.51	(127)
SrTi_0.5_Mn_0.5_O_3−δ_	solid-state	10		1350/1100	30/15	40.0	Ar/Ar	54.0^g^	103.6^g^	6.91	(127)
SrTi_0.5_Mn_0.5_O_3−δ_	solid-state	10		1350/1100	30/15	40.0	Ar/Ar	67.9^g^	134.8^g^	8.99	(127)
SrTi_0.5_Mn_0.5_O_3−δ_	solid-state	10		1350/1100	30/15	40.0	Ar/Ar	85.3^g^	170.1^g^	11.34	(127)
CaTi_0.5_Mn_0.5_O_3−δ_	solid-state	14		1350/900	30/60	40.0	Ar/Ar	817.0^g^	228.1^g^	3.80	(66)
CaTi_0.5_Mn_0.5_O_3−δ_	solid-state	14	0.103	1350/1000	30/60	40.0	Ar/Ar	817.0^g^	268.3^g^	4.47	(66)
CaTi_0.5_Mn_0.5_O_3−δ_	solid-state	14		1350/1100	30/60	40.0	Ar/Ar	817.0^g^	295.1^g^	4.92	(66)
CaTi_0.5_Mn_0.5_O_3−δ_	solid-state	14		1350/1150	30/60	40.0	Ar/Ar	817.0^g^	304.0^g^	5.07	(66)
CaTi_0.5_Mn_0.5_O_3−δ_	solid-state	14		1350/1200	30/60	40.0	Ar/Ar	817.0^g^	286.2^g^	4.77	(66)
CaTi_0.5_Mn_0.5_O_3−δ_	solid-state	14		1350/1150	30/60	40.0	Ar/Ar	817.0^g^	339.7^g^	5.66	(66)
CaTi_0.5_Mn_0.5_O_3−δ_	solid-state	14		1350/1150	30/60	40.0	Ar/Ar	817.0^g^	447.3^g^	7.46	(66)
La_0.5_Sr_0.5_Mn_0.9_Mg_0.1_O_3_	Pechini	1		1400/1050	45/60		Ar/-	269.0		0	(131)
La_0.5_Sr_0.5_Mn_0.9_Mg_0.1_O_3_–CeO_2_	suspension	1		1399/1040	40/13.1^h^	17.2	Ar/Ar	87.0	149.0	11.37	(131)
La_0.5_Sr_0.5_Mn_0.9_Mg_0.1_O_3_–CeO_2_	suspension	1		1409/1034	40/6.1^h^	24.2	Ar/Ar	203.0	234.0	38.36	(131)
La_0.5_Sr_0.5_Mn_0.9_Mg_0.1_O_3_–CeO_2_	suspension	1		1407/959	40/7.5^h^	50.0	Ar/Ar	72.0	166.0	22.13	(131)
La_0.5_Sr_0.5_Mn_0.9_Mg_0.1_O_3_	Pechini	1	0.231	1400/1050	-/40	50.0	Ar/Ar	206.0	236.0	5.90	(132)
La_0.5_Sr_0.5_Mn_0.9_Mg_0.1_O_3_	Pechini	2	0.231	1400/1050	-/40	50.0	Ar/Ar	123.0	251.0	6.28	(132)

a Sample was cooled to RT after reduction and pulverized prior to heating to oxidation temperature.

b H_2_O:H_2_ = 1333.

c H_2_O:H_2_ = 1000.

d H_2_O:H_2_ = 750.

e H_2_O:H_2_= 500.

f H_2_O:H_2_ = 285.

g Value converted from mL g_material_^–1^. Standard volume occupied: 22400 mL mol^–1^.

h Amount of time for 90% of fuel to be produced.

Table 6 provides a detailed analysis of the solar thermochemical hydrogen (STCH) production performance of MnX-based perovskite oxides under reducing gas conditions.

**Table 6:** STCH Performance of MnX-Based Perovskite Oxides under Reducing Gas Conditions

composition	synthesis method	cycles	temp. red./ox. (°C)	time red./ox. (min)	H_2_O (%vol)	gas red./ox.	O_2_ yield (μmol g^–1^)	H_2_ yield (μmol g^–1^)	average H_2_ production rate (μmol g^–1^ min^–1^)	ref
La_0.6_Sr_0.4_Mn_0.8_Fe_0.2_O_3−δ_	sol–gel	2	1000/1000	-/20	3.0	N_2_–H_2_/N_2_^a^	286.0^b^	1205.0^c^	60.25	(96)
La_0.6_Sr_0.4_Mn_0.6_Fe_0.4_O_3−δ_	sol–gel	2	1000/1000	-/20	3.0	N_2_–H_2_/N_2_^a^	280.0^b^	1645.0^c^	82.25	(96)
La_0.6_Sr_0.4_Mn_0.4_Fe_0.6_O_3−δ_	sol–gel	2	1000/1000	-/20	3.0	N_2_–H_2_/N_2_^a^	333.2^b^	2827.0^c^	141.35	(96)

a 5 vol % H_2_ stream.

b Results from TGA analysis.

c Introduction of steam began at 60 °C while ramping to 1000 °C

### 5.4 Fe- and FeX-Based Perovskite Oxides As Redox-Active Oxygen Exchange Materials

Alongside La_1–*x*_Sr_*x*_MnO_3_ perovskites, La_1–*x*_Sr_*x*_FeO_3_ was also among the first families of perovskites tested for STCH. When compared to the temperature requirements for the reduction of manganese-based materials (LSM-20 and LSM-35), which necessitate temperatures close to 1400 °C, the theoretical temperature needed for the reduction of Fe-based materials is lower, at 1150 °C.^97^ In 2009, Nalbandian et al. followed the same parameters and synthesis used by Edvou et al. using the oxidation of CH_4_ as a means of reducing their perovskite material and pure steam as the oxidizing medium. Their perovskites achieved cumulative H_2_ production of 261.0 μmol g^–1^ per ∼2000 μmol g^–1^ of H_2_O at *x* = 0.3, while reaching 123.0 μmol g^–1^ of H_2_ per ∼1000 μmol g^–1^ of H_2_O at *x* = 0.7 and 130.0 μmol g^–1^ of H_2_ per ∼1000 μmol g^–1^ of H_2_ at *x* = 1.^133^ Commercial La_0.6_Sr_0.4_FeO_3−δ_ rendered 124.0 μmol g^–1^ of H_2_ under isothermal 1400 °C conditions with undefined reduction/oxidation times, and undefined steam concentration.^97^ G. Luciani et al. examined the perovskite La_0.6_Sr_0.4_FeO_3−δ_, which exhibited the most substantial release of oxygen (427.3 μmol g^–1^) during thermogravimetric analysis and generated significant hydrogen production (3359.0 μmol g^–1^) in splitting tests.^96^ In 2014, the commercial La_0.6_Sr_0.4_FeO_3−δ_, which was also used as a mixed ionic electronic conductor in fuel cells, was also investigated by Stéphane Abanades’s group, who found an oxygen production of 425 μmol g^–1^ at 1200 °C.^95^ Additionally, Lulu Wang et al. similarly explored La_0.6_Sr_0.4_FeO_3−δ_, emphasizing the essential role of oxygen vacancies within metal oxide for controlling significant redox properties.^105^ LaFeO_3_ has recently attracted attention for its impressive performance in photocatalytic hydrogen production, but its use in STCH has been relatively unexplored. In this study, Jin et al. employed a first-principles method at the density functional theory level to investigate the hydrogen production mechanism of LaFeO_3_ perovskite doped with 25% Sr/Ca at the A-site. The rate of hydrogen production is determined by the migration of hydrogen at the surface. The water-splitting (WS) mechanism of La_0.75_A_0.25_FeO_3_ (A = Ca, Sr) elucidates the process of water-splitting and H_2_ generation on the defected material surface. For example, in the case of La_0.75_Sr_0.25_FeO_3_, the process begins with the approach and adsorption of H_2_O on the defective surface, releasing 16.94 kJ mol^–1^ of energy. Subsequently, one of the two H–O bonds of H_2_O is broken, and the H atom combines with an adjacent lattice O atom on the surface, forming a hydroxyl group. Another hydroxyl group fills the oxygen vacancy, releasing 43.72 kJ mol^–1^ of energy. This decomposition of H_2_O into two hydroxyl groups is followed by successive hydrogen production and desorption steps. In summary, LaFeO_3_’s hydrogen production mechanism involves several energy-releasing steps, with a distinct transition state observed during hydrogen migration.^134^ Recently, Park et al. have introduced another mechanism in which the distribution of the oxygen formation energy (therefore enthalpy) can lead to increased H_2_ production, particularly when the *P*_H_2__/*P*_H_2_O_ ratio is relatively low. Their results indicate that distribution refers to both the average and the spreading of the oxygen vacancy formation energy.^135^ Maria Batuk et al. were the first to report in situ 3D electron diffraction (ED) studies of SrFeO_2.5_ in both O_2_ and H_2_ environments. Their research provided detailed insights into the structural changes occurring during redox reactions at the nanoscale, involving individual particles with dimensions of a few hundred nanometers. The results notably revealed the formation of the SrFeO_2.75_*Cmmm* structure upon oxidation, followed by a subsequent transformation into the perovskite SrFeO_3−δ_ with a *Pm*3̅*m* structure. Importantly, this phase transition had not been observed in prior in situ experiments conducted in gas environments using X-ray or neutron powder diffraction techniques.^136^

**Figure 12:**
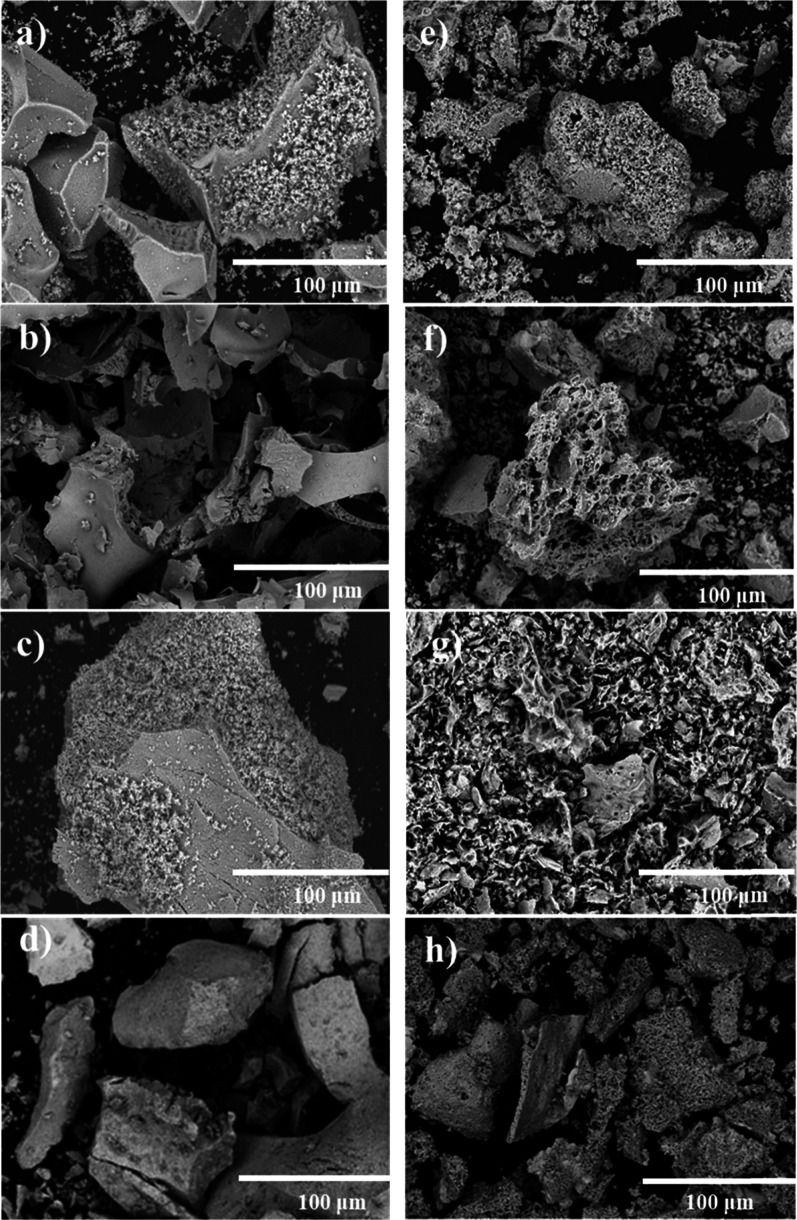
SEM imagines of (a) La_0.8_Al_0.2_CoO_3−δ_ (acid), (b) La_0.8_Al_0.2_NiO_3−δ_ (acid), (c) La_0.8_Al_0.2_FeO_3−δ_ (acid), (d) La_0.8_Al_0.2_CuO_3−δ_ (acid), (e) La_0.8_Al_0.2_CoO_3−δ_ (basic), (f) La_0.8_Al_0.2_NiO_3−δ_ (basic), (g) La_0.8_Al_0.2_FeO_3−δ_ (basic), and (h) La_0.8_Al_0.2_CuO_3−δ_ (basic).^138^ Reproduced with permission from ref (138). Copyright 2022 Elsevier.

In addition to the presence of Sr on the A-site, Hartley’s group also investigated the incorporation of Ba on the A-site. They discovered that Ba_0.95_La_0.05_FeO_3−δ_ (BLF) with a cubic phase exhibited the most substantial H_2_ production (approximately 1310 μmol g^–1^) under an isothermal reduction/oxidation temperature of 900 °C. However, it should be noted that this catalyst faced challenges related to “sintering” (coarsening of nanoparticles).^137^ Alejandro Perez et al. studied the hydrogen production by thermochemical H_2_O splitting with a La_0.8_Al_0.2_FeO_3−δ_ perovskite prepared under a controlled pH during the synthesis process. The reason for changing the pH is that, in the Pechini method, the pH level significantly affects the stability and homogeneity of metal citrate solutions.^138^Figure 12 displays the SEM images of a series of perovskites synthesized at various pH levels, demonstrating their significant impact on the morphological attributes of the perovskites.

Mingkai Fu et al. first filled the gap in the water dissociative mechanism of LaFeO_3_. They meticulously calculated the entire pathway for water-splitting and H_2_ production on a defective LaFeO_3_ (010) surface. Their findings reveal a distinctive characteristic of the oxidation step on the defected LaFeO_3_ surface, which is endothermic in nature. This differs markedly from the exothermic water-splitting behavior observed in many other redox materials like CeO_2_ and SnO_2_.^139^

In addition to the extensively researched Fe-based perovskites, the exploration of FeX-based materials (where X includes Gd, Co, Ni, Al, Ir, and Ti as the doping elements on the B site) has also captured the attention of researchers. For example, when subjecting the materials to TGA testing, the presence of cobalt leads to a more substantial reduction in weight when comparing La_0.6_Sr_0.4_Co_0.2_Fe_0.8_O_3−δ_ to La_0.6_Sr_0.4_FeO_3−δ_. This aligns with the general trend of cobalt oxides exhibiting greater ease of reduction compared to iron oxides at equivalent oxidation states.^95^ A series of Ba_*x*_Sr_1–*x*_Co_*y*_Fe_1–*y*_O_3−δ_ (*x* = 0.5, 0.25; y = 0.2, 0.4, 0.6, 0.8) materials were also explored by Stéphane Abanades’s group, and they mentioned that introducing Sr^2+^ as a substitute for Ba^2+^ within the BSCF series could potentially stimulate the formation of M^4+^ (M = Mn, Co, Fe) cations, consequently enhancing the likelihood of achieving elevated reduction yields.^95^ The combination of Fe and Co in the B-site of the perovskite is beneficial for fuel products. Here, Can Li’s group screened a series LaFe_1–*y*_Co_*y*_O_3_ (y = 0, 0.1, 0.2, 0.3, 0.4, and 0.5) and found that when *y* = 0.2 the H_2_ and O_2_ production is the highest and the kinetic is the fast as the result of H_2_ production of the initial 15 min.^129^ Moreover, Zhenpan Chen et al. investigated the effects of a SiO_2_ support on the LaMn_*y*_Fe_1–*y*_O_3_ perovskite, and they found SiO_2_ support was helpful to mitigate sintering effects and disperse iron silicon oxides at high temperatures. Their approach involved the usage of modified glycine nitrate autocombustion to synthesize their perovskites, which were subsequently mechanically milled with SiO_2_ prior to the STCH analysis. A temperature sweep of 250 K (1350/1100 °C) was employed, with a reduction time of 40 min, an oxidation time of 15 or 60 min, and pure steam as the oxidizing gas. They witnessed the enhancement of H_2_ in LaFeO_3_ through the support of 25 wt % SiO_2_, advancing from a negligible H_2_ yield to reach 96.9 μmol g^–1^ after 15 min and a total of 130.5 μmol g^–1^ after the sample was fully oxidized. Doping with Mn did not result in an increase in the STCH performance, being rather detrimental to the yield of H_2_. Furthermore, Chen et al. briefly studied the effects of Ni or Al doping in LaFeO_3_ (25 wt %)/SiO_2_ at 10% doping concentration. They too did not see an increase to the production of H_2_, with LaFe_0.9_Ni_0.1_O_3_ (25 wt %)/SiO_2_ producing 1.4 μmol g^–1^ more than LaFeO_3_ (25 wt %)/SiO_2_ when fully oxidized.^129^ The previous study from their group showed the IrO_*x*_-catalyzed CO_2_ at high temperatures with similar catalytic functions compared to the catalysis of the chemical reaction at the gas–solid interface is probably facilitated by iridium dopants present at the exposed microscale surface, which aligns with the reaction mechanism governed by surface control.^140^ Similarly, they found even that when the doping level was a mere 1 at. %, the oxygen release is increased. Additionally, the maximum H_2_ rate enhanced roughly 0.5× when 1 at. % Ir was added to LaFe_0.99_Ir_0.01_O_3_/SiO_2_.^129^ To investigate perovskite materials that can be formed from more earth-abundant elements and to establish techniques for the efficient synthesis of a diverse range of potential compositions, the CaTi_*x*_Fe_1–*x*_O_3_ (*x* = 0.7, 0.8, 0.9, 1) series was studied, and researchers found these oxides exhibit water-splitting activity. Additionally, they reported the redox behavior of CaTi_0.7_Fe_0.3_O_3_ is similar to that of CeO_2_, and the total quantity of oxygen produced by CaTi_0.7_Fe_0.3_O_3_ when the reduction temperature at 1320 °C is similar to CeO_2_.^77^ Sha Chen and colleagues conducted a study using density functional theory calculations to explore how water interacts with the surfaces of perovskite materials based on SrFeO_3−δ_, doped with different metals (B = Al, Zr, Nb, and W). Their findings revealed that Zr-, Nb-, and W-doped structures enhanced the production of H_2_, while the Al-doped structure did not facilitate H_2_ release. The calculations demonstrated that the introduction of metal dopants had a positive effect on the molecular and dissociative adsorption of H_2_O on both perfect and oxygen-deficient surfaces. Furthermore, the presence of surface oxygen vacancies was predicted to promote the dissociative adsorption of H_2_O and the subsequent formation of H_2_.^141^Table 7 indicates the STCH performance of Fe and FeX-based perovskite oxides under inert gas conditions.

**Table 7:** STCH Performance of Fe- and FeX-Based Perovskite Oxides under Inert Gas Conditions

composition	synthesis method	cycles	Δδ	temp. red./ox. (°C)	time red./ox. (min)	H_2_O (% vol)	gas red./ox.	O_2_ yield (μmol g^–1^)	H_2_ yield (μmol g^–1^)	average H_2_ production rate (μmol g^–1^ min^–1^)	ref
La_0.6_Sr_0.4_FeO_3−δ_	commercial	5	0.030	1400/1400			N_2_/N_2_	327.2	124.0		(97)
La_0.6_Sr_0.4_FeO_3−δ_	commercial	1		1200/-	45/-		Ar/-	425.0			(95)
La_0.6_Sr_0.4_FeO_3−δ_	Pechini	1	0.170	1300/900	60/60	40.0	Ar/Ar	375.0	349.0	5.82	(105)
BaFeO_3−δ_	sol–gel	9		700/700	30/30	30.0	Ar/Ar		∼216.85		(137)
Ba_0.95_La_0.05_FeO_3−δ_	sol–gel	9		500/500	30/30	30.0	Ar/Ar		∼48.9		(137)
Ba_0.95_La_0.05_FeO_3−δ_	sol–gel	9		900/900	30/30	30.0	Ar/Ar		∼1485.5		(137)
BaFe_0.975_Gd_0.025_O_3−δ_	sol–gel	9		700/700	30/30	30.0	Ar/Ar		∼297.56		(137)
La_0.8_Al_0.2_FeO_3−δ_-acid pH	Pechini	1		1400/800	80/80	100.0	N_2_/N_2_		271.88^a^	3.40	(138)
La_0.8_Al_0.2_FeO_3−δ_-acid pH	Pechini	1		1200/800	80/80	100.0	N_2_/N_2_		19.53^a^	0.24	(138)
La_0.8_Al_0.2_FeO_3−δ_-acid pH	Pechini	1		1000/800	80/80	100.0	N_2_/N_2_		315.18^a^	3.94	(138)
La_0.8_Al_0.2_FeO_3−δ_-acid pH	Pechini	1		800/800	80/80	100.0	N_2_/N_2_		131.14^a^	1.64	(138)
La_0.8_Al_0.2_FeO_3−δ_-basic pH	Pechini	1		1400/800	80/80	100.0	N_2_/N_2_		19.53^a^	0.24	(138)
La_0.8_Al_0.2_FeO_3−δ_-basic pH	Pechini	1		1200/800	80/80	100.0	N_2_/N_2_		244.2^a^	3.05	(138)
La_0.8_Al_0.2_FeO_3−δ_-basic pH	Pechini	1		1000/800	80/80	100.0	N_2_/N_2_		308.04^a^	3.85	(138)
La_0.8_Al_0.2_FeO_3−δ_-basic pH	Pechini	1		800/800	80/80	100.0	N_2_/N_2_		131.14^a^	1.64	(138)
Ba_0.5_Sr_0.5_Co_0.2_Fe_0.8_O_3−δ_	solid-state	1		1000/-	30/-		Ar/-	630.0			(95)
Ba_0.5_Sr_0.5_Co_0.6_Fe_0.4_O_3−δ_	solid-state	1		1000/-	30/-		Ar/-	611.0			(95)
Ba_0.5_Sr_0.5_Co_0.8_Fe_0.2_O_3−δ_	Pechini	1		1000/800	30/45	80.0	Ar/Ar	600.0	102.0	2.27	(95)
Ba_0.5_Sr_0.5_Co_0.8_Fe_0.2_O_3−δ_	solid-state	1		1000/800	30/45	80.0	Ar/Ar	600.0	83.0	1.84	(95)
Ba_0.25_Sr_0.75_Co_0.8_Fe_0.2_O_3−δ_	solid-state	2		1000/800	30/45	80.0	Ar/Ar	700.0	74.0	1.64	(95)
La_0.8_Sr_0.2_FeO_3−δ_(25 wt %)/SiO_2_	combustion	1		1350/1100	40/60	100.0	Ar/-	105.0^a^	126.2^a^	2.10	(129)
La_0.8_Ba_0.2_FeO_3−δ_(25 wt %)/SiO_2_	combustion	1		1350/1100	40/60	100.0	Ar/-	100.6^a^	126.6^a^	2.11	(129)
La_0.8_Ce_0.2_FeO_3−δ_(25 wt %)/SiO_2_	combustion	1		1350/1100	40/60	100.0	Ar/-	80.9^a^	85.9^a^	1.43	(129)
LaFeO_3−δ_(25 wt %)/SiO_2_	combustion	1		1350/1100	40/15	100.0	Ar/-	88.3^a^	96.9^a^	6.46	(129)
LaFeO_3−δ_(25 wt %)/SiO_2_	combustion	1		1350/1100	40/60	100.0	Ar/-	88.3^a^	130.5^a^	2.175	(129)
LaFe_0.95_Mn_0.05_O_3−δ_(25 wt %)/SiO_2_	combustion	1		1350/1100	40/15	100.0	Ar/-		87.1^a^	5.81	(129)
LaFe_0.9_Mn_0.1_O_3−δ_(25 wt %)/SiO_2_	combustion	1		1350/1100	40/15	100.0	Ar/-	117.6^a^	78.3^a^	5.22	(129)
LaFe_0.9_Mn_0.1_O_3−δ_(25 wt %)/SiO_2_	combustion	1		1350/1100	40/60	100.0	Ar/-	117.6^a^	100.4^a^	1.67	(129)
LaFe_0.9_Ni_0.1_O_3−δ_(25 wt %)/SiO_2_	combustion	1		1350/1100	40/60	100.0	Ar/-	93.3^a^	131.9^a^	2.20	(129)
LaFe_0.9_Al_0.1_O_3−δ_(25 wt %)/SiO_2_	combustion	1		1350/1100	40/60	100.0	Ar/-	118.6^a^	115.2^a^	1.92	(129)
LaFe_0.8_Mn_0.2_O_3−δ_(25 wt %)/SiO_2_	combustion	1		1350/1100	40/15	100.0	Ar/-		67.8^a^	4.52	(129)
LaFe_0.7_Mn_0.3_O_3−δ_(25 wt %)/SiO_2_	combustion	1		1350/1100	40/15	100.0	Ar/-		50.0^a^	3.33	(129)
LaFe_0.6_Mn_0.4_O_3−δ_(25 wt %)/SiO_2_	combustion	1		1350/1100	40/15	100.0	Ar/-		43.8^a^	2.92	(129)
LaFe_0.5_Mn_0.5_O_3−δ_(25 wt %)/SiO_2_	combustion	1		1350/1100	40/15	100.0	Ar/-		35.3^a^	2.35	(129)
CaTi_0.7_Fe_0.3_O_3_	solid-state			1400/1100	-/10	40.0	He/-		39.0	3.9	(77)

a Value converted from mL g_material_^–1^. Standard volume occupied: 22400 mL mol^–1^.

Table 8 presents a comprehensive analysis of the solar thermochemical hydrogen (STCH) production performance of Fe- and FeX-based perovskite oxides under reducing gas conditions.

**Table 8:** Fe and FeX-based perovskite oxides STCH performance under reducing gas conditions

composition	synthesis method	cycles	temp. red./ox. (°C)	time red/.ox. (min)	H_2_O (% vol)	gas red./ox.	O_2_ yield (μmol g^–1^)	H_2_ yield (μmol g^–1^)	average H_2_ production rate (μmol g^–1^ min^–1^)	ref
La_0.7_Sr_0.3_FeO_3−δ_	sol–gel	3	1000/1000		100.0	He-CH_4_/-		261.0		(133)
La_0.3_Sr_0.7_FeO_3−δ_	sol–gel	3	1000/1000		100.0	He-CH_4_/-		123.0		(133)
SrFeO_3−δ_	sol–gel	3	1000/1000		100.0	He-CH_4_/-	270.0	130.0		(133)
LaFeO_3−δ_	sol–gel	3	1000/-			He-CH_4_/-	530.0			(133)
La_0.6_Sr_0.4_FeO_3−δ_	sol–gel	2	1000/1000	-/20	3.0	aN_2_–H_2_/N_2_	427.3^b^	3359.0^c^	167.95	(96)
La_0.6_Sr_0.4_Mn_0.8_Fe_0.2_O_3−δ_	sol–gel	2	1000/1000	-/20	3.0	aN_2_–H_2_/N_2_	286.0^b^	1205.0^c^	60.25	(96)
La_0.6_Sr_0.4_Mn_0.6_Fe_0.4_O_3−δ_	sol–gel	2	1000/1000	-/20	3.0	aN_2_–H_2_/N_2_	280.0^b^	1645.0^c^	82.25	(96)
La_0.6_Sr_0.4_Mn_0.4_Fe_0.6_O_3−δ_	sol–gel	2	1000/1000	-/20	3.0	aN_2_–H_2_/N_2_	333.2^b^	2827.0^c^	141.35	(96)
BaFeO_3−δ_	sol–gel	9	500/500	30/30	30.0	10%H_2_–Ar/Ar		∼26.43		(137)
BaFeO_3−δ_	sol–gel	9	900/900	30/30	30.0	10%H_2_–Ar/Ar		∼1285.8		(137)
Ba_0.95_La_0.05_FeO_3−δ_	sol–gel	9	700/700	30/30	30.0	10%H_2_–Ar/Ar		∼370.2		(137)
BaFe_0.975_Gd_0.025_O_3−δ_	sol–gel	9	500/500	30/30	30.0	10%H_2_–Ar/Ar		∼42.84		(137)
BaFe_0.975_Gd_0.025_O_3−δ_	sol–gel	9	900/900	30/30	30.0	10%H_2_–Ar/Ar		∼737.28		(137)

a 5 vol % H_2_ stream.

b Results from TGA analysis.

c Introduction of steam began at 60 °C while ramping to 1000 °C

### 5.5 Co- and CoX-Based Perovskite Oxides as Redox-Active Oxygen Exchange Materials

**Figure 13:**
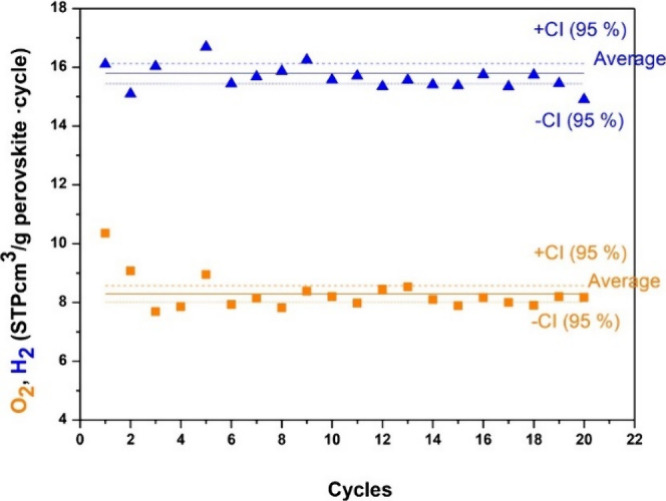
Performance of LSC materials in 20 thermochemical cycles. This involves assessing oxygen release at 1000 °C and hydrogen release at 800 °C.^143^ Reproduced from ref (143). Available under a Creative Commons CC-BY license. Copyright 2022 M. Orfila and co-workers.

The lanthanum–cobalt perovskites have been investigated in thermochemical cycles by various research groups. For instance, Stéphane Abanades’s group reported that La_0.8_Sr_0.2_CoO_3−δ_ produces 732 μmol g^–1^ of oxygen at 1200 °C.^95^ Additionally, Mara Orfila et al. evaluated a commercially available Co-based perovskite material, La_0.8_Sr_0.2_CoO_3−δ_. The study revealed that La_0.8_Sr_0.2_CoO_3−δ_ exhibited the highest activity compared to iron-based material La_0.6_Sr_0.4_FeO_3−δ_ and manganese materials (LSM-20, LSM-35), as observed through thermogravimetric analysis. Furthermore, theoretical reduction temperatures for Co-based materials were found to be lower at 1050 °C in comparison to those of Mn-based and Fe-based perovskite. However, both H_2_-TPR and SEM results confirmed that the cobalt material undergoes significant changes after 4 reaction cycles, negatively impacting hydrogen production.^97^ Lulu Wang et al. investigated a series of La_1–*x*_Ca_*x*_CoO_3_ perovskites for two-step thermochemical H_2_O splitting cycles, revealing the significant impact of the Ca dopant content on both thermal reduction and H_2_O splitting steps. Enhanced O_2_ evolution was achieved with increased Ca doping, but this compromised H_2_O splitting thermodynamics and led to lower reoxidation yields. Among the compositions, La_0.6_Ca_0.4_CoO_3_ exhibited the best performance, with the optimal thermochemical operational conditions identified between 1300 (thermal reduction) and 900 °C (reoxidation), resulting in a notable H_2_ yield of 587 μmol g^–1^.^142^ Co-based perovskite oxides were extensively investigated in terms of the extent of reduction (δ). For instance, Wang et al. also reported significant H_2_ (514 μmol g^–1^) and O_2_ (718 μmol g^–1^) production from the La_0.6_Sr_0.4_CoO_3_ perovskite under a temperature combination of 1300/900 °C. Furthermore, experimental observations have indicated that La_0.6_Sr_0.4_CoO_3_ exhibits the highest formation of oxygen vacancies on the B-site when compared to Ni, Fe, Cr, and Mn. This indicates its potential as an active catalyst for H_2_O splitting. Additionally, La_0.6_Sr_0.4_CoO_3_ demonstrates the highest weight loss measured by TGA. However, after the cycle reaction, La_0.6_Sr_0.4_CoO_3_ exhibits significant particle aggregation and a phase change.^105^ La_0.8_Al_0.2_CoO_3−δ_ was synthesized using the Pechini method at various pH levels, as studied by Pérez et al. The research revealed that both the thermal reduction and hydrolysis stages induce permanent structural alterations when synthesized under acidic pH conditions. However, such changes are not observed when the synthesis takes place under basic pH conditions and the material is processed at 800 °C.^138^ La_0.8_Sr_0.2_CoO_3_ (LSC), with higher exergy (36%) and solar to fuel efficiencies (67%) according to the thermodynamic evaluation, stands out as a promising material in comparison to other reported materials. Moreover, LSC has stable hydrogen production during 20 consecutive cycles at 1000 °C, as shown in Figure 13; however, at a high reduction temperature (>1000 °C), the formation of the segregated Co_3_O_4_ phase is induced, resulting in a decrease in hydrogen production.^143^

Lanthanum–cobalt perovskites substituted in the A- or/and B-site were investigated. The cubic structure perovskite Sr_2_CoNb_1–*x*_Ti_*x*_O_6−δ_ (*x* = 1.00, 0.70) exhibiting outstanding H_2_ performance even at 700 °C were investigated by Prof. M. Teresa Azcondo et al. They also found a Sr_2_CoNb_0.3_Ti_0.7_O_6−δ_ material that maintained its remarkable performance upon cycling. The Mn perovskite undergoes thermal reduction at temperatures exceeding 1000 °C, while the Co material requires 800 °C and the Fe material requires 900 °C. Additionally, after eight thermochemical reactions at 700 °C, Sr_2_CoNb_0.30_Ti_0.70_O_6−δ_ showed the highest ever reported hydrogen production of 410 μmol g^–1^.^144^ Perovskite La_0.6_Sr_0.4_Cr_1–*x*_Co_*x*_O_3_ used in water-splitting was first reported by A. H. Bork et al. They exhibit that there is a trade-off in doping: the introduction of cobalt dopants enhances the liberation of oxygen during the initial high-temperature reduction phase; however, this advantage is counterbalanced by the less advantageous thermodynamics observed during the subsequent second step involving water-splitting at lower temperatures.^145^ Sr- and Co-doped LaGaO_3−δ_ was successfully investigated in thermochemical water-splitting (TCWS) by Can Li’s group. They found that the oxygen vacancy concentration (δ) increased with the content of the Sr and Co dopants. The catalyst IrO_*x*_, doped into the LGC50 structure, was found to increase the hydrogen release rate by 1.3-fold. XPS analysis revealed that the water-splitting reaction was driven by Co^2+^/Co^3+^ ions. The oxygen vacancy formation energies (Ev) were predicted using DFT calculations, which found that *E*_v_ is from about 0.7 to nearly 5.3 eV at 1623 K under vacuum conditions. However, under the evaluation conditions, the low water-to-hydrogen conversion (less than 1%) is still far away from the practical needs.^119^ In a study by Park et al., the perovskite Gd_0.5_La_0.5_Co_0.5_Fe_0.5_O_3_ was identified as a promising water-splitting catalyst through computational analysis, and its efficacy was subsequently confirmed experimentally. In water-splitting assessments conducted at a thermal reduction temperature of 1350 °C, the reoxidation temperatures of 850 and 1000 °C yielded hydrogen outputs of 101 and 141 μmol g^–1^, respectively, with a progressive enhancement in production across successive cycles.^146^Table 9 provides a detailed overview of the solar thermochemical hydrogen (STCH) production performance of Co- and CoX-based perovskite oxides.

**Table 9:** STCH Performance of Co- and CoX-Based Perovskite Oxides

composition	synthesis method	cycles	Δδ	temp. red./ox. (°C)	time red./ox. (min)	H_2_O (%vol)	gas red./ox.	O_2_ yield (μmol g^–1^)	H_2_ yield (μmol g^–1^)	average H_2_ production rate (μmol g^–1^ min^–1^)	ref
La_0.8_Sr_0.2_CoO_3−δ_	commercial	1		1200/-	45/-		Ar/-	732.0			(95)
La_0.6_Sr_0.4_Fe_0.8_Co_0.2_O_3−δ_	commercial	2		1200/800	45/45	80.0	Ar/Ar	503.0	90.0	2.00	(95)
La_0.6_Sr_0.4_Fe_0.8_Co_0.2_O_3−δ_	Pechini	2		1200/800	45/45	80.0	Ar/Ar	503.0	162.0	3.60	(95)
La_0.8_Sr_0.2_CoO_3−δ_	commercial	5	0.220	1400/1400			N_2_/N_2_	492.7	272.8		(97)
La_0.8_Ca_0.2_CoO_3−δ_	Pechini	1		1300/900	60/60	40.0	Ar/Ar	715.0	496.0	8.27	(142)
La_0.6_Ca_0.4_CoO_3−δ_	Pechini	5		1300/900	60/60	40.0	Ar/Ar	832.5	587.0	9.78	(142)
La_0.4_Ca_0.6_CoO_3−δ_	Pechini	1		1300/900	60/60	40.0	Ar/Ar	157.5	472.0	7.87	(142)
La_0.2_Ca_0.8_CoO_3−δ_	Pechini	1		1300/900	60/60	40.0	Ar/Ar	1213.0	204.0	3.40	(142)
La_0.6_Sr_0.4_CoO_3−δ_	Pechini	5	0.330	1300/900	60/60	40.0	Ar/Ar	718.0	514.0	8.57	(105)
La_0.6_Sr_0.4_CrO_3−δ_	Pechini	1	0.072	1300/900	60/60	40.0	Ar/Ar	166.0	280.0	4.67	(105)
La_0.6_Sr_0.4_NiO_3−δ_	Pechini	1	0.200	1300/900	60/60	40.0	Ar/Ar	437.0	368.0	6.13	(105)
La_0.8_Al_0.2_CoO_3−δ_-acid pH	Pechini	1		1400/800	80/80	100.0	N_2_/N_2_		503.57^a^	6.29	(138)
La_0.8_Al_0.2_CoO_3−δ_-acid pH	Pechini	1		1200/800	80/80	100.0	N_2_/N_2_		239.73^a^	3.00	(138)
La_0.8_Al_0.2_CoO_3−δ_-acid pH	Pechini	1		1000/800	80/80	100.0	N_2_/N_2_		16.74^a^	0.21	(138)
La_0.8_Al_0.2_CoO_3−δ_-acid pH	Pechini	1		800/800	80/80	100.0	N_2_/N_2_		136.72^a^	1.71	(138)
La_0.8_Al_0.2_CoO_3−δ_-basic pH	Pechini	1		1400/800	80/80	100.0	N_2_/N_2_		366.96^a^	4.59	(138)
La_0.8_Al_0.2_CoO_3−δ_-basic pH	Pechini	1		1200/800	80/80	100.0	N_2_/N_2_		213.39^a^	2.67	(138)
La_0.8_Al_0.2_CoO_3−δ_-basic pH	Pechini	1		1000/800	80/80	100.0	N_2_/N_2_		309.71^a^	3.87	(138)
La_0.8_Al_0.2_CoO_3−δ_-basic pH	Pechini	1		800/800	80/80	100.0	N_2_/N_2_		133.93^a^	1.67	(138)
La_0.8_Sr_0.2_CoO_3−δ_	commercial	1		1400/800		100.0	N_2_/N_2_	915.18^a^	296.34^a^		(143)
La_0.8_Sr_0.2_CoO_3−δ_	commercial	1		1200/800		100.0	N_2_/N_2_	618.3^a^	279.02^a^		(143)
La_0.8_Sr_0.2_CoO_3−δ_	commercial	1		1000/800		100.0	N_2_/N_2_	461.16^a^	717.41^a^		(143)
La_0.8_Sr_0.2_CoO_3−δ_	commercial	1		800/800		100.0	N_2_/N_2_	11.01^a^	3.67^a^		(143)
La_0.8_Sr_0.2_CoO_3−δ_	commercial	20		1000/800		100.0	N_2_/N_2_	364.73^a^	665.18^a^		(143)
Sr_2_CoNb_0.3_Ti_0.7_O_6−δ_	Pechini	1		700/700		100.0	N_2_/N_2_	235.29	450.0		(144)
Sr_2_CoNb_0.3_Ti_0.7_O_6−δ_	Pechini	8		700/700		100.0	N_2_/N_2_	200.78	410.0		(144)
Sr_2_CoTiO_6−δ_	Pechini	1		700/700		100.0	N_2_/N_2_	260.68	492.0		(144)
Sr_2_CoTiO_6−δ_	Pechini	8		700/700		100.0	N_2_/N_2_	181.55	238.18		(144)
LaGaO_3−δ_	combustion	1	0.010	1350/650	40/-	100.0	Ar/-	2.0	6.0		(119)
LaGa_0.99_Co_0.01_O_3−δ_	combustion	1	0.010	1350/650	40/-	100.0	Ar/-	26.0	8.0		(119)
LaGa_0.9_Co_0.1_O_3−δ_	combustion	1	0.044	1350/650	40/-	100.0	Ar/-	75.0	146.0		(119)
LaGa_0.85_Co_0.15_O_3−δ_	combustion	1		1350/650	40/-	100.0	Ar/-	90.0	128.0		(119)
LaGa_0.8_Co_0.2_O_3−δ_	combustion	1	0.056	1350/650	40/-	100.0	Ar/-	116.0	185.0		(119)
LaGa_0.7_Co_0.3_O_3−δ_	combustion	1	0.084	1350/650	40/-	100.0	Ar/-	172.0	254.0		(119)
LaGa_0.6_Co_0.4_O_3−δ_	combustion	1	0.106	1350/650	40/-	100.0	Ar/-	203.0	329.0		(119)
LaGa_0.5_Co_0.5_O_3−δ_	combustion	1	0.137	1350/550	40/-	100.0	Ar/-	227.0	258.0		(119)
LaGa_0.5_Co_0.5_O_3−δ_	combustion	1	0.137	1350/650	40/-	100.0	Ar/-	268.0	406.0		(119)
LaGa_0.5_Co_0.5_O_3−δ_	combustion	1	0.137	1350/700	40/-	100.0	Ar/-	260.0	440.0		(119)
LaGa_0.5_Co_0.5_O_3−δ_	combustion	1	0.137	1350/800	40/-	100.0	Ar/-	272.0	450.0		(119)
LaGa_0.5_Co_0.5_O_3−δ_	combustion	1	0.137	1350/900	40/-	100.0	Ar/-	256.0	440.0		(119)
LaGa_0.5_Co_0.5_O_3−δ_	combustion	1	0.137	1350/1000	40/-	100.0	Ar/-	246.0	328.0		(119)
LaGa_0.5_Co_0.5_O_3−δ_	combustion	1	0.137	1350/1100	40/-	100.0	Ar/-	202.0	301.0		(119)
LaGa_0.5_Co_0.5_°_3−δ_-Ir(0.5 atom %)	combustion	1		1350/800	40/100	100.0	Ar/-	268.0	363.0	3.63	(119)
LaGa_0.5_Co_0.5_O_3−δ_-Ir(1.0 atom %)	combustion	1	0.131	1350/800	40/100	100.0	Ar/-	274.0	413.0	4.13	(119)
LaGa_0.5_Co_0.5_O_3−δ_-Ir(1.5 atom %)	combustion	1		1350/800	40/100	100.0	Ar/-	274.0	337.0	3.37	(119)
LaGa_0.4_Co_0.6_O_3−δ_	combustion	3	0.156	1350/800	40/400	100.0	Ar/-	313.0	478.0	1.20	(119)
LaGa_0.3_Co_0.7_O_3−δ_	combustion	1	0.160	1350/800	40/530	100.0	Ar/-	321.0	196.0	0.37	(119)
LaCoO_3−δ_	combustion	1	0.245	1350/800	40/250	100.0	Ar/-	500.0	69.0	0.28	(119)
La_0.9_Sr_0.1_GaO_3−δ_	combustion	1	0.137	1350/800	40/-	100.0	Ar/-	4.0	4.0		(119)
La_0.9_Sr_0.1_Ga_0.5_Co_0.5_O_3−δ_	combustion	1	0.160	1350/800	40/-	100.0	Ar/-	328.0	215.0		(119)
La_0.7_Sr_0.3_Ga_0.5_Co_0.5_O_3−δ_	combustion	1	0.167	1350/1000	40/-	100.0	Ar/-	355.0	45.0		(119)
La_0.5_Sr_0.5_Ga_0.5_Co_0.5_O_3−δ_	combustion	1		1350/1000	40/-	100.0	Ar/-	379.0	4.0		(119)
Gd_0.5_La_0.5_Co_0.5_Fe_0.5_O_3_	solid-state	1		1350/850	5.5/20	40.0	Ar/Ar	261.0	67.0	3.35	(146)
Gd_0.5_La_0.5_Co_0.5_Fe_0.5_O_3_	solid-state	2		1350/850	5.5/20	40.0	Ar/Ar	87.0	90.0	4.50	(146)
Gd_0.5_La_0.5_Co_0.5_Fe_0.5_O_3_	solid-state	3		1350/850	5.5/20	40.0	Ar/Ar	74.0	101.0	5.05	(146)
Gd_0.5_La_0.5_Co_0.5_Fe_0.5_O_3_	solid-state	4		1350/850	5.5/20	40.0	Ar/Ar	69.0	-	-	(146)
Gd_0.5_La_0.5_Co_0.5_Fe_0.5_O_3_	solid-state	1		1350/1000	5.5/20	40.0	Ar/Ar	125.0	127.0	6.35	(146)
Gd_0.5_La_0.5_Co_0.5_Fe_0.5_O_3_	solid-state	2		1350/1000	5.5/20	40.0	Ar/Ar	93.0	141.0	7.05	(146)
Gd_0.5_La_0.5_Co_0.5_Fe_0.5_O_3_	solid-state	3		1350/1000	5.5/20	40.0	Ar/Ar	87.0	-	-	(146)
LaFe_0.9_Co_0.1_O_3−δ_(25 wt %)/SiO_2_	combustion	1		1350/1100	40/15	100.0	Ar/-	104.2^a^	108.0^a^	7.20	(129)
LaFe_0.9_Co_0.1_O_3−δ_(25 wt %)/SiO_2_	combustion	1		1350/1100	40/60	100.0	Ar/-	104.2^a^	155.1^a^	2.59	(129)
LaFe_0.8_Co_0.2_O_3−δ_(10 wt %)/SiO_2_	combustion	1		1350/1100	40/60	100.0	Ar/-	45.0^a^	57.8^a^	0.96	(129)
LaFe_0.8_Co_0.2_O_3−δ_(25 wt %)/SiO_2_	combustion	1		1350/1100	40/15	100.0	Ar/-	125.2^a^	128.9^a^	8.59	(129)
LaFe_0.8_Co_0.2_O_3−δ_(25 wt %)/SiO_2_	combustion	9		1350/1100	40/60	100.0	Ar/-	125.2^a^	188.3^a^	3.14	(129)
LaFe_0.8_Co_0.2_O_3−δ_(50 wt %)/SiO_2_	combustion	1		1350/1100	40/60	100.0	Ar/-	203.5^a^	351.4^a^	5.86	(129)
LaFe_0.8_Co_0.2_O_3−δ_(75 wt %)/SiO_2_	combustion	1		1350/1100	40/60	100.0	Ar/-	53.6^a^	10.8^a^	0.18	(129)
LaFe_0.7_Co_0.3_O_3−δ_(25 wt %)/SiO_2_	combustion	1		1350/1100	40/15	100.0	Ar/-		117.4^a^	7.83	(129)
LaFe_0.6_Co_0.4_O_3−δ_(25 wt %)/SiO_2_	combustion	1		1350/1100	40/15	100.0	Ar/-		115.4^a^	7.69	(129)
LaFe_0.5_Co_0.5_O_3−δ_(25 wt %)/SiO_2_	combustion	1		1350/1100	40/15	100.0	Ar/-		77.3^a^	5.15	(129)

a Value converted from mL g_material_^–1^. Standard volume occupied: 22400 mL mol^–1^.

### 5.6 Other Perovskite Oxides as Redox-Active Oxygen Exchange Materials

Nickel (Ni) was studied as a B-site element in perovskites. Huijun Zhao’s group elucidated the linear relationship between H_2_ generation and the extent of oxygen vacancy (δ) in La_0.6_Sr_0.4_BO_3_ (B = Mn, Cr, Fe, Co, and Ni). Additionally, the LSB perovskites possessing lower V_o_ formation energies can achieve higher levels of V_o_ formation and oxygen evolution. Notably, the H_2_ production (368 μmol g^–1^) of La_0.6_Sr_0.4_NiO_3−δ_ was found to be competitive when compared to other B-site elements (Cr, Mn, and Fe).^105^ Alejandro Perez et al. also found the promising performance of Ni-based perovskite La_0.8_Al_0.2_NiO_3−δ_ in H_2_ production (196 μmol g^–1^) at an isothermal temperature (800 °C). Furthermore, the La_0.8_Al_0.2_NiO_3−δ_ perovskite demonstrates excellent performance in terms of solar/fuel efficiency with a ratio of 0.46. This ratio represents the potential energy recovery from the produced hydrogen compared to the solar heat required for its production, and it is comparable to or even higher than values reported in the literature for other metal oxides. However, La_0.8_Al_0.2_NiO_3−δ_ perovskites prepared at acidic pH undergo irreversible changes in their crystalline structure during both the thermal reduction and hydrolysis steps.^138^ The perovskite materials La_0.6_Sr_0.4_NiO_3−δ_ were studied with chromium (Cr) as a B-site element. Compared to Mn in the B-site, the Cr-based perovskite exhibits a higher extent of oxygen vacancy formation in the La_0.6_Sr_0.4_B (B = Cr, Mn) system, and the hydrogen production of the Cr-based perovskite is also higher than that of the Mn-based perovskite. This also meets the correlation between the H_2_ production and the extent of oxygen vacancy formation.^105^ Xin Li’s group also designed a Cr perovskite via the oxygen vacancy mechanism analysis and doping-mixture modification for solar thermochemical water-splitting.^147^ Alejandro Perez et al. also investigated Cu in the B-site, La_0.8_Al_0.2_CuO_3−δ_, and it was found that while reducing the temperature to 1400 °C is thermodynamically favorable, the hydrogen production is lower compared to temperatures of 1200 and 1000 °C. Additionally, when comparing the B-site metals Co, Ni, Fe, and Cu, Cu exhibits the lowest hydrogen production at a given temperature.^138^

High-entropy oxides (HEOs), a novel category of materials discovered in 2015, have garnered significant attention in recent years.^148^ A high-entropy oxide is defined as a multicationic oxide featuring a random and homogeneous cation distribution that is entropy-stabilized within a single sublattice. The key criteria for HEO classification include the mixing of typically five or more cations in equimolar amounts (on at least one sublattice) to optimize the configurational entropy and parent oxides exhibiting distinct crystal structures with low mutual solubility.^149^ While most initial studies of HEOs focus on equimolar compositions where the configurational entropy is maximized (ln(*N*)*k*_B_ per cation, where *N* is the number of equimolar components and *k*_B_ is the Boltzmann constant), high-entropy oxides (or ceramics in general) are extended to compositionally complex oxides (CCOs) or ceramics (CCCs) to include nonequimolar compositions, as well as long- and short-range orders, which reduce the configurational entropy but offer more opportunities to tune and improve properties to outperform their equimolar higher-entropy counterparts.^150,151^ The intrinsic configurational disorder within these compounds paves the way for innovative pathways to design and discover multiple new crystalline material phases. In 2018, Jian Luo’s group first reported the fabrication of high-entropy perovskite oxides (HEPOs),^56^ which soon attracted interest because of their catalytic, dielectric, magnetic, ionic, ferroelectric, and other properties with vast tunability. To enhance the efficiency of fuel generation per cycle and achieve favorable kinetic rates, Alex Le Gal et al. delved into an investigation of a series of seven HEOs, including two HEPOs ((Gd_0.2_La_0.2_Nd_0.2_Sm_0.2_Y_0.2_)(Co_0.2_Cr_0.2_Fe_0.2_Mn_0.2_Ni_0.2_)O_3_, (La_0.5_Sr_0.5_)(Mn_0.2_Ce_0.2_Ni_0.2_Mg_0.2_Cr_0.2_)O_3_) and nonequimolar (La_0.8_Sr_0.2_)(Mn_0.2_Fe_0.2_Co_0.4_Al_0.2_)O_3−δ_, to improve the fuel generation with good kinetic rates.^149^ The CO production of these HEPOs is higher than that of ceria but still far from the production yield of LaSrMnO_3_ perovskites.^149^ Dawei Zhang et al. investigated the impact of aliovalent doping, differentiating between normal and abnormal cases, on the redox behaviors observed in compositionally complex perovskite oxides (CCPOs). They discovered two series of medium-entropy CCPOs, namely, (La_1–*x*_Sr_*x*_)(Mn_1/3_Fe_1/3_Ti_1/3_)O_3−δ_ (LS_MFT) and (La_1–*x*_Sr_*x*_)(Mn_1/3_Fe_1/3_Cr_1/3_)O_3−δ_ (LS_MFC). They observed a linear relationship between the extent of reduction (Δδ) and the Sr molar ratio in LS_MFC, as well as a V-shaped correlation between Δδ and the Sr molar ratio in LS_MFT. The explanation is as follows: In LS_MFC, the energy shifts of the Cr–L_2,3_, Mn–L_2,3_, and Fe–L_2,3_ peak toward higher values as *x* increases, indicating elevated oxidation states for Cr, Mn, and Fe, along with decreased oxygen vacancy formation energies. In LS_MFT, the V-shaped curve of Δδ vs *x* is attributed to the stable Ti^4+^ state and the V-shaped dependence of the Mn/Fe valency on *x*.^37^

**Figure 14:**
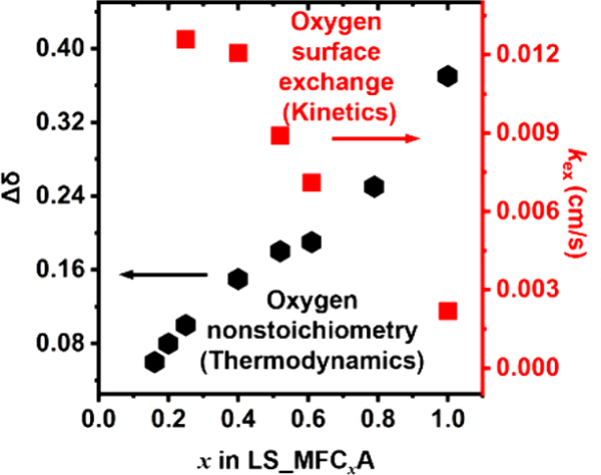
Relationship between Δδ and *K*_ex_ on the Co content in LS_MFC_*x*_A.^27^ Reproduced from ref (27). Copyright 2023 American Chemical Society.

In addition, Zhang et al. studied the STCH performance of (La_0.8_Sr_0.2_)(Mn_(1–*x*)/3_Fe_(1–*x*)/3_Co_*x*_Al_(1–*x*)/3_)O_3_ (LS_MFC_*x*_A) and found that LS_MFC_*x*_A has tunable thermodynamic and kinetics properties. They reported that the extent of reduction (Δδ) increases with the increasing Co content (*x*), but at the same time there is a decreasing trend in the intrinsic kinetics (oxygen surface exchange coefficient, *K*_ex_), as shown in Figure 14.^27^

In B-site elements, the preferential redox element was found to be Co by in situ XPS and TGA. The DFT calculation result shows that oxygen vacancies tend to form near positions where Co is the first nearest neighbor. The analysis of the magnetic moments of the B-site metals also confirms that the Co redox valence change exhibits the highest activity when oxygen vacancies are formed. Furthermore, the bond valence sum (BVS) results indicate a notable weakening of Co–O bonds, slight weakening of Mn–O and Al–O bonds, and strengthening of Fe–O bonds.^27^

Cijie Liu et al. reported a new series of A-site high-entropy HPEOs, (La_1/6_Pr_1/6_Nd_1/6_Gd_1/6_Sr_1/6_Ba_1/6_)MnO_3_ (LPNGSB_Mn), for STCH that show desirable thermodynamic and kinetics properties as well as excellent phase stability and cycling durability. LPNGSB_Mn exhibits enhanced hydrogen production (∼77.5 mmol mol_oxide_^–1^) compared to (La_2/3_Sr_1/3_)MnO_3_ (∼53.5 mmol mol_oxide_^–1^) in a short 1 h redox duration and high STCH production and phase stability for 50 cycles. This work provides a new pathway to explore A-sites with tunable redox, thermodynamic, and kinetics properties of oxides for STCH and chemical looping.^152^Table 10 summarizes the STCH production performance of various perovskite oxides.

**Table 10:** STCH Performance of Selected Other Perovskite Oxides

composition	synthesis method	cycles	Δδ	temp. red./ox. (°C)	time red./ox. (min)	H_2_O (% vol)	gas red./ox.	O_2_ yield (μmol g^–1^)	H_2_ yield (μmol g^–1^)	average H_2_ production rate (μmol g^–1^ min^–1^)	ref
La_0.6_Sr_0.4_NiO_3−δ_	modified Pechini	1	0.200	1300/900	60/60	40.0	Ar/Ar	437.0	368.0	6.13	(105)
La_0.8_Al_0.2_NiO_3−δ_	modified Pechini	5		1400/800	80/60	100.0	N_2_/N_2_		111.61	1.86	(138)
La_0.8_Al_0.2_NiO_3−δ_	modified Pechini	5		1200/800	80/60	100.0	N_2_/N_2_		200.89	3.35	(138)
La_0.8_Al_0.2_NiO_3−δ_	modified Pechini	5		1000/800	80/60	100.0	N_2_/N_2_		380.58	6.34	(138)
La_0.8_Al_0.2_NiO_3−δ_	modified Pechini	5		800/800	80/60	100.0	N_2_/N_2_	94.87	196	3.27	(138)
La_0.6_Sr_0.4_CrO_3−δ_	modified Pechini	1	0.072	1300/900	60/60	40.0	Ar/Ar	166.0	280.0	4.67	(105)
YCr_0.9_Zr_0.1_O_3−δ_	sol–gel	1		1400/1300	/40		Ar/Ar		207.6	5.19	(147)
YCr_0.75_Zr_0.25_O_3−δ_	sol–gel	1		1400/1300	/40		Ar/Ar		219.6	5.49	(147)
YCr_0.6_Zr_0.4_O_3−δ_	sol–gel	1		1400/1300	/40		Ar/Ar		206	5.15	(147)
YCr_0.75_Zr_0.25_O_3−δ_-10 wt % CeO_2_	sol–gel	1		1400/1300	/40		Ar/Ar		209.7	5.24	(147)
YCr_0.75_Zr_0.25_O_3−δ_-25 wt % CeO_2_	sol–gel	1		1400/1300	/40		Ar/Ar		298.7	7.47	(147)
YCr_0.75_Zr_0.25_O_3−δ_-25 wt % CeO_2_	sol–gel	1		1400/1300	40/60		Ar/Ar		449.8	11.25	(147)
YCr_0.75_Zr_0.25_O_3−δ_-50 wt % CeO_2_	sol–gel	1		1400/1300	/40		Ar/Ar		174.6	4.37	(147)
YCr_0.9_Zr_0.1_O_3−δ_	sol–gel	3		1400/1300	/40		Ar/Ar		193.8	4.85	(147)
YCr_0.75_Zr_0.25_O_3−δ_	sol–gel	3		1400/1300	/40		Ar/Ar		210.9	5.27	(147)
YCr_0.6_Zr_0.4_O_3−δ_	sol–gel	3		1400/1300	/40		Ar/Ar		204.3	5.11	(147)
YCr_0.75_Zr_0.25_O_3−δ_-10 wt % CeO_2_	sol–gel	3		1400/1300	/40		Ar/Ar		211.4	5.29	(147)
YCr_0.75_Zr_0.25_O_3−δ_-25 wt % CeO_2_	sol–gel	3		1400/1300	/40		Ar/Ar		256.7	6.42	(147)
YCr_0.75_Zr_0.25_O_3−δ_-50 wt % CeO_2_	sol–gel	3		1400/1300	/40		Ar/Ar		163.7	4.09	(147)
YCr_0.75_Zr_0.25_O_3−δ_-25 wt % CeO_2_	sol–gel	10		1400/1300	/40		Ar/Ar		244.6	6.12	(147)
La_0.8_Al_0.2_CuO_3−δ_	modified Pechini	5		1400/800	80/60	100.0	N_2_/N_2_		22.32	0.37	(138)
La_0.8_Al_0.2_CuO_3−δ_	modified Pechini	5		1200/800	80/60	100.0	N_2_/N_2_		156.25	2.60	(138)
La_0.8_Al_0.2_CuO_3−δ_	modified Pechini	5		1000/800	80/60	100.0	N_2_/N_2_		178.57	2.98	(138)
La_0.8_Al_0.2_CuO_3−δ_	modified Pechini	5		800/800	80/60	100.0	N_2_/N_2_	50.223	93.75	1.56	(138)
(La_0.8_Sr_0.2_)(Mn_0.28_Fe_0.28_Co_0.16_Al_0.28_)O_3−δ_	solid-state	4	0.057	1350/1100	45/15	40.0	N_2_/N_2_		125.0	8.30	(27)
(La_0.8_Sr_0.2_)(Mn_0.267_Fe_0.267_Co_0.20_Al_0.267_)O_3−δ_	solid-state	4	0.081	1350/1100	45/15	40.0	N_2_/N_2_		135.0	9.00	(27)
(La_0.8_Sr_0.2_)(Mn_0.25_Fe_0.25_Co_0.25_Al_0.25_)O_3−δ_	solid-state	4	0.097	1350/1100	45/15	40.0	N_2_/N_2_		162.0	10.80	(27)
(La_0.8_Sr_0.2_)(Mn_0.2_Fe_0.2_Co_0.4_Al_0.2_)O_3−δ_	solid-state	4	0.14	1350/1100	45/15	40.0	N_2_/N_2_		219.0	14.60	(27)
(La_0.8_Sr_0.2_)(Mn_0.16_Fe_0.16_Co_0.52_Al_0.16_)O_3−δ_	solid-state	4	0.18	1350/1100	45/15	40.0	N_2_/N_2_		189.0	12.60	(27)
(La_0.8_Sr_0.2_)(Mn_0.13_Fe_0.13_Co_0.61_Al_0.13_)O_3−δ_	solid-state	4	0.19	1350/1100	45/15	40.0	N_2_/N_2_		148.0	9.87	(27)
(La_0.8_Sr_0.2_)(Mn_0.07_Fe_0.07_Co_0.79_Al_0.07_)O_3−δ_	solid-state	4	0.28	1350/1100	45/15	40.0	N_2_/N_2_		25.0	1.67	(27)
(La_0.8_Sr_0.2_)(Mn_0.2_Fe_0.2_Co_0.4_Al_0.2_)O_3−δ_	solid-state	4	0.14	1350/800	45/15	40.0	N_2_/N_2_		49.0	3.27	(27)
(La_0.8_Sr_0.2_)(Mn_0.2_Fe_0.2_Co_0.4_Al_0.2_)O_3−δ_	solid-state	4	0.14	1350/1000	45/15	40.0	N_2_/N_2_		194.0	12.93	(27)
(La_0.8_Sr_0.2_)(Mn_0.2_Fe_0.2_Co_0.4_Al_0.2_)O_3−δ_	solid-state	4	0.14	1350/1100	30/30	40.0	N_2_/N_2_		270.0	9.00	(27)
(La_0.8_Sr_0.2_)(Mn_0.2_Fe_0.2_Co_0.4_Al_0.2_)O_3−δ_	solid-state	4	0.14	1350/1100	30/60	40.0	N_2_/N_2_		351.0	5.85	(27)
(La_0.8_Sr_0.2_)(Mn_0.2_Fe_0.2_Co_0.4_Al_0.2_)O_3−δ_	solid-state	12	0.14	1350/1100	30/30	40.0	N_2_/N_2_		386.9	12.90	(27)
(La_0.8_Sr_0.2_)(Mn_0.2_Fe_0.2_Co_0.4_Al_0.2_)O_3−δ_	solid-state	51	0.14	1350/1100	30/60	40.0	N_2_/N_2_		157.9	2.63	(27)
(La_1/6_Pr_1/6_Nd_1/6_Gd_1/6_Sr_1/6_Ba_1/6_)MnO_3_	solid-state	1	0.0822	1350/1100	5/20	40.0	N_2_/N_2_		91.9	4.60	(152)
(La_1/6_Pr_1/6_Nd_1/6_Gd_1/6_Sr_1/6_Ba_1/6_)MnO_3_	solid-state	2	0.0822	1350/1100	5/20	40.0	N_2_/N_2_		110.8	5.54	(152)
(La_1/6_Pr_1/6_Nd_1/6_Gd_1/6_Sr_1/6_Ba_1/6_)MnO_3_	solid-state	3	0.0822	1350/1100	5/20	40.0	N_2_/N_2_		126.4	6.32	(152)
(La_1/6_Pr_1/6_Nd_1/6_Gd_1/6_Sr_1/6_Ba_1/6_)MnO_3_	solid-state	4	0.0822	1350/1100	5/20	40.0	N_2_/N_2_		123.5	6.18	(152)
(La_1/6_Pr_1/6_Nd_1/6_Gd_1/6_Sr_1/6_Ba_1/6_)MnO_3_	solid-state	1	0.0822	1350/1100	30/30	40.0	N_2_/N_2_		214.9	7.16	(152)
(La_1/6_Pr_1/6_Nd_1/6_Gd_1/6_Sr_1/6_Ba_1/6_)MnO_3_	solid-state	2	0.0822	1350/1100	30/30	40.0	N_2_/N_2_		240.2	8.01	(152)
(La_1/6_Pr_1/6_Nd_1/6_Gd_1/6_Sr_1/6_Ba_1/6_)MnO_3_	solid-state	3	0.0822	1350/1100	30/30	40.0	N_2_/N_2_		252.8	8.43	(152)
(La_1/6_Pr_1/6_Nd_1/6_Gd_1/6_Sr_1/6_Ba_1/6_)MnO_3_	solid-state	4	0.0822	1350/1100	30/30	40.0	N_2_/N_2_		252.8	8.43	(152)
(La_1/6_Pr_1/6_Nd_1/6_Gd_1/6_Sr_1/6_Ba_1/6_)MnO_3_	solid-state	1	0.0822	1350/900	30/30	40.0	N_2_/N_2_		269.7	8.99	(152)
(La_1/6_Pr_1/6_Nd_1/6_Gd_1/6_Sr_1/6_Ba_1/6_)MnO_3_	solid-state	2	0.0822	1350/900	30/30	40.0	N_2_/N_2_		307.6	10.25	(152)
(La_1/6_Pr_1/6_Nd_1/6_Gd_1/6_Sr_1/6_Ba_1/6_)MnO_3_	solid-state	3	0.0822	1350/900	30/30	40.0	N_2_/N_2_		322.4	10.75	(152)
(La_1/6_Pr_1/6_Nd_1/6_Gd_1/6_Sr_1/6_Ba_1/6_)MnO_3_	solid-state	4	0.0822	1350/900	30/30	40.0	N_2_/N_2_		326.6	10.89	(152)

### 5.7 Discussion on the Role of Inert and Reductant Gases

Inert gases, such as argon and nitrogen, are primarily used to lower the partial pressure of oxygen, thereby facilitating redox reactions in materials.^27,138,152^ The main energy requirements associated with using inert gases include heating the gas to the reaction temperature and separating the generated oxygen afterward.^153^

Reductant gases, such as hydrogen or methane, play an active role in redox reactions, often enhancing oxygen release.^154,155^ When methane is used as the reductant, it prompts the material to release lattice oxygen, which is fully reabsorbed when oxygen is reintroduced at the same temperature.^133^ Additionally, methane consumes the permeated oxygen and produces valuable compounds, such as synthesis gas.^154,155^ Hydrogen is sometimes used in reactions to simulate an up-scale scenario, as high hydrogen concentrations are present during actual experiments. It is also occasionally used as a pretreatment gas, which can affect the crystalline structure of the material.^96^

### 5.8 Comparative Analysis of Perovskite Families and Ceria: Pros, Cons, and Future Development

In this section, we provide a comparative analysis of different perovskite oxide families and ceria, focusing on their advantages and disadvantages, current development status, and potential for overcoming existing challenges, as shown in Table 11.

**Table 11:** Comparative Analysis of Perovskite Oxides and Ceria: Pros and Cons, Development Status, and Future Potential

perovskite family	advantages	disadvantages	development status	potential for overcoming barriers
Mn-based perovskites	high redox activity; significant ionic conductivity; in some systems, Sr doping enhances performance and offers potential for optimization	over doping with Sr negatively impacts reoxidation yield, potential structural changes	extensive research in water-splitting and fuel cells	optimization of doping strategies; development of more stable structures
MnAl-based perovskites	enhanced reoxidation efficiency and an increased extent of reduction	high Sr doping can negatively affect redox activity	research on optimization of Sr, Mn, Al, and Ca; DFT calculations and experimental studies	optimization of doping levels; improvement of structural stability; exploration of new chemical compositions and structures
MnX-based perovskites	high H_2_ yield with Ce, Cr doping; stability under high temperatures and reducing environments	formation of secondary phases	research on the effects of various dopants; development of high-purity synthesis methods	optimization of doping levels and chemical compositions
Fe- and FeX-based perovskites	lower reduction temperature required	sintering and structural changes; presence of secondary phases	research focused on fuel cells and thermochemical cycles; extensive research, particularly on La_1–*x*_Sr_*x*_FeO_3_; studies include Co, Ni, Ir, and Ti dopants	development of antisintering materials; improved thermal stability
Co- and CoX-based perovskites	Co doping enhances oxygen vacancy formation and redox activity	structural changes and aggregation; phase changes at high temperatures	widely used in catalysis and fuel cells; research on various Co-based perovskites and doping strategies	improved stability through doping and protective layers
other perovskites	competitive H_2_ production; tunable redox properties; potential high-entropy stability	irreversible structural changes; lower H_2_ production with Cu dopant	emerging materials research, particularly high-entropy oxides	in-depth study of thermodynamic and kinetic properties; exploration of new compositional spaces
CeO_2_	fast oxidation kinetics; thermal stability	low extent of stoichiometry	extensive doping research; proven performance in large-scale solar reactors	apply doping strategies to address volatilization at both low *P*_O_2__ and high temperatures

Perovskite oxides are emerging as promising materials for STCH due to their high oxygen exchange capacity. Mn-based perovskites exhibit notable redox activity and ionic conductivity, but excessive Sr doping can reduce reoxidation yields. MnAl-based perovskites have enhanced reoxidation efficiencies and an increased extent of reduction but may also suffer from high Sr doping levels, which can negatively affect the redox activity. MnX-based perovskites, doped with Ce and Cr, show high hydrogen yields but face challenges in secondary phase formation. Fe- and FeX-based perovskites, particularly La_1–*x*_Sr_*x*_FeO_3_, are advantageous for their lower reduction temperatures, though issues with sintering and structural changes persist. Co- and CoX-based perovskites are recognized for their high oxygen release and hydrogen production, but cobalt’s toxicity and cost are concerns. Other perovskite types, including high-entropy oxides, offer competitive performance but also face challenges such as irreversible structural changes.

Ceria, on the other hand, has been extensively studied as a mature material for STCH, with significant contributions from Aldo Steinfeld’s group. Their work, particularly on the reticulated porous ceramic (RPC) foam structured CeO_2_, featuring dual-scale porosity and composed entirely of pure CeO_2_, was utilized in a solar cavity-type reactor.^156,157^

Each oxide type has distinct advantages and drawbacks. Mn-based perovskites are highly reactive but suffer from stability issues, while MnAl-based variants show better thermal stability but are complex to synthesize. MnX-based perovskites benefit from additional doping, but they face performance consistency challenges. Fe-based perovskites are cost-effective but typically have lower efficiency in oxygen exchange. Co-based perovskites are stable with high reactivity, though cobalt’s toxicity is a concern. Other perovskites exhibit varied performance characteristics and stability. Ceria, while offering excellent thermal stability, suffers from a low extent of stoichiometry.

While perovskite oxides demonstrate promising properties for STCH applications, it is important to note that current research, including this study, has primarily been conducted under laboratory conditions.^26,27,39,78,152^ In contrast, state-of-the-art materials like CeO_2_ have been successfully tested in upscaled reactor systems powered by concentrated solar energy.^15,34,36,101^ Future research should focus on scaling up the testing of perovskite materials under real-world conditions to better evaluate their performance in practical applications. Additionally, the relatively low steam-to-hydrogen conversion efficiency and incomplete reoxidation yield observed in perovskite oxides highlight the need for further exploration and optimization.^158^ Advances in high-entropy and compositionally complex ceramics offer a promising pathway to enhance the efficiency and stability of STCH materials. The development of new perovskite compositions and the exploration of their performance in large-scale systems will be crucial for realizing the full potential of perovskites in solar thermochemical water-splitting.

## 6 Theoretical Calculations and Modeling Studies

Since Δδ directly impacts the H_2_ production, it is important to evaluate the formation of oxygen vacancies in perovskite materials at both the reduction and oxidation conditions as a guidance. Density functional theory (DFT) calculations have been used to evaluate the oxygen vacancy formation energy. Combining DFT results and thermodynamics connects the DFT-computed vacancy formation energy to STCH performance. Thus, high-throughput DFT calculations can be used to achieve the design of computational materials. Based on the DFT generated data, it becomes possible to develop a machine learning approach to further expand the material search.

The first level of evaluation comes from the DFT computed neutral oxygen vacancy formation energy (Δ*E*_v_^f^). At 0 K,
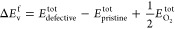
18where *E*_host_^tot^, *E*_defective_^tot^, and *E*_O_2__^tot^ are the total energy of the pristine host structure, that of the O-deficient structure, and that of an oxygen molecule, respectively.^159^ When treating strongly correlated electronic materials, the DFT+U^160^ method is widely adopted to ameliorate the self-interaction inaccuracies that standard DFT has while modestly increasing the computational cost. Without the Hubbard U correction, generalized gradient approximations (GGA), such as PBE and PW91 functionals, can predict simple properties, such as lattice parameters, but can be wrong with electronic properties, magnetic properties, vacancy formation energy, and surface effects.^161^ More advanced functionals, such as PBEsol (a modification of PBE) and the SCAN^162^ meta-GGA functionals ideally should produce more accurate material properties. Table 12 collects the recent DFT computed oxygen vacancy formation energy (Δ*E*_v_^f^) along with the exchange-correlation functional. These DFT studies show that Δ*E*_v_^f^ (*T* = 0 K) is determined by the elements and compositions on both A- and B-sites in ABO_3_, serving as a useful tool for materials design. There are other mechanisms that contribute to the oxygen vacancy formation entropy, including the electronic entropy, which may arise due to the formation of charged oxygen vacancy defects. These emerging calculations will not be fully discussed in this Review. Instead, we will focus on charge-neutral oxygen vacancies. Since the predicted properties for transition metal oxides are sensitive to the DFT calculation details, as shown in Table 12, more caution is needed for high-throughput DFT calculations and DFT-trained machine learning approaches in complex perovskites. Here we summarize them into two sections.

**Table 12:** DFT-Calculated Neutral Oxygen Vacancy Formation Energies

composition							
A	B	supercell size	no. of Vo in a supercell	δ	DFT method	Δ*E*_v_^f^(eV)	ref
La	Mn	80	single	0.0625	DFT+U (GGA-PBE)	3.9	(114)
Sr_0.4_La_0.6_	Mn	80	single	0.0625	DFT+U (GGA-PBE)	2.6	(114)
Sr_0.2_La_0.8_	Mn_0.6_Al_0.4_	80	single	0.0625	DFT+U (GGA-PBE)	3.3^a^	(114)
Sr_0.4_La_0.6_	Mn_0.6_Al_0.4_	80	single	0.0625	DFT+U (GGA-PBE)	2.6	(114)
Sr_0.6_La_0.4_	Mn_0.6_Al_0.4_	80	single	0.0625	DFT+U (GGA-PBE)	1.4	(114)
Sr_0.4_La_0.6_	Mn_0.4_Al_0.6_	80	single	0.0625	DFT+U (GGA-PBE)	2.2	(114)
Sr	Fe	80	single	0.0625	DFT+U (GGA-PBE)	2.102	(170)
Sr_0.875_Ca_0.125_	Fe	80	single	0.0625	DFT+U (GGA-PBE)	2.018	(170)
Sr_0.75_Ca_0.25_	Fe	80	single	0.0625	DFT+U (GGA-PBE)	2.016	(170)
Sr_0.525_Ca_0.375_	Fe	80	single	0.0625	DFT+U (GGA-PBE)	1.98	(170)
Sr_0.5_Ca_0.5_	Fe	80	single	0.0625	DFT+U (GGA-PBE)	2.096	(170)
Sr	Fe	80	multiple	0.1875	DFT+U (GGA-PBE)	2.253	(170)
Sr_0.875_Ca_0.125_	Fe	80	multiple	0.1875	DFT+U (GGA-PBE)	2.084	(170)
Sr_0.75_Ca_0.25_	Fe	80	multiple	0.1875	DFT+U (GGA-PBE)	2.042	(170)
Sr_0.525_Ca_0.375_	Fe	80	multiple	0.1875	DFT+U (GGA-PBE)	1.951	(170)
Sr_0.5_Ca_0.5_	Fe	80	multiple	0.1875	DFT+U (GGA-PBE)	1.957	(170)
Sr	Fe	80	multiple	0.375	DFT+U (GGA-PBE)	2.587	(170)
Sr_0.875_Ca_0.125_	Fe	80	multiple	0.375	DFT+U (GGA-PBE)	2.58	(170)
Sr_075_Ca_0.25_	Fe	80	multiple	0.375	DFT+U (GGA-PBE)	2.476	(170)
Sr_0.525_Ca_0.375_	Fe	80	multiple	0.375	DFT+U (GGA-PBE)	2.484	(170)
Sr_0.5_Ca_0.5_	Fe	80	multiple	0.375	DFT+U (GGA-PBE)	2.532	(170)
Sr	Fe	80	multiple	0.5625	DFT+U (GGA-PBE)	2.862	(170)
Sr_0.875_Ca_0.125_	Fe	80	multiple	0.5625	DFT+U (GGA-PBE)	2.762	(170)
Sr_0.75_Ca_0.25_	Fe	80	multiple	0.5625	DFT+U (GGA-PBE)	2.749	(170)
Sr_0.525_Ca_0.375_	Fe	80	multiple	0.5625	DFT+U (GGA-PBE)	2.749	(170)
Sr_0.5_Ca_0.5_	Fe	80	multiple	0.5625	DFT+U (GGA-PBE)	2.755	(170)
Sr	Co_0.8_Fe_0.2_	40	single	0.125	GGA-PBE	1.58	(171)
Ba_0.5_Sr_0.5_	Co	40	single	0.125	GGA-PBE	1.03	(171)
Ba_0.5_Sr_0.5_	Co_0.75_Fe_0.25_	40	single	0.125	GGA-PBE	1.34	(171)
Ba_0.5_Sr_0.5_	Co_0.5_Fe_0.5_	40	single	0.125	GGA-PBE	1.63	(171)
Ba_0.5_Sr_0.5_	Co_0.25_Fe_0.75_	40	single	0.125	GGA-PBE	1.82	(171)
Ba_0.5_Sr_0.5_	Fe	40	single	0.125	GGA-PBE	2.07	(171)
La	Ga	80	single	0.0625	DFT+U (GGA-PBE)	5.3	(119)
La	Ga_0.75_Co_0.25_	80	single	0.0625	DFT+U (GGA-PBE)	3.0	(119)
La	Ga_0.5_Co_0.5_	80	single	0.0625	DFT+U (GGA-PBE)	2.9	(119)
La	Ga_0.25_Co_0.75_	80	single	0.0625	DFT+U (GGA-PBE)	2.2	(119)
La	Co	80	single	0.0625	DFT+U (GGA-PBE)	2.9	(119)
La_0.875_Sr_0.125_	Ga_0.5_Co_0.5_	80	single	0.0625	DFT+U (GGA-PBE)	1.5^a^	(119)
La_0.75_Sr_0.25_	Ga_0.5_Co_0.5_	80	single	0.0625	DFT+U (GGA-PBE)	0.7	(119)
La_0.5_Sr_0.5_	Fe	40	multiple	0.125	DFT+U (GGA-PW91)	0.3992	(172)
La_0.5_Sr_0.5_	Fe_0.5_Co_0.5_	40	multiple	0.125	DFT+U (GGA-PW91)	0.3255	(172)
La_0.5_Sr_0.5_	Co	40	multiple	0.125	DFT+U (GGA-PW91)	0.2483	(172)
La_0.5_Sr_0.5_	Fe	40	multiple	0.25	DFT+U (GGA-PW91)	0.8276	(172)
La_0.5_Sr_0.5_	Fe_0.5_Co_0.5_	40	multiple	0.25	DFT+U (GGA-PW91)	0.6687	(172)
La_0.5_Sr_0.5_	Co	40	multiple	0.25	DFT+U (GGA-PW91)	0.5553	(172)
La_0.5_Sr_0.5_	Fe	40	multiple	0.375	DFT+U (GGA-PW91)	1.2299	(172)
La0.5Sr0.5	Fe_0.5_Co_0.5_	40	multiple	0.375	DFT+U (GGA-PW91)	1.0375	(172)
La0.5Sr0.5	Co	40	multiple	0.375	DFT+U (GGA-PW91)	0.8712	(172)
Ca	Mn	20	single	0.25	DFT (GGA-PW91)	1.47	(173)
Ca	Co_0.25_Mn_0.75_	20	single	0.25	DFT (GGA-PW91)	0.60	(173)
Ca	Co_0.5_Mn_0.5_	21	single	0.25	DFT (GGA-PW91)	0.47	(173)
Ca	Co_0.75_Mn_0.25_	22	single	0.25	DFT (GGA-PW91)	0.38	(173)
Ca	Co	23	single	0.25	DFT (GGA-PW91)	0.36	(173)
La	Fe	80	single	0.0625	DFT+U (GGA-PBE)	4.98	(163)
La	Co	80	single	0.0625	DFT+U (GGA-PBE)	4.02	(163)
Sr	Fe	40	single	0.125	DFT+U (GGA-PBE)	2.34	(163)
Sr	Co	40	single	0.125	DFT+U (GGA-PBE)	1.30	(163)
Ba	Ti	40	single	0.125	PBEsol	5.18^a^	(164)
Ba	V	40	single	0.125	PBEsol	4.46^a^	(164)
Ba	Cr	40	single	0.125	PBEsol	3.42	(164)
Ba	Mn	40	single	0.125	PBEsol	1.35^a^	(164)
Ba	Fe	40	single	0.125	PBEsol	1.30^a^	(164)
Ba	Co	40	single	0.125	PBEsol	0.52^a^	(164)
Ba	Ni	40	single	0.125	PBEsol	–0.41^a^	(164)
Ba	Cu	40	single	0.125	PBEsol	–1.04^a^	(164)
Ca	Ti	40	single	0.125	SCAN+U	6.48	(124)
Ca	V	40	single	0.125	SCAN+U	4.18	(124)
Ca	Cr	40	single	0.125	SCAN+U	2.25	(124)
Ca	Mn	40	single	0.125	SCAN+U	2.25	(124)
Ca	Fe	40	single	0.125	SCAN+U	0.80^a^	(124)
Ca	Co	40	single	0.125	SCAN+U	–1.37	(124)
Ca	Ni	40	single	0.125	SCAN+U	1.20^a^	(124)
Ce	Sc	40	single	0.125	SCAN+U	6.70^a^	(124)
Ce	V	40	single	0.125	SCAN+U	6.30^a^	(124)
Ce	Cr	40	single	0.125	SCAN+U	5.22	(124)
Ce	Co	40	single	0.125	SCAN+U	3.68	(124)
Ce	Ni	40	single	0.125	SCAN+U	4.25	(124)
Ca_0.5_Ce_0.5_	Sc	40	single	0.125	SCAN+U	4.40^a^	(124)
Ca_0.5_Ce_0.5_	Ti	40	single	0.125	SCAN+U	6.30^a^	(124)
Ca_0.5_Ce_0.5_	V	40	single	0.125	SCAN+U	5.00^a^	(124)
Ca_0.5_Ce0_.5_	Cr	40	single	0.125	SCAN+U	4.80^a^	(124)
Ca_0.5_Ce_0.5_	Mn	40	single	0.125	SCAN+U	3.65^a^	(124)
Ca_0.5_Ce_0.5_	Fe	40	single	0.125	SCAN+U	3.77^a^	(124)
Ca_0.5_Ce_0.5_	Co	40	single	0.125	SCAN+U	–0.1^a^	(124)
Ca_0.5_Ce_0.5_	Ni	40	single	0.125	SCAN+U	0.2^a^	(124)

a Values obtained from plot.

### 6.1 Oxygen Vacancy Formation Energy in Simple ABO_3_ Compositions

The effect of A elements and/or B elements on the oxygen vacancy formation energy was evaluated by comparing different simple ABO_3_ perovskites using DFT calculations. Due to the simplicity of these structures, high-throughput calculations are possible.

Jia et al. calculated the *E*_v_^f^ of ABO_3−δ_ (A = La, Sr, and B = Fe, Co).^163^ They obtained that Δ*E*_v_^f^ is in the order of LaFeO_3_ > LaCoO_3_ > SrFeO_3_ > SrCoO_3_. It should be noted that the 80-atom supercell was used for LaBO_3_, while the 40-atom supercell was used for SrFeO_3_.

Ghose et al. studied the effect of the B-site element in BaMO_3_ (M = Ti–Cu).^164^ Δ*E*_v_^f^ decreases as this electronic structure transitions from semiconducting character (BaTiO_3_, BaVO_3_) to ferromagnetic character (BaCrO_3_, BaMnO_3_, and BaFeO_3_) and ultimately metallic character (BaCoO_3_, BaNiO_3_, and BaCuO_3_). The decreasing trend indicates a progressive weakening of the M–O bond, which, in turn, enhances the reduction of M^4+^. The trend was rationalized in terms of their electronic band structures and partial densities of states, which are related to the charge transfer.

Gautam et al. compared the Δ*E*_v_^f^ of CaMO_3–0.125_, CeMO_3–0.125_, and Ca_0.5_Ce_0.5_MO_3–0.125_, where M = Sc, Ti, V, Cr, Mn, Fe, Co, and Ni, using SCAN+U.^124^ They correlated the *E*_v_^f^ to the standard reduction potentials (versus the standard hydrogen electrode, SHE). It was found that the trends in standard reduction potentials are the dominant criteria indictating the monatomic decrease of Δ*E*_v_^f^ in CaMO_3–0.125_ and CeMO_3–0.125_ as M varies from Ti to Co (the higher standard reduction potentials, the lower the *E*_v_^f^). In Ca_0.5_Ce_0.5_MO_3–0.125_, however, structural and electronic factors play a larger role in determining Δ*E*_v_^f^.

Curnan et al. evaluated the effects of concentration, crystal structure, magnetism, and electronic structure on the Δ*E*_v_^f^ of ABO_3_ (A = La, Sr, Ba, K, Na and B = Sc, Ti, V, Cr, Mn, Fe, Co, Ni, Cu).^165^ It was shown that the higher Δ*E*_v_^f^ was observed in La-based ABO_3_ when comparing A-site elements, and the cubic structure has a lower Δ*E*_v_^f^ compared to the rhombohedral structure (LaBO_3_) and to the orthorhombic structure (SrBO_3_). They also showed that trends in Δ*E*_v_^f^ are largely unaffected by the supercell size; however, the value of *E*_v_^f^ varies by more than 1 eV between the 1 × 1 × 1 supercell and the 2 × 2 × 2 supercell (for example, LaCuO_3_, SrCrO_3_, SrMnO_3_, and SrFeO_3_), indicating vacancy–vacancy interaction. They have focused on characterizing the relative ordering in energetic trends.

**Figure 15:**
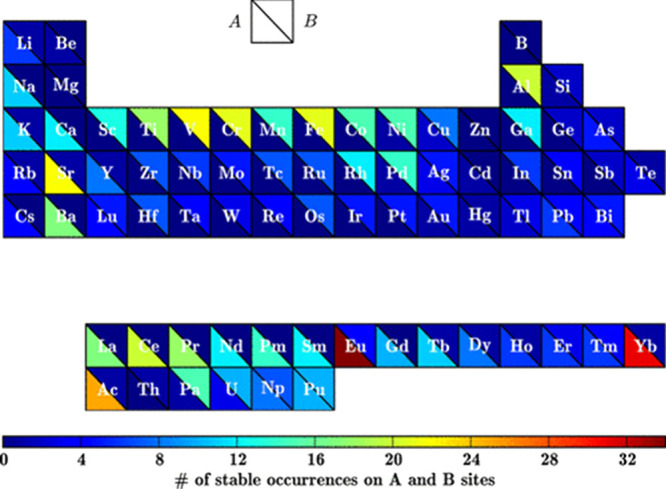
High-throughput density functional theory analysis of 5329 cubic and distorted perovskite ABO_3_ compounds for screening thermodynamically suitable materials for two-step thermochemical water-splitting. Stability of ABO_3_ perovskites was assessed by the frequency of A- and B-site cations: a periodic table highlights all elements considered for A and B sites. Elements are color-coded according to their frequency of stable occurrences on either the A- or B-site.^166^ Reproduced from ref (166). Copyright 2016 American Chemical Society.

Emery et al. investigated 5329 compounds in a high-throughput manner, as presented in Figure 15.^166^ They first reported 383 thermodynamically stable compounds based on the stability of a compound with respect to all other phases present in the A–B–O phase diagram (Δ*H*_stab_^ABO_3_^ < 25 meV/atom). Then, the Δ*E*_v_^f^ was calculated for the predicted 383 compounds and 139 compounds that fall in the target Δ*E*_v_^f^ window (2.5 < Δ*E*_v_^f^/(O atom) < 5 eV). For the defect calculations, extremely small 9-atom (A_2_B_2_O_5_) supercells were used to reduce the computational cost.

Deml et al. further related the DFT-computed Δ*E*_v_^f^ values for 45 oxides to features such as the strength of the metal–oxygen bonds, the energy needed to transfer the electrons left behind by the neutral O vacancy, and the charge transfer between chemically bonded O and metal atoms with a linear relation^167^ approach. This machine learning model can be used to predict Δ*E*_v_^f^ values accurately (with an error of 0.39 eV in a subset of oxides in the NRELMatDB database) without the cost of DFT calculations.

Wexler et al. proposed a machine learning linear model based on SCAN+U that obtained Δ*E*_v_^f^ values of ABO_3_ perovskites, where A = Ca, Sr, Ba, La, Ce and B = Ti, V, Cr, Mn, Fe, Co, Ni, in six lattice systems.^168^ They took crystal bond dissociation energies, crystal reduction potentials, band gaps, and energies above the convex hull as inputs and demonstrated that the model prevailed over the one presented by Deml et al. Its ability in material discovery was presented by predicting BiFeO_3_ and BiCoO_3_ as possible STCH candidates.

Baldassarri et al. discussed the importance of using DFT ground-state structures instead of a dynamically unstable structure that was observed by experiment, since Δ*E*_v_^f^ is very sensitive to the structure used in computation.^169^ They applied this approach to revise and expand their high-throughput framework for STCH ABO_3_ material screening and reduced the error from 0.86 to 0.54 eV. They predicted Δ*E*_v_^f^ values for ∼2200 compositions and found ∼180 compounds within the STCH window (2–5 eV). Based on the appearance frequency in the lower end of the STCH window, Mn^4+^, Mn^3+^, and Co^3+^ were identified as the more promising redox-active B cations for STCH applications.

### 6.2 Effect of A- or B-Site Doping on Oxygen Vacancy Formation Energy

In practice, most perovskite oxides used for STCH contain either A-site mixing, B-site mixing, or both. Computing the oxygen vacancy formation energy with mixed A- and/or B-sites requires larger structures and mixed sites.

**Figure 16:**
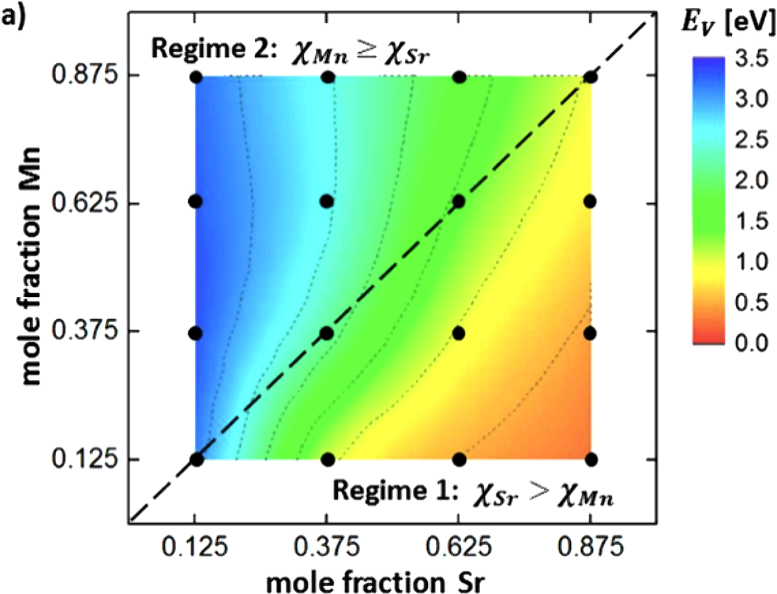
Relationship between composition and oxygen vacancy formation energies (Δ*E*_v_^f^) in Sr_*x*_La_1–*x*_Mn_*y*_Al_1–*y*_O_3–0.0625_ (*x*, *y* = 0.2, 0.4, 0.6, and 0.8).^114^ Reproduced from ref (114). Copyright 2014 American Chemical Society.

Deml et al. investigated the composition dependency of Δ*E*_v_^f^ in Sr_*x*_La_1–*x*_Mn_*y*_Al_1–*y*_O_3–0.0625_ (*x*, *y* = 0.2, 0.4, 0.6, and 0.8), as shown in Figure 16.^114^ The Δ*E*_v_^f^ value decreases as the Sr content increases. When *x* > *y*, increases in Mn contents result in an increase of Δ*E*_v_^f^, while Δ*E*_v_^f^ is approximately constant as the Mn contents increase for *y* > *x*. It was concluded that Sr_0.4_La_0.6_Mn_0.6_Al_0.4_O_3_ has a *E*_v_^f^ = 2.6 eV predicted by DFT calculation and is the optimal composition based on the design parameters including redox thermodynamics, kinetics, utilized redox capacity, and material stability.

Jia et al. studied the effect of Ca^2+^ doping in Sr_1–*x*_Ca_*x*_FeO_3–0.0625_ (*x* = 0, 0.125, 0.25, 0.375, 0.5).^170^ It was shown that the Δ*E*_v_^f^ has its minimum value at *x* = 0.375 and the dominant mechanism behind this is a change of the Fe–O. For *x* = 0–0.375, the Δ*E*_v_^f^ decreases due to the shortening of Fe–O bonds through Ca^2+^ doping, while for *x* = 0.375–0.5 it increases since the Fe–O bonds increase in length to a more stable length due to the FeO_6_ octahedron distortion. They also calculated the Δ*E*_v_^f^ for Sr_1–*x*_Ca_*x*_FeO_3−δ_, where δ = 0.1875, 0.375, and 0.5625, by removing 3, 6, and 9 O atoms from the 80-atom supercell randomly. The trend of Δ*E*_v_^f^ decreasing first and then increasing upon Ca^2+^ doping is unchanged for those cases; however, a vacancy–vacancy interaction has not been explored.

Merkle et al. analyzed the dependence of the Δ*E*_v_^f^ on the Fe content in Ba_1–*x*_Sr_*x*_Co_1–*y*_Fe_*y*_O_3–0.125_.^171^ The Δ*E*_v_^f^ linearly increases with increasing Fe content, which was rationalized based on the calculated electronic density of states, with unoccupied Fe states located higher in energy than Co states. Fe exhibits a peak about 1 eV higher than the Fermi level, whereas Co shows a considerable density of states close to the Fermi level so that Co^4+^ can easily is reduced to Co^3+^.

Chen et al. demonstrated that doping of Sr or Co in A- or B-site, respectively, is useful to reduce the Δ*E*_v_^f^ in La_1–*x*_Sr_*x*_Ga_1–*y*_Co_*y*_O_3–0.0625_ because the metal oxygen bonds (M–O) are weakened after doping.^119^ They experimentally found that undoped LaGaO_3_ is inactive for water-splitting and thus doping Co makes it redox active.

Maiti et al. showed a systematic trend of Δ*E*_v_^f^: the value of Δ*E*_v_^f^ is smaller as the Sr content increases in the A-site and as the Fe content decreases in the B-site in La_1–*x*_Sr_*x*_Fe_1–*y*_Co_*y*_O_3−δ_ (δ = 0.125, 0.25, 0.375).^172^ They also found that oxygen removal from the La layer is more favorable than that from a Sr layer due to the d orbitals in La and the smaller ionic radius. The Δ*E*_v_^f^ values with δ = 0.125, 0.25, and 0.375 were calculated by removing 1, 2, and 3 atoms of the 40-atom supercell. A vacancy–vacancy interaction has not been included even though the 40-atom supercell is small.

Jin et al. investigated the Δ*E*_v_^f^ of Co-doped CaCo_*x*_Mn_1–*x*_O_3−δ_ with different Co content levels (*x* = 0–1).^173^ It was shown that even with a small amount of Co-doping (*x* = 0.25), the value of Δ*E*_v_^f^ is reduced by almost half (1.47 to 0.60 eV). As the Co content increases further, Δ*E*_v_^f^ decreases so that the oxygen release process is facilitated. However, it should be noted that the 2 × 2 × 1 supercell (20 atoms) is very small, vacancy–vacancy interaction is likely in such a small cell.

Witman et al. presented a graph neural network model applicable to arbitrary structures to predict vacancy formation enthalpies.^153^ The model was trained with DFT data from all seven crystal structure systems, and the authors showed its ability to handle mixed ABO_3_ systems by cross validating (Ba_*x*_A_1–*x*_)MnO_3_ (A = Ce, Nb, Pr) and Sr_1–*x*_Ce_*x*_MnO_3_ with DFT. The prediction error of this more generalizable model is below 0.45 eV, which is comparable to the model from Demel or Wexler.^114,168^ In most of these studies, a vacancy–vacancy interaction has been ignored. In detail, most of the studies investigated the formation of single neutral oxygen vacancy.^114,119,124,163−166,171,173^ Several studies used a small cell size,^166,173^ where the vacancy in the simulation cell and the vacancy in the image cells are likely to interact. Some have shown cell size (or concentration) dependent Δ*E*_v_^f^,^165^ and others have explored multiple vacancies in a supercell, but these studies have not discussed the vacancy–vacancy interaction.^170,172^ Das et al. showed oxygen vacancies start to interact around δ ∼ 0.05, and this interaction becomes stronger when δ > 0.1 in SrFeO_3−δ_ and La_0.5_Sr_0.5_FeO_3−δ_.^159,174^ It was shown that long-range charge transfer results in a large oxygen vacancy polaron size, which leads to a significant increase of *E*_v_^f^ when δ > 0.1. Mixed cations on A- and/or B-sites will significantly increase the search space. Vieten et al. calculated the perovskite to brownmillerite reaction enthalpy as an indication of the oxygen vacancy formation energy with at most two different elements mixed at both A- and B-sites.^175^ To reach a better agreement with experiments, they had to consider vacancy interactions by introducing δ-dependent Δ*H*. A high-entropy perovskite was modeled recently for a different application; chemical looping involves oxygen vacancy reactions with CO_2_. Wang et al. first applied high-throughput DFT calculations for the Sr_*x*_A_1–*x*_Fe_*y*_B_1–*y*_O_3−δ_ perovskite at various oxygen nonstoichiometries and different A (alkaline earth, alkali, or rare earth metals) and B (transition metals).^176^ These DFT results were used to train a machine-learned random forest model to investigate the redox thermodynamics of 227 and 273 perovskites with 5 cation elements. Validation with experiments of the predicted compositions gave satisfactory results, but the authors admitted that their DFT model could not cover complicated spin couplings and electronic states in systems containing more than 5 cation elements. Detailed DFT calculations combined with Monte Carlo sampling have been used to investigate the oxygen vacancy formation in LaSr{CoFeMnCo}O_3−δ_. It was revealed that Co is the redox-active B-site element (agreeing with experiments), mainly due to the elongated bonds around Co in the high-entropy perovskite.^27^

### 6.3 Directly Predicting the Δδ for STCH Performance

To compare the DFT-computed oxygen vacancy formation energy at 0 K with the experimentally measurable properties under the oxygen gas at a given temperature (*T*) and partial pressure of oxygen (*P*_O_2__), the oxygen vacancy formation free energy (Δ*G*_v_^f^(*T*, *P*_O_2__)) can be employed and is calculated as
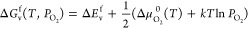
19where Δμ_*O*_2__^0^(*T*) is the change in the chemical potential of oxygen from 0 K to *T* and *k* is the Boltzmann constant.

In ABO_3−δ_, δ is the oxygen nonstoichiometry and the site fraction of oxygen vacancies (*X*) is given by *X* = δ/3. If the oxygen vacancy formation energy is independent of the oxygen nonstoichiometry (dilute scenario), the oxygen nonstoichiometry (δ) is typically described by^159,177^
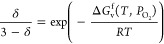
20With increasing δ, Δ*G*_v_^f^ starts to increase with δ, due to vacancy–vacancy interaction, in a nondilute scenario, which is commonly observed in perovskites.^178,179^ So, the right-hand side of eq 20 is modified as
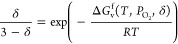
21Qian et al. further simplified Δ*G*_v_^f^ as a linear function of δ and fitted it to experimental data to determine both the enthalpy and entropy of oxygen vacancy formation.^178,179^ Das et al. also fitted DFT-computed Δ*E*_v_^f^ as a linear function of δ and used eq 21 to predict the δ in SrFeO_3−δ_ and La_0.5_Sr_0.5_FeO_3−δ_.^159,174^ They demonstrated that the increase of the vacancy formation energy with the vacancy concentration must be included to predict the δ measured in the experiments. In perovskites with mixed cations, especially in high-entropy or compositionally complex perovskite oxides, the oxygen vacancy formation energy is no longer a single value but rather has a distribution. Recently, Park et al. introduced distributed Δ*E*_v_^f^ for eqs 18 –21 and predicted Δδ under the STCH reduction and oxidation conditions. By comparing dry TGA data, they illustrated that both the oxygen vacancy interaction and the vacancy formation energy distribution must be considered to predict the experimental trend.^135^

As described above, the chemical potential of oxygen (μ_O_) varies with temperature and the oxygen partial pressure under dry conditions. Under wet conditions, the oxygen partial pressure is determined by the partial pressure of H_2_O steam and the partial pressure of H_2_ gas in a reactor.^91,180,181^ Accordingly, μ_O_ changes with various testing conditions such as temperature, the gas composition in the reactor, and the oxygen nonstoichiometry. Thus, diverse experimental conditions can be unified through μ_O_, which is beneficial for a fair comparison of the H_2_ yields under different temperatures and steam concentrations.

## 7 Conclusions, Challenges, and Perspectives

Two-step solar-driven thermochemical hydrogen (STCH) is a promising renewable fuel technology that efficiently stores and converts solar light to hydrogen fuel. The oxygen storage capacity (OSC) plays a crucial role in assessing the suitability of metal oxides for thermochemical processes. Ideal metal oxides for STCH reactions exhibit a large redox extent, favorable thermodynamic properties (such as reduction of entropy and enthalpy), fast reaction kinetics, high resistance to sintering, and thermal stability. These characteristics contribute to efficient and reliable STCH processes.

Perovskite oxides, with their exceptional properties, have captivated researchers and sparked investigations of their potential for solar-to-hydrogen (H_2_) conversion. The structural stability of perovskites enables them to accommodate significant oxygen vacancies during solar-to-fuel conversion. Their tunability also allows for the incorporation of various chemical elements, providing numerous possibilities for future investigations. This flexibility in tuning thermodynamic parameters makes perovskite materials ideal for designing favorable systems for STCH applications. Moreover, perovskites have the advantage of requiring lower reaction temperatures for reduction and offering a larger possible change of nonstoichiometry compared to ceria. Additionally, perovskites not only are promising for water-splitting but also show potential for CO_2_ thermochemical reduction. Their ability to undergo redox reactions at high temperatures makes them suitable for both applications. The same properties that make perovskites effective for H_2_O activation, such as the ability to accommodate multiple oxidation states and the presence of oxygen vacancies, are also beneficial for CO_2_ reduction.

However, it should be noted, that unlike ceria, perovskite materials are still primarily at the laboratory scale and have not been demonstrated in upscaled reactor systems. The primary challenge faced by perovskites is the low efficiency of the solar-to-fuel conversion. Therefore, the next research direction is to search for novel perovskites with a higher efficiency that can yield greater hydrogen production in water-splitting reactions. Furthermore, another limitation associated with perovskite utilization is the incomplete reoxidation yield, resulting from low kinetics and insufficient thermodynamic driving forces. Additionally, high-entropy oxides, which consist of multiple principal elements, exhibit high configurational entropy. These materials demonstrate remarkable properties, such as stabilizing their structure at high temperatures, tunable water-splitting properties, and potential utilization for hydrogen production.

This study has compiled information about all perovskites used in STCH and has summarized various methods employed to investigate reaction kinetics and thermodynamics concerning nonstoichiometric perovskites, providing valuable insights into the diverse available techniques, along with their pros, cons, and related performance data. These characterizations are beneficial for identifying suitable perovskites with favorable thermodynamics and rapid reaction kinetics. Additionally, the crystallographic stability of perovskites over thermochemical cycling is also an important factor that affects the performance of solar thermochemical water-splitting. It is important to note that the B-site in perovskite materials is likely a key active site for H_2_O activation; however, this hypothesis requires further validation, offering a promising avenue for future research.

Overall, the future prospects of perovskites in solar thermochemical water-splitting show considerable potential. However, the search for perovskites with high solar-to-fuel efficiency, favorable thermodynamics, fast kinetic properties, and crystallographic stability.

Recent advancements in high-entropy and compositionally complex ceramics open additional opportunities to tune the thermodynamic and kinetic properties of STCH materials via their vast, unexplored compositional space. These have been demonstrated in a few recent studies of HEPOs and CCPOs in the perovskite structure (without phase transformation during the STCH cycling),^27,158^ as well as an equimolar polycation (Fe_0.25_Mg_0.25_Co_0.25_Ni_0.25_)O_*x*_ in the rocksalt/spinel structure (with phase transformation during STCH cycling).^182^ In addition to the vast compositional spaces, compositionally complex ceramics (CCCs) also offer new mechanisms, e.g., using the distribution of oxygen vacancy formation enthalpies to tune STCH performance.^27,135^ The benefits and new opportunities brought about by CCCs may also be extended to other oxides. For example, we can envision the tuning of oxygen vacancies and thereby STCH performance in ceria-based (or ceria-containing or even ceria-free) compositionally complex fluorite-based oxides (CCFBOs) that have been fabricated with a variety of compositions in the fluorite or fluorite-derived structure with long- and short-range order but not yet optimized for STCH performance.^151,183−187^
